# Nucleolin promotes angiogenesis and endothelial metabolism along the oncofetal axis in the human brain vasculature

**DOI:** 10.1172/jci.insight.143071

**Published:** 2023-04-24

**Authors:** Marc Schwab, Ignazio de Trizio, Moheb Ghobrial, Jau-Ye Shiu, Oguzkan Sürücü, Francesco Girolamo, Mariella Errede, Murat Yilmaz, Johannes Haybaeck, Alessandro Moiraghi, Philippe P. Monnier, Sean E. Lawler, Jeffrey P. Greenfield, Ivan Radovanovic, Karl Frei, Ralph Schlapbach, Viola Vogel, Daniela Virgintino, Katrien De Bock, Thomas Wälchli

**Affiliations:** 1 Group of CNS Angiogenesis and Neurovascular Link, Neuroscience Center Zurich, and Division of Neurosurgery, University and University Hospital Zurich, Swiss Federal Institute of Technology (ETH) Zurich, Zurich, Switzerland.; 2 Division of Neurosurgery, University Hospital Zurich, Zurich, Switzerland.; 3 Group of Brain Vasculature and Perivascular Niche, Division of Experimental and Translational Neuroscience, Krembil Brain Institute, Krembil Research Institute, Toronto Western Hospital, University Health Network, University of Toronto, Toronto, Ontario, Canada.; 4 Division of Neurosurgery, Department of Surgery, Toronto Western Hospital, Toronto, Ontario, Canada.; 5 Institute for Regenerative Medicine (IREM), University of Zurich, Schlieren, Switzerland.; 6 Department of Basic Medical Sciences, Neurosciences and Sense Organs, University of Bari School of Medicine, Bari, Italy.; 7 Department of Neurosurgery, Neurocenter of Southern Switzerland, Regional Hospital Lugano, Lugano, Switzerland.; 8 Laboratory of Exercise and Health, Institute of Exercise and Health, and; 9 Laboratory of Applied Mechanobiology, Department of Health Sciences and Technology, Swiss Federal Institute of Technology Zurich, Zurich, Switzerland.; 10 Graduate Institute of Biomedical Sciences, China Medical University, Taichung, Taiwan.; 11 Center for Psychiatry Emmendingen, Emmendingen, Switzerland.; 12 Department of Internal Medicine, Cantonal Hospital, Lucerne, Switzerland.; 13 Department of Pathology, Neuropathology, and Molecular Pathology, Medical University of Innsbruck, Innsbruck, Austria.; 14 Diagnostic & Research Center for Molecular Biomedicine, Institute of Pathology, Medical University of Graz, Graz, Austria.; 15 Division of Neurosurgery, Department of Clinical Neurosciences, University Hospital Geneva, Geneva, Switzerland.; 16 Krembil Research Institute, Vision Division, Krembil Discovery Tower, Toronto, Ontario, Canada.; 17 Department of Physiology, Faculty of Medicine, and; 18 Department of Ophthalmology and Vision Sciences, Faculty of Medicine, University of Toronto, Ontario, Toronto, Canada.; 19 Harvey Cushing Neuro-Oncology Laboratories, Department of Neurosurgery, Harvard Medical School, Brigham and Women’s Hospital, Boston, Massachusetts, USA.; 20 Department of Pathology and Laboratory Medicine, Legoretta Cancer Center, Brown University, Providence, Rhode Island, USA.; 21 Department of Neurological Surgery, Weill Cornell Medical College, New York, New York, USA.; 22 Functional Genomics Center Zurich, ETH Zurich and University of Zurich, Zurich, Switzerland.

**Keywords:** Angiogenesis, Neuroscience, Brain cancer, Glucose metabolism, Neurodevelopment

## Abstract

Glioblastomas are among the deadliest human cancers and are highly vascularized. Angiogenesis is dynamic during brain development, almost quiescent in the adult brain but reactivated in vascular-dependent CNS pathologies, including brain tumors. The oncofetal axis describes the reactivation of fetal programs in tumors, but its relevance in endothelial and perivascular cells of the human brain vasculature in glial brain tumors is unexplored. Nucleolin is a regulator of cell proliferation and angiogenesis, but its roles in the brain vasculature remain unknown. Here, we studied the expression of Nucleolin in the neurovascular unit in human fetal brains, adult brains, and human gliomas in vivo as well as its effects on sprouting angiogenesis and endothelial metabolism in vitro. Nucleolin is highly expressed in endothelial and perivascular cells during brain development, downregulated in the adult brain, and upregulated in glioma. Moreover, Nucleolin expression correlated with glioma malignancy in vivo. In culture, siRNA-mediated Nucleolin knockdown reduced human brain endothelial cell (HCMEC) and HUVEC sprouting angiogenesis, proliferation, filopodia extension, and glucose metabolism. Furthermore, inhibition of Nucleolin with the aptamer AS1411 decreased brain endothelial cell proliferation in vitro. Mechanistically, Nucleolin knockdown in HCMECs and HUVECs uncovered regulation of angiogenesis involving VEGFR2 and of endothelial glycolysis. These findings identify Nucleolin as a neurodevelopmental factor reactivated in glioma that promotes sprouting angiogenesis and endothelial metabolism, characterizing Nucleolin as an oncofetal protein. Our findings have potential implications in the therapeutic targeting of glioma.

## Graphical abstract

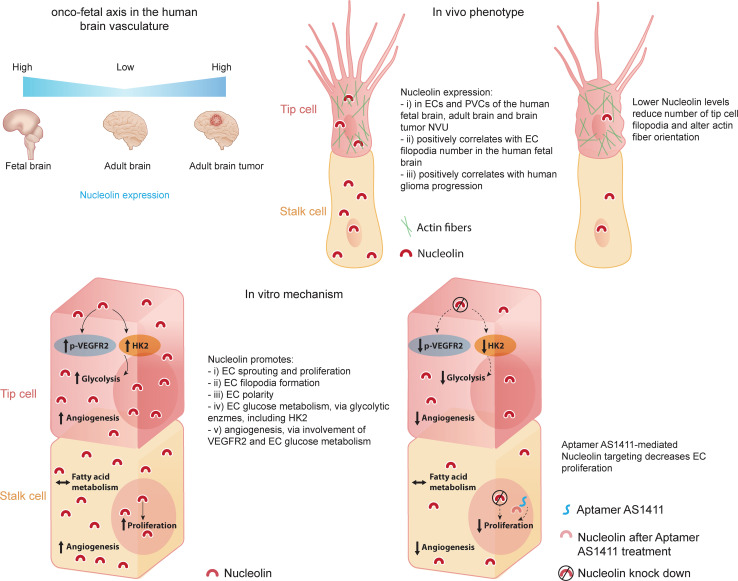

## Introduction

Glioblastomas (GBMs) are among the deadliest human cancers, with a less than 15-month median survival and a 5-year survival of only 5% (1–3) (Supplemental Introduction; supplemental material available online with this article; https://doi.org/10.1172/jci.insight.143071DS1).

A typical feature of GBMs is their high grade of vascularization established by angiogenesis, the growth of new blood vessels (4–6). GBM growth is highly dependent on angiogenesis and mutual interaction among the cellular components of the neurovascular unit (NVU)/perivascular niche (PVN), including endothelial and perivascular cells such as pericytes, astrocytes, neurons, macrophages, microglia, and neuronal stem cells (7). Accordingly, therapeutic approaches targeting angiogenesis and the NVU in GBM have been proposed (1, 8–10). However, despite promising preclinical data, antiangiogenic agents have failed to show a survival benefit in randomized controlled trials in patients with GBM (1, 8). This is mainly due to our limited knowledge about the cellular and molecular mechanisms regulating angiogenesis and the NVU in brain tumors (1, 4, 5, 7, 11, 12).

Whereas angiogenesis is highly dynamic during brain development, the brain vasculature is mostly quiescent in the adult brain, with only few proliferating endothelial cells (ECs) ensuring a stable blood-brain barrier (BBB) (11, 13–16). Interestingly, angiogenesis and the NVU are reactivated in a variety of angiogenesis-dependent central nervous system (CNS) pathologies such as brain tumors, brain vascular malformations, or stroke (4, 7, 11, 14, 17). However, the molecular signaling cascades reactivated in brain tumors remain elusive. For instance, whether there is molecular similarity between developmental and tumor angiogenesis (termed oncofetal axis; refs. 18–25) and how neurodevelopmental pathways regulate brain tumor (vessel) growth remain poorly defined. Thus, in order to better understand pathological brain tumor vasculature, a molecular understanding of normal vascular brain development is crucial (11, 13, 17).

During development, the brain vascular network is established during embryogenesis and at the postnatal stage (11, 26). After initial formation of the perineural vascular plexus (PNVP) surrounding the CNS via vasculogenesis (de novo formation of blood vessels from angioblasts), the brain is predominantly vascularized by sprouting angiogenesis (formation of new blood vessels from preexisting ones) (11, 26). During sprouting angiogenesis, endothelial tip cells (ETCs) at the forefront of vascular sprouts extend filopodia to guide the growing vessels (11, 27, 28) (Supplemental Introduction). However, even though peri-/neurovascular crosstalk and metabolism are crucial features of brain tumors (4, 5, 7, 8, 29–35), less is known about how developmental signaling pathways are reactivated in brain tumors to regulate angiogenesis and endothelial metabolism (4, 8, 31, 32).

The reactivation of fetal signaling programs in tumor tissues is defined as the oncofetal axis and has been described in cancer cells in brain and peripheral tumors (18–24) as well as in ECs in liver cancer (25). However, the relevance of the oncofetal axis in endothelial and perivascular cells of the brain vasculature in human (glial) brain tumors is currently unknown. Oncofetal programs represent interesting therapeutic targets, since they are upregulated in the brain tumor tissue as compared with the surrounding healthy brain, thereby reducing the likelihood of side-effects (36). Thus, a better understanding of the oncofetal axis in the (glial) brain tumor vasculature harbors great scientific and translational potential, such as the potential identification of novel therapeutic targets.

Nucleolin (NCL) is a multifunctional and widely expressed protein found in various cell compartments of eukaryotic cells (nucleoplasm, nucleolus, cytoplasm, and plasma membrane) (37), and its main functions are the regulation of ribosome biogenesis and ribosomal RNA (rRNA) synthesis (38–40), while it also regulates cell cycle, senescence, apoptosis, and angiogenesis (37, 38, 41). NCL expression increases with malignancy grade and proliferation rate in both human and mouse glial brain tumors (42–45), indicating its proproliferative role in glioma (Supplemental Introduction). Targeting NCL in cancer cells using the anti-NCL aptamer AS1411 has been reported in brain and peripheral tumors (46–48), whereas AS1411-mediated targeting of NCL in ECs of the pathological vasculature in the retina resulted in reduced angiogenicity (49, 50). However, AS1411 has not been used to target NCL in the vasculature of tumors inside and outside the CNS. In peripheral tissues, NCL was shown to be upregulated at the cell surface of ECs in angiogenic vessels in breast tumors (51), with regulatory effects on carcinogenesis and angiogenesis, and it was shown to regulate EC motility and tube formation in vitro (52). Moreover, targeting endothelial NCL induced endothelial apoptosis and vessel normalization in a pancreatic tumor mouse model (53, 54). However, the role of NCL on angiogenesis and EC function in the developing human brain and in human gliomas remains poorly understood. Here, using a variety of in vivo and in vitro assays, we show that NCL is an oncofetal protein in human gliomas regulating sprouting angiogenesis and endothelial metabolism.

## Results

### NCL is a neurodevelopmental protein of the oncofetal axis that is silenced in the healthy adult brain and reactivated in the NVU/PVN of glial brain tumors.

**Figure 1:**
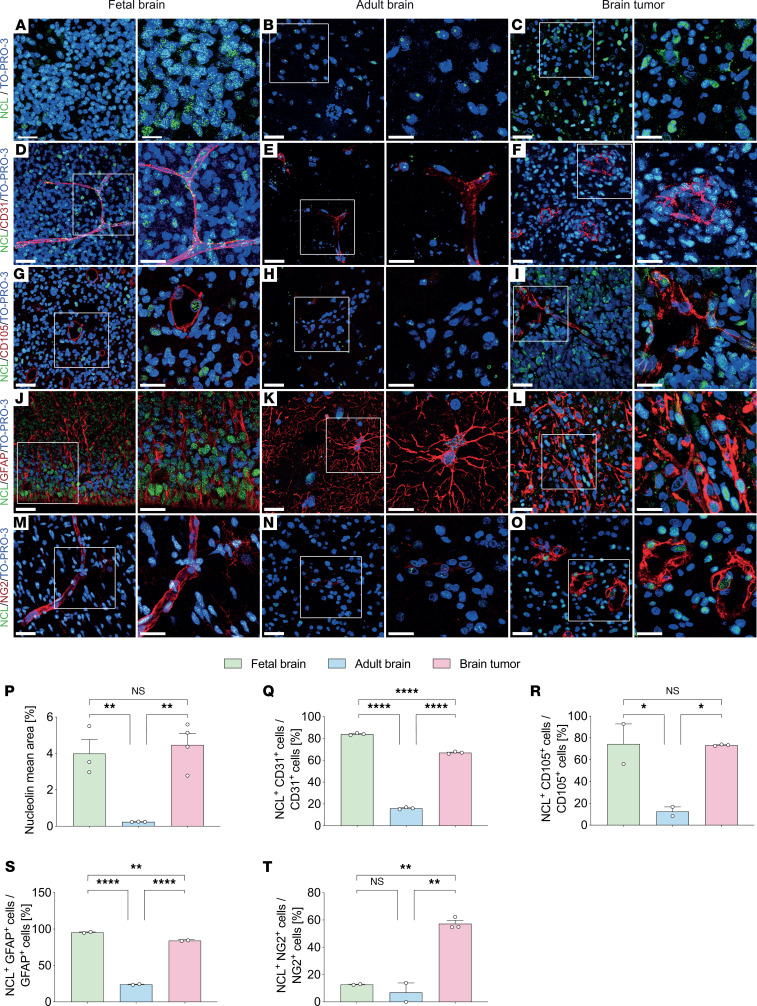
Nucleolin is expressed in endothelial and perivascular cells during human brain development, is downregulated in the adult brain, and is reactivated in glial brain tumors in vivo. Sections (20 μm) of human fetal (GW 18-22) and adult brains as well as sections from human GBMs were stained for Nucleolin, the vascular endothelial cell markers CD31 and CD105, the astrocytic marker GFAP, the pericyte marker NG2, and TO-PRO-3 nuclear counterstaining. (**A**–**C** and **P**) Nucleolin (green) is highly expressed in the nuclei of the developing human fetal brain (**A**) and of human brain tumors (**C**), but it shows a significant downregulation in the adult normal/healthy brain (**B** and **P**). (**D**–**I**, **Q**, and **R**) Nucleolin (green) is highly expressed in CD31^+^ blood vessel endothelial cells (red) in the human fetal (**D** and **Q**) and pathological brain (**F** and **Q**), but it is significantly downregulated in endothelial cell of the quiescent adult brain (**E** and **Q**). (**G**–**I** and **R**) Nucleolin shows a high expression in CD105^+^ activated endothelial cells (red) in the human fetal brain (**G** and **R**) and in glioblastoma (**I** and **R**) but is significantly downregulated in the quiescent adult normal brain (**H** and **R**), with a very low number of CD105^+^ endothelial cells in the quiescent adult brain (**H** and **R**). (**J**–**L** and **S**) Nucleolin (green) is highly expressed in GFAP^+^ neural precursors cells (red) in the fetal brain (**J**) and in tumoral astrocytes in glioblastoma (**L**), but it is significantly downregulated in adult normal brain (**K** and **S**). (**M**–**O** and **T**) In human fetal and adult brains, NG2^+^ pericytes (red) partially express low levels of Nucleolin (**M** and **N**); Nucleolin expression is highly upregulated in human brain tumor NG2^+^ pericytes (**O** and **T**). Data represent mean ± SEM of 2–3 patients (2–3 sections per patient, based on tissue availability). For statistical analysis, 1-way ANOVA with Tukey’s post hoc test were performed. *P < 0.05, **P < 0.01, ****P < 0.0001. Scale bars: 25 μm in **A**–**O**, left panels; and 15 μm in **A**–**O**, right panels.

To investigate whether NCL constitutes a neurodevelopmental protein that is reactivated in brain tumors, we performed immunofluorescence microscopy of the main NVU/PVN cellular components of human fetal brain, of human normal adult brain, and of human glial brain tumors. NCL was highly expressed during fetal forebrain neocortex development, at gestational week 18 (GW18) and GW22, significantly downregulated in the adult brain, and upregulated in brain tumors, as revealed by immunofluorescence staining against NCL and the nuclear marker TO-PRO-3 (55) (Figure 1, A–C and P). Moreover, NCL expression was present throughout the nucleoplasm during fetal development but was restricted to the nucleolus in the adult human brain (Figure 1, A and B). In GBM, NCL expression was detected across the entire nucleoplasm and appeared similar to the pattern observed during fetal development (Figure 1, A and C). Within the NVU, NCL was expressed in both endothelial and perivascular cells (Figure 1, D–O). NCL was highly expressed in cells labeled with the endothelial marker cluster of differentiation 31 (CD31) during brain development, where 84% of CD31^+^ ECs showed NCL expression across the entire nucleoplasm (Figure 1, D and Q). NCL was significantly downregulated in the adult brain, with 16% of the CD31^+^ cells being NCL^+^ ECs (predominant nucleolar expression) (Figure 1, E and Q), but it was significantly upregulated in GBM ECs, where 67% of the CD31^+^ were NCL^+^ (nucleoplasm and nucleolar expression), similar to its expression in fetal brain (Figure 1, D, F, and Q). NCL was also highly expressed in CD105^+^ angiogenic ECs during fetal brain development and in GBM, with 75% and 74% CD105^+^/NCL^+^ double-positive ECs, respectively (Figure 1, G, I, and R), whereas only 13% CD105^+^/NCL^+^ ECs could be observed in adult brain slices (Figure 1, H and R), consistent with the reported quiescence of ECs in the adult normal brain (11, 56, 57).

Within the NVU, blood vessel ECs are in contact with perivascular supportive cells such as pericytes, astrocytes, and neuronal stem cells (11, 57–61). Therefore, we assessed the expression of NCL in pericytes and perivascular astrocytes. Glial fibrillary acidic protein–positive (GFAP^+^) astrocytes formed typical patterns by contacting ECs and showed very strong NCL expression during fetal brain development with 95% of GFAP^+^/NCL^+^ astrocytes (Figure 1, J and S). In contrast, in the adult brain, NCL was significantly downregulated in GFAP^+^ astrocytes with only 24% GFAP^+^/NCL^+^ (no restriction to nucleolus observed), but it showed a significant upregulation in GBM with 84% of GFAP^+^/NCL^+^ astrocytes (Figure 1, J, K, and S). Interestingly, neuron-glial antigen 2–positive (NG2^+^) pericytes showed low NCL expression in fetal brain development with only 13% NG2^+^/NCL^+^ pericytes as well as in the adult brain with 7% NG2^+^/NCL^+^ pericytes (Figure 1, M, N, and T). However, NCL was significantly upregulated in human GBM with 57% NG2^+^/NCL^+^ pericytes (Figure 1, O and T).

Taken together, these data reveal that NCL is highly expressed in endothelial and certain NVU cells (astrocytes > pericytes) during fetal brain development, is subsequently downregulated in the adult brain, and is reactivated in GBM. This characterizes NCL as an oncofetal protein that is reactivated in human glial brain tumors after downregulation in the quiescent adult NVU (18–25).

### NCL expression within the NVU correlates with glial brain tumor malignancy and progression.

**Figure 2:**
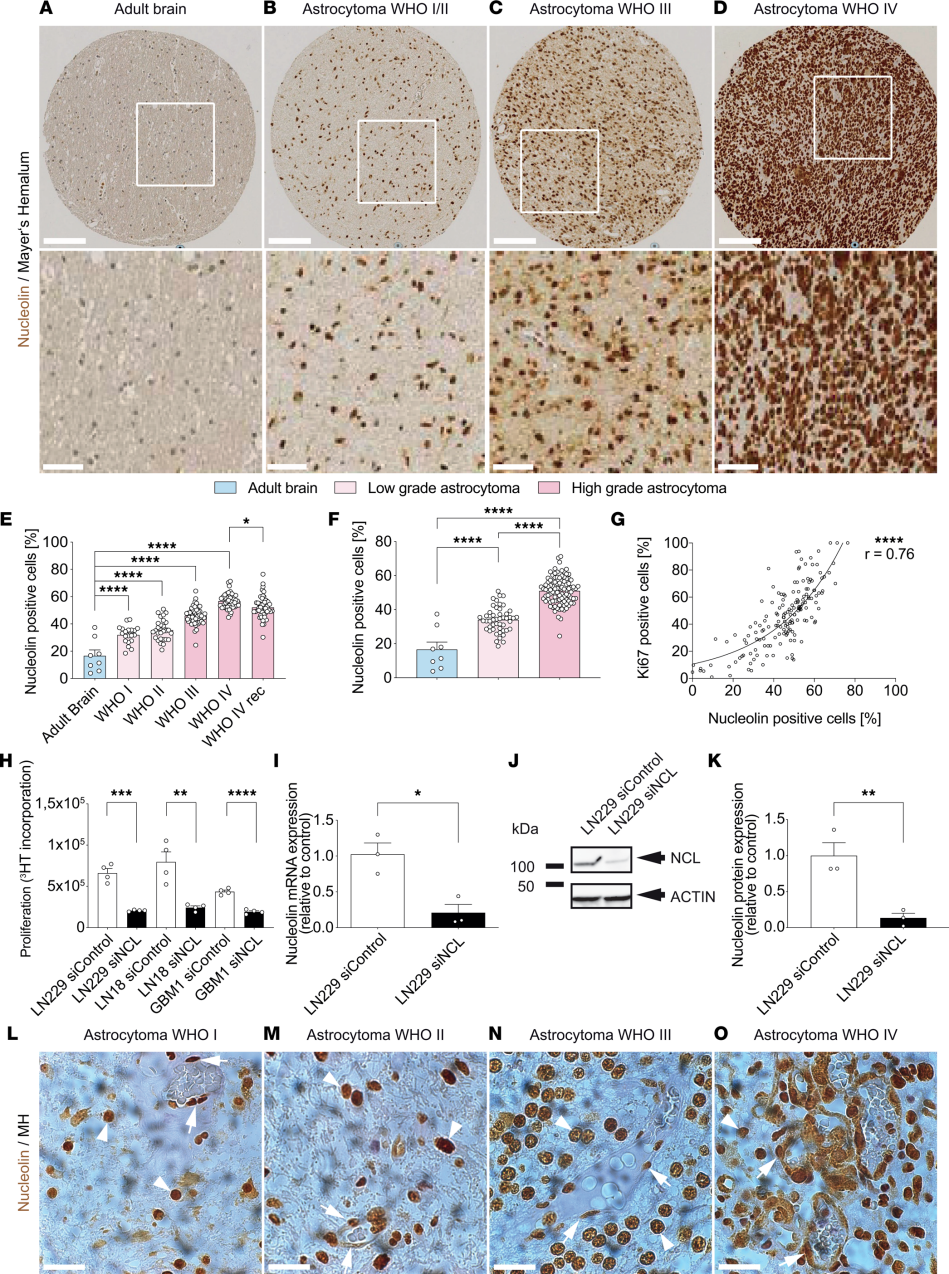
Expression of NCL increases during astrocytic tumor progression and NCL is expressed in blood vessels in low- and high-grade gliomas in vivo. (**A**–**F**) NCL (brown) expression increases during tumor progression of human astrocytomas from WHO grade I (**A**, *n* = 20), II (**B**, *n* = 28), III (**C**, *n* = 53), to IV (= glioblastoma, **D**, *n* = 46). In low-grade astrocytomas (WHO grades I and II, *n* = 48), NCL expression is significantly higher than in the normal brain parenchyma (**E** and **F**, *n* = 8) but significantly lower as compared with high-grade astrocytomas (**E** and **F**, WHO grades III and IV, *n* = 96). NCL expression slightly decreases in recurrent WHO grade IV tumors (**E**, *n* = 49). (**G**) NCL expression positively correlates with the expression of the proliferation marker Ki-67 in gliomas (*n* = 167). (**H**) Glioblastoma cell lines (LN-229, LN-18, and GBM-1) proliferation was significantly decreased upon NCL knockdown *n* = 4). (**I**) qPCR revealing a significant downregulation of about 70% of NCL mRNA expression upon siRNA-targeted NCL knockdown (siNCL, *n* = 3). (**J**) Western blot showing NCL downregulation in LN-229 cells transfected with siRNA against NCL (siNCL) (*n* = 3). No NCL downregulation was observed in LN-229 transfected with the control siRNA (siControl) (*n* = 3). (**K**) Quantification of Western blot revealing a significant downregulation of NCL protein expression by siRNA-targeted NCL knockdown as compared with control cells in LN-229 cells (*n* = 3). (**L**–**O**) NCL expression in tumor blood vessels. Note the increasing expression of NCL in the blood vessel wall (arrows) as well as in perivascular cells (arrowheads) in astrocytomas of higher grades. Data represent mean ± SEM. For statistical analysis, 1-way ANOVA with Tukey’s post hoc test (**E** and **F**), Pearson correlation analysis (**G**), and 2-tailed unpaired Student’s *t* test (**H**, **I**, and **K**) were performed. *P < 0.05, **P < 0.01, ****P < 0.0001. Scale bars: 100 μm (**A**–**D**, upper panel), 50 μm (**A**–**D**, lower panel, and **G**–**J**).

In order to address the expression of NCL in glial brain tumor progression (from WHO low- to high-grade tumors; refs. 62–64), we referred to tissue microarrays (TMAs) of human glioma stained by IHC for NCL and the nuclear marker Mayer’s hemalum (Figure 2, A–D). NCL expression was markedly upregulated in human glial brain tumors as compared with the adult normal brain (Figure 2, A–E). Moreover, NCL expression was significantly increased during glial tumor progression, ranging from 32% of NCL^+^ cells in WHO grade I glioma to 57% in glioma grade IV (GBM) (Figure 2E). NCL showed a significant upregulation from low-grade (WHO grade I and II) to high-grade glioma (WHO grade III) as well as a significant increase from WHO grade III to WHO grade IV glioma (Figure 2, A–D, and F). In recurrent GBM, NCL expression showed a slight but significant decrease as compared with primary GBM (Figure 2E). NCL expression correlated well with the established proliferation marker Ki-67 (65) in all glioma grades (Figure 2G).

Based on NCL expression within perivascular cells of the developmental and tumoral NVU, we next addressed its effects on tumoral cell proliferation. To determine whether NCL promotes GBM cell proliferation, NCL was knocked down in the human GBM cell lines LN-229 (66) and LN-18 (67) as well as in freshly isolated primary human GBM cells (GBM-1) using siRNA (Figure 2, I–K). Cell proliferation of LN-229, LN-18, and GBM-1 was inhibited by siRNA-mediated knockdown of NCL (Figure 2H) compared with scrambled controls, in agreement with the previously reported strong proproliferative effect of NCL in human GBM cells (42, 68).

Next, we examined the expression of NCL in tumor blood vessels using spatial transcriptomics and IHC. In agreement with our immunofluorescent data in GBMs (Figure 1, F and Q), exploratory spatial transcriptomics in human GBMs showed cooccurrence of NCL and various endothelial markers including CD31 (*PECAM1*), CD105 (*ENG*), *CLDN5*, and *VWF* (Supplemental Figure 1, A–L). Moreover, NCL was indeed present in the wall of tumor blood vessels, showing an increased expression during glial tumor progression (WHO I–IV; Figure 2, L–O), further suggesting a regulatory effect on human glial brain tumor angiogenesis.

Taken together, these data indicate that NCL expression is reactivated in tumor cells and tumor ECs within the NVU during human astrocytic tumor progression.

### NCL is expressed within the NVU and in sprouting ETCs, and it promotes the number of tip cell filopodia during brain development in vivo.

**Figure 3:**
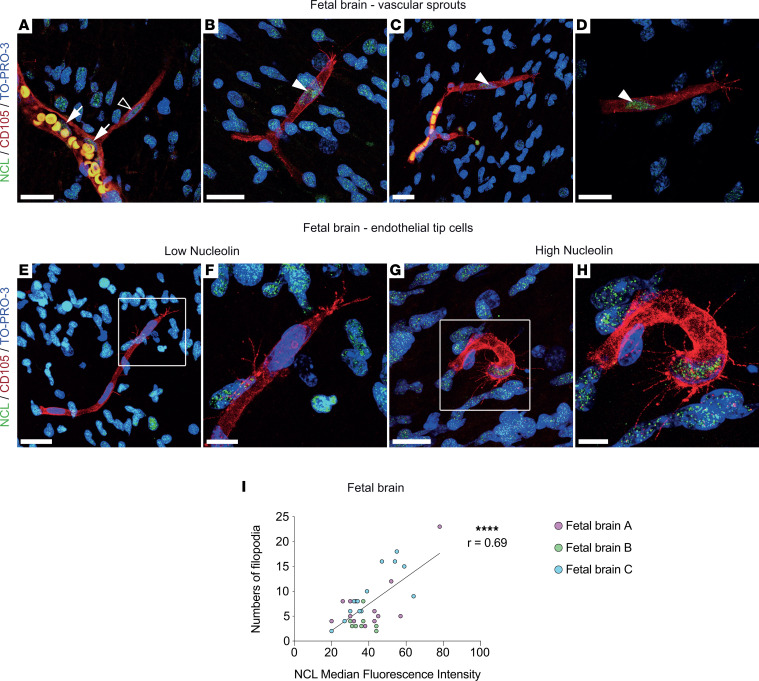
NCL is expressed in endothelial tip, stalk, and phalanx cells of vascular sprouts, and NCL expression correlates with the number of endothelial tip cell filopodia in the human fetal brain in vivo. (**A**–**D**) CD105^+^ blood vessel sprouts (red) in the human fetal brain grow in CNS tissue with vascular and parenchymal expression of NCL. NCL (green) is expressed in endothelial tip (filled arrowheads in **B**–**D**) stalk (empty arrowhead in **A**) and phalanx cells (empty arrows in **A**) in growing vessels in the human fetal (GW18-22) cortex. (**E**–**H**) Vascular sprouts with CD105-labeled endothelial tip cells (red) with low (**E** and **F**) and high (**G** and **H**) NCL (green) expression in the human fetal cortex. Numerous filopodial protrusions emerged from the endothelial tip cell body with high NCL expression (**G** and **H**) as compared with only few filopodial protrusions in an endothelial tip cell with low NCL expression (**E** and **F**). (**I**) The number of filopodia per endothelial tip cell strongly correlated with the intensity of NCL expression in the respective endothelial tip cell. Each dot represents an endothelial tip cell with its median NCL expression and number of filopodia (*n* = 35). Dots are colored by patients (*n* = 3 patients, 2–3 sections per patient, based on tissue availability). Pearson correlation analysis was performed. ****P < 0.0001. Scale bars: 20 μm in **A**–**D**; 25 μm in **E** and **G**; and 10 μm in **F** and **H**.

NCL has been shown to affect tumor and blood vessel growth in peripheral tissues (42, 51, 52, 68, 69), but whether it regulates sprouting angiogenesis during brain development remains unknown. To assess whether NCL affects sprouting angiogenesis and ETCs during brain development, we addressed NCL expression in the vicinity of sprouting blood vessels in the human fetal brain. CD105-labeled ETCs with their typical, finger-like protruding filopodia could be recognized in GW18 and GW22 human fetal brain forebrains (Figure 3, A–D). NCL was expressed in CD105^+^ endothelial tip, stalk, and phalanx cells (Figure 3, A–D) as well as in perivascular cells surrounding sprouting capillary ETCs (filopodia) (Figure 3, A–D). We observed NCL in nuclei of CD105^+^ endothelial tip, stalk, and phalanx cells but not on the (endothelial and perivascular) cell surface or in filopodia protrusions (Figure 3, A–D).

We examined whether NCL expression affected the number of ETC filopodia and observed a lower number of filopodia in ETCs with low NCL expression (Figure 3, E and F) and a higher number of filopodia in ETCs with high NCL expression (Figure 3, G and H). Accordingly, we quantified the number of filopodia per NCL^+^/CD105^+^ ETCs and assessed NCL expression for each ETC. Indeed, the number of filopodia positively correlated with NCL expression in the ETCs, as revealed by median fluorescence intensity (Figure 3I). These results strongly suggest that NCL positively regulates the number of ETC filopodia in the human fetal brain.

### NCL promotes HCMEC and HUVEC sprouting angiogenesis in vitro.

**Figure 4:**
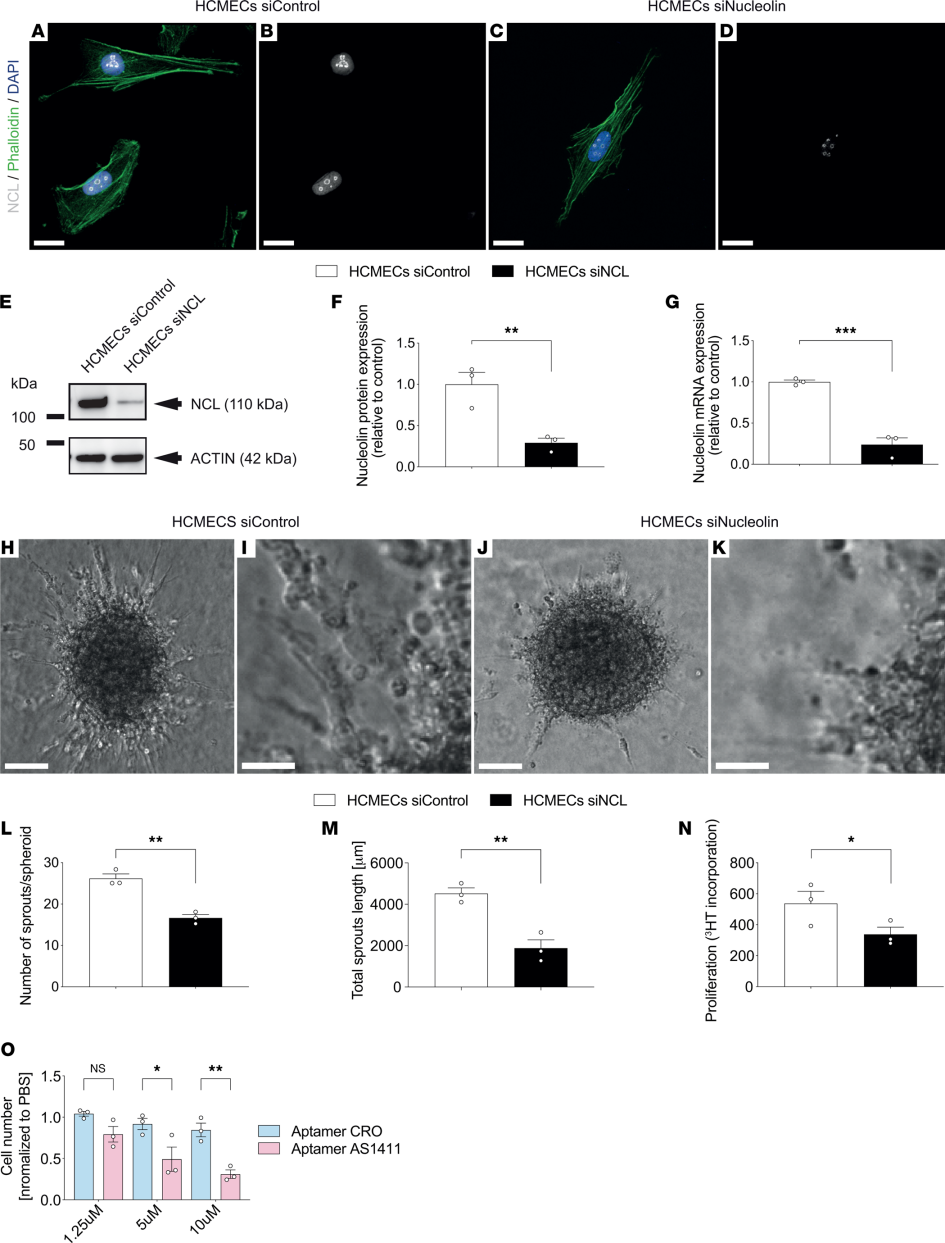
NCL promotes brain vascular endothelial cell sprouting and proliferation in vitro and can be targeted with the NCL-specific aptamer AS1411. (**A**–**D**) HCMECs were stained for NCL (green), F-actin (stained with Phalloidin, red), and the general nuclear marker DAPI (blue). NCL expression was decreased and restricted to nucleoli upon siRNA-mediated knockdown in HCMECs (**C** and **D**) as compared with control-siRNA treated HCMECs (**A** and **B**). (**E**) Western blot showing NCL downregulation in HCMECs transfected with siRNA against NCL (siNCL) as compared with control siRNA–treated HCMECs (*n* = 3). (**F**) Quantification of Western blot revealing a significant downregulation of 70% NCL protein expression by siRNA-targeted NCL knockdown as compared with control cells (*n* = 3). (**G**) qPCR revealing a significant downregulation of more than 80% of NCL mRNA expression by siRNA-targeted NCL knockdown (*n* = 3). (**H**–**K**) HCMEC sprout formation (number of sprouts per spheroid) was decreased upon siRNA-mediated NCL knockdown (**J** and **K**) as compared with the control group (**H** and **I**). The boxed areas in **H** and **J** are enlarged in **I** and **K,** respectively. (**L** and **M**) HCMEC sprout formation and total sprout length were significantly reduced upon NCL knockdown as compared with the control group (**L** and **M**) (*n* = 3). (**N**) HCMEC proliferation was significantly decreased upon NCL knockdown (*n* = 3). (**O**) HCMEC cell proliferation was dose-dependently decreased upon treatment with the NCL-specific aptamer AS1411. Cells were treated for 96 hours with 1.25 μM, 5 μM, and 10 μM of the NCL targeting aptamer AS1411 or the control aptamer CRO (*n* = 3). Data represent mean ± SEM. For statistical analysis, 2-tailed unpaired Student’s *t* test (**F**–**G** and **L**–**N**) and 2-way ANOVA Tukey’s multiple-comparison test comparing treatment columns (**O**) were performed. *P < 0.05, **P < 0.01, ***P < 0.001, ****P < 0.0001. Scale bars: 20 μm in **A**–**D**; 150 μm in **H** and **J**; and 50 μm in **I** and **K**.

Based on these expression studies suggesting a role for NCL in sprouting angiogenesis and ETC filopodia in vivo, we next investigated the functional role of NCL in human angiogenic EC sprouting in vitro. We used siRNA to knock down NCL in human cerebral microvascular EC/D3 (HCMEC/D3, hereafter referred to as HCMEC) and HUVECs (Figure 4, A–G, and Supplemental Figure 2, A–G). In NCL siRNA-treated HCMECs (HCMEC^NCL^ ^KD^) and HUVECs (HUVEC^NCL^ ^KD^), NCL expression was decreased and confined to the nucleolus when compared with the siRNA control–treated HCMECs (HCMEC^Control^ ^KD^) and HUVECs (HUVEC^Control^ ^KD^) (Figure 4, A–D, and Supplemental Figure 2, A–D). Accordingly, quantitative PCR (qPCR) and Western blot analysis revealed significant knockdown of NCL at both the mRNA and the protein levels in HCMECs and HUVECs (Figure 4, E–G, and Supplemental Figure 2, E–G).

To test the effects of NCL on sprouting angiogenesis in vitro, we referred to an in vitro spheroid angiogenesis assay (70). HCMECs and HUVECs in the sprouting spheroid assay grew vessel-like sprouts composed of multiple branches in the control group (Figure 4, H and I, and Supplemental Figure 2, H and I). In contrast, siRNA-mediated knockdown of NCL markedly suppressed the number of vessel sprouts per spheroid as well as the length of the sprouts as compared with the control group in both cell types (Figure 4, J–M, and Supplemental Figure 2, J–M), suggesting a promoting effect on sprouting angiogenesis.

Given the important role of NCL in cell proliferation (37, 39, 40), we next assessed whether NCL affects EC proliferation in a ^3^HT proliferation assay. Indeed, HCMEC and HUVEC proliferation was significantly reduced upon siRNA-mediated NCL knockdown (Figure 4N and Supplemental Figure 2N), indicating a positive regulatory role for NCL on HCMEC and HUVEC proliferation, reminiscent of stalk cell behavior in vivo (11, 27, 57).

Because we observed NCL expression in brain ECs (Figure 1, D–F, and Figure 4, A–D), based on its positive effects on (brain) EC proliferation (Figure 4N and Supplemental Figure 2N), and given that NCL has been targeted in cancer cells and retinal ECs using the aptamer AS1411(45–50, 71, 72), we next tested the effects of the NCL-specific aptamer AS1411 on brain EC proliferation. Treatment of HCMECs using 1.25, 5, and 10 μM of AS1411 dose-dependently inhibited their proliferation after 96 hours (Figure 4O). Taken together, these results suggest that endothelial NCL is a positive regulator of sprouting angiogenesis, endothelial proliferation, and filopodia formation in the brain that can potentially be targeted in brain ECs using aptamers.

### NCL regulates HCMEC and HUVEC lamellipodia and filopodia formation and actin cytoskeleton orientation.

**Figure 5:**
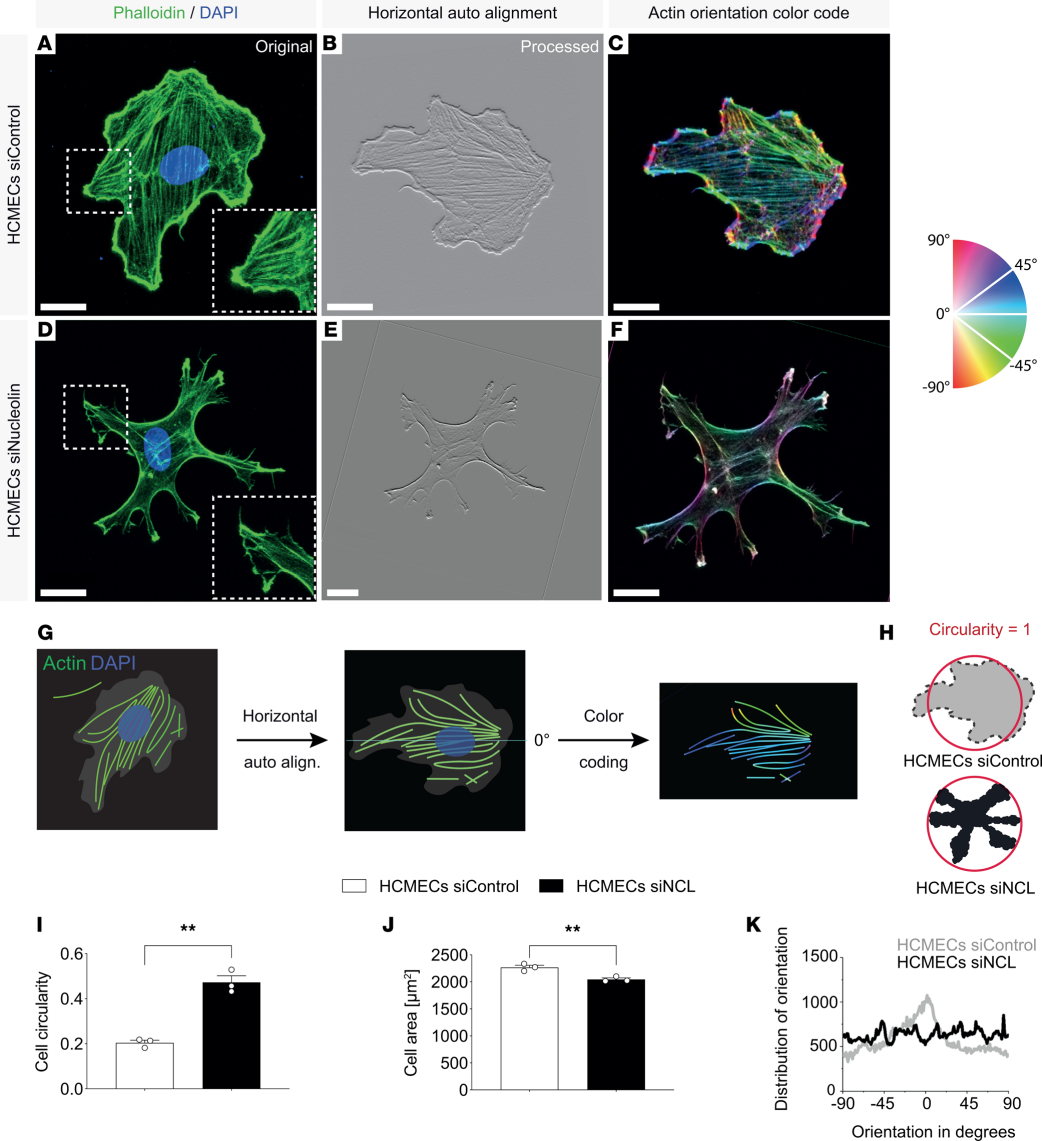
NCL affects HCMEC actin cytoskeleton orientation in vitro. (**A**–**F**) HCMEC treated with control or NCL siRNA were left to spread on fibronectin-coated glass substrate and stained for F-actin (green) and DAPI (blue), seen in **A** and **D**. (**G**) Schematic illustration of actin fiber orientation characterization. (**H**) Schematic illustration of circularity index indicating the reference circular index (circle = 1). (**I** and **J**) NCL knockdown decreased HCMEC cell spreading, as measured by cell circularity and cell area measurements. NCL knockdown HCMECs had a significantly less elongated shape (**I**, *n* = 3). HCMEC spreading was significantly decreased upon NCL knockdown (**J**, *n* = 3). (**K**) Phalloidin actin fibers (green) were more randomly organized in the HCMEC^NCL^ ^KD^ (**E** and **F**) as compared with the control (**B** and **C**). The distribution of actin orientation shows a clear classical peak close to 0 degrees in the control HCMEC^Control^ ^KD^ (gray curve). In HCMEC^NCL^ ^KD^, the classical peak of actin orientation was lost and HCMEC actin orientation was more randomly distributed (black curve) (**K**). Data represent mean ± SEM. Two-tailed unpaired Student’s *t* test were performed. **P < 0.01. Scale bars: 20 μm in **A**–**F**.

Lamellipodia and filopodia are composed of actin and myosin fibers, and they are essential components of in vivo sprouting angiogenesis (28). Therefore, to further assess the effects of NCL on HCMEC and HUVEC angiogenesis in vitro, we addressed cell shape and morphology as well as actin orientation after NCL knockdown (Figure 5, A–K, and Supplemental Figure 3, A–K). HCMEC^NCL^ ^KD^ and HUVEC^NCL^ ^KD^ spread less and were not as elongated as control cells, as quantified by their increased cell circularity and decreased cell area (Figure 5, I and J, and Supplemental Figure 3, I and J). F-actin fibers were more randomly organized in the HCMEC^NCL^ ^KD^ and HUVEC^NCL^ ^KD^ (Figure 5, C and F, and Supplemental Figure 3, C and F). Accordingly, the distribution of actin orientation showed a classical peak in controls, whereas in the HCMEC^NCL^ ^KD^ and HUVEC^NCL^ ^KD^, the actin orientation was more randomly distributed (Figure 5K and Supplemental Figure 3K), indicating the HCMEC^NCL^ ^KD^ and HUVEC^NCL^ ^KD^ had poorly orientated stress fibers, as opposed to well-aligned stress fibers of control cells.

**Figure 6:**
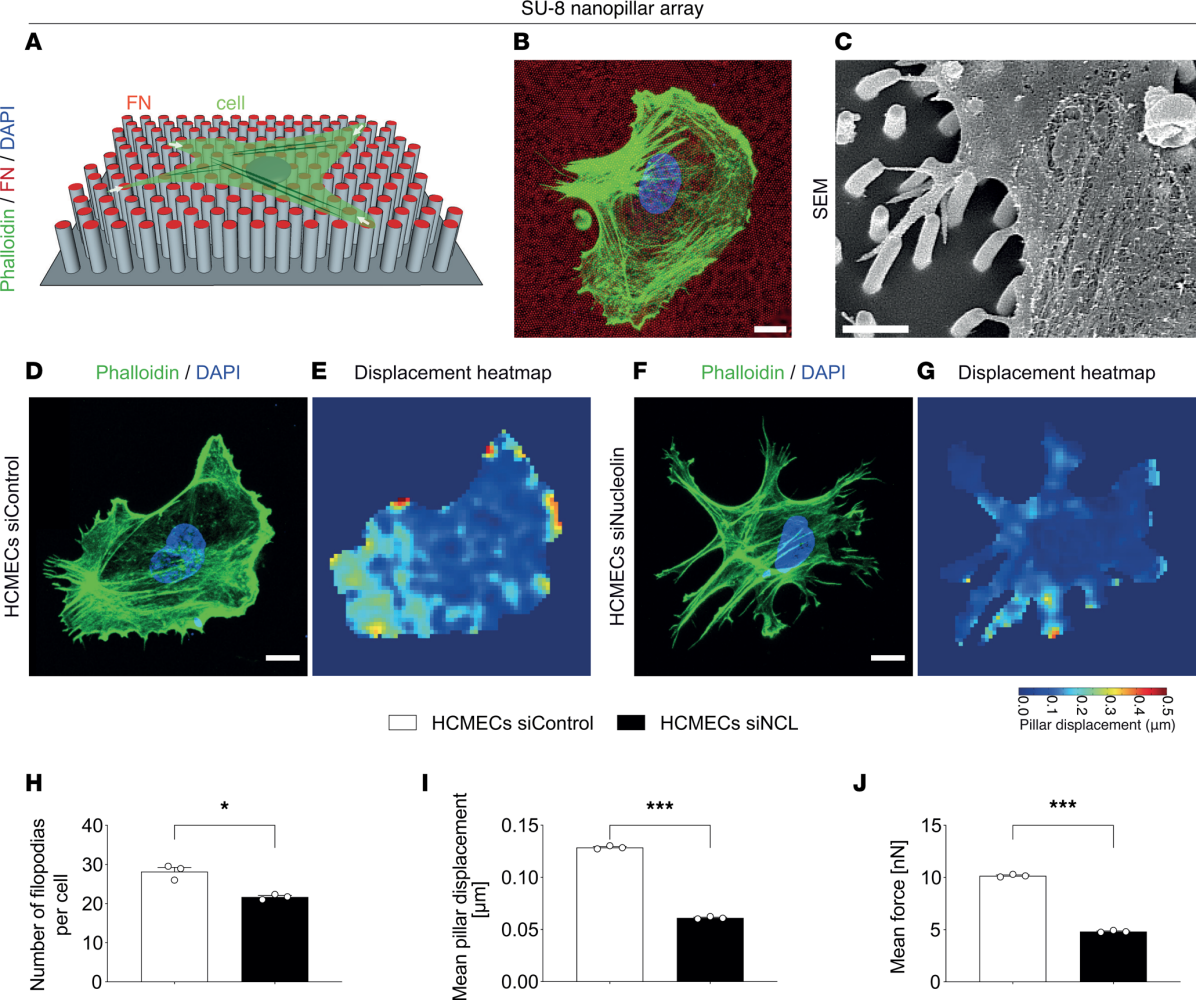
NCL regulates HCMEC lamellipodia and filopodia in vitro. (**A**) Scheme illustrating a spread endothelial cell (green) on a SU-8 nanopillar array (gray) coated with fibronectin (red). (**B**) F-actin (green) and DAPI (blue) stained HCMEC on a fibronectin-coated nanopillar substrate (red). (**C**) Scanning electron microscopy image of HCMEC filopodia attaching to nanopillars. Note the nanopillar-deflection caused by retracting HCMEC filopodia (arrowheads), allowing to optically measure the displacement of the nanopillar and the induced corresponding traction forces. (**D**–**J**) HCMECs treated with siRNA (for NCL, and control) were placed on nanopillar substrates and were stained for F-actin (green) and the general nuclear marker DAPI (blue) (**D** and **F**). The number of filopodia per cell was decreased significantly in siRNA-mediated NCL knockdown in comparison with control siRNA-treated HCMECs (**H**, *n* = 3). Explorative movements of HCMECs (and its lamellipodia and filopodia extensions) were reduced upon NCL knockdown, as evidenced by displacement heatmaps (**E** and **G**, *n* = 3). NCL downregulation decreased mean nanopillar displacement (**I**) and mean filopodia force (**J**) accordingly. Data represent mean ± SEM. Two-tailed unpaired Student’s *t* tests were performed. *P < 0.05, ***P < 0.001. Scale bars: 10 μm in **B**; 2 μm in **C**; and 5 μm in **D**–**G**.

To assess the effects of NCL on HCMECs and HUVECs and their filopodia, we cultured these cells on a substrate consisting of nanopillar arrays (73, 74) (Figure 6, A–C, and Supplemental Figure 4, A–C), allowing assessment of filopodia dynamics and traction/pulling forces exerted by the spreading of HCMECs and HUVECs on the substrate (Figure 6, D–G, and Supplemental Figure 4, D–G). In HCMEC^NCL^ ^KD^ and HUVEC^NCL^ ^KD^, the number of filopodial extensions per cell was significantly reduced as compared with HCMEC^Control^ ^KD^ and HUVEC^Control^ ^KD^ (Figure 6, D, F, and H, and Supplemental Figure 4, D, F, and H).

Next, we examined the movement of NCL siRNA–treated HCMECs and HUVECs on the nanopillar surface. In HCMEC^NCL^ ^KD^ and HUVEC^NCL^ ^KD^, the mean displacements of the nanopillars were significantly decreased as compared with the control group (0.061 μm and 0.129 μm, and 0.068 μm and 0.136 μm, respectively; Figure 6, E, G, and I, and Supplemental Figure 4, E, G, and I). HCMEC^NCL^ ^KD^ and HUVEC^NCL^ ^KD^ exerted significantly reduced average traction forces of 4.8 nN and 5.4 nN when compared with the control HCMECs and HUVECs (10.1 nN and 10.7 nN, respectively; Figure 6J and Supplemental Figure 4J). These results indicate a proadhesive/promigratory/proexplorative effect of NCL on ECs and their filopodial protrusions.

These data indicate that NCL is important for actin orientation and polarization, which is required for EC lamellipodia and filopodia formation, structures that are crucial for migration, proliferation, and sprouting of vascular ECs in vivo.

### Bulk RNA-Seq reveals regulation of angiogenic pathways including VEGF-A–VEGFR2 as well as of endothelial metabolism upon NCL knockdown.

**Figure 7:**
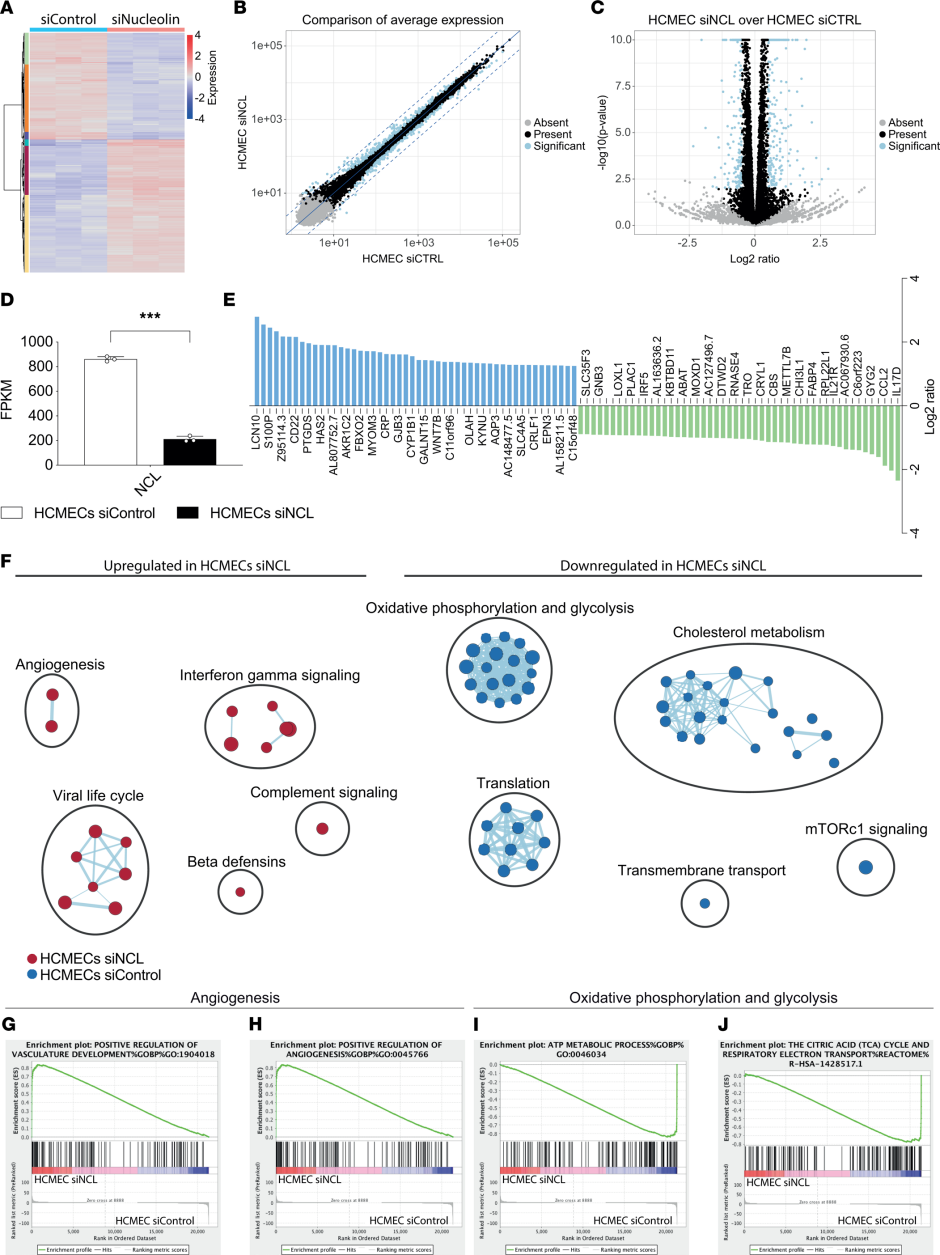
NCL induces regulation of angiogenic pathways including Dll4-Jagged-Notch-Hey-Hes, YAP-TAZ-CTGF-Cyr61, VEGF-A–VEGFR2, and endothelial glucose metabolism in HCMECs. Transcriptome analysis via RNA-Seq of HCMECs treated with siRNA against NCL (siNCL) and control siRNA (siControl) in 3 independent experiments. (**A**) Heatmap and hierarchical clustering of siNCL-treated HCMECs as compared with siControl-treated HCMECs. (**B** and **C**) A total of 445 genes was differentially regulated between HCMECs^NCL^ ^KD^ and HCMECs^Control^ ^KD^, indicated in blue on scatter (**B**) and volcano plots (**C**). (**D**) NCL gene expression (FPKM) was significantly downregulated by siNCL treatment. (**E**) Top 50 significantly upregulated (blue) or downregulated (green) genes detected by RNA-Seq in HCMECs upon NCL knockdown as compared with control treatment. Differentially regulated genes were arranged according to fold change of gene expression. (**F**–**J**) GSEA, cytoscape enrichment map (**F**), and enrichment plots showed a significant upregulation of signaling pathways related to regulation of angiogenesis (**F**–**H**) in HCMECs treated with siRNA against NCL, whereas pathways involved in metabolic processes such as oxidative phosphorylation and glycolysis (**F**, **I**, and **J**) and cholesterol metabolism were enriched in the control treatment. Pathways enriched in NCL-knockdown HCMECs are labeled in red, and pathways enriched in control HCMECs are labeled in blue. Pathways are indicated by colored nodes. Their size represents the number of genes they contain. Green lines indicate relationships between the pathways. Black circles group related pathways. Data represent mean ± SEM. Wald tests corrected for multiple testing using the Benjamini-Hochberg method were performed. ***P < 0.001.

To address the downstream signaling pathways induced by NCL in human ECs, we performed an unbiased transcriptome analysis by RNA-Seq of HCMEC^NCL^ ^KD^ and HCMEC^Control^ ^KD^ as well as of HUVEC^NCL^ ^KD^ and HUVEC^Control^ ^KD^. RNA-Seq revealed 445 and 3,240 significantly differentially regulated genes between HCMEC^NCL^ ^KD^ and HCMEC^Control^ ^KD^ and between HUVECs^NCL^ ^KD^ and HUVECs^Control^ ^KD^, respectively (Figure 7, A–C, and Supplemental Figure 5, A–C). Analysis of the gene expression by the fragments per kilobase of exon model per million reads mapped (FPKM) values confirmed NCL downregulation mediated by siRNA in HCMECs and HUVECs (Figure 7D and Supplemental Figure 5D). In HCMECs^NCL^ ^KD^, genes involved in inflammatory responses including *IL17D* and *CCL2* (75) were among the top-regulated genes (Figure 7E and Supplemental Results).

Next, to address the molecular pathways regulated by NCL in ECs, we performed gene set enrichment analyses (GSEA) (76) between siNCL-treated HCMECs/HUVECs and the control HCMECs/HUVECs. In HCMEC^NCL^ ^KD^, GSEA followed by pathway visualization using cytoscape (77) revealed that upregulated genes were mainly involved in regulation of angiogenesis and immune-related processes, while the downregulated genes were linked to regulation of metabolic processes and translation (Figure 7F). Indeed, angiogenesis and vascular development were significantly enriched upon NCL depletion in HCMECs, whereas oxidative phosphorylation and glycolysis showed significant enrichment in control HCMECs (Figure 7, F–J).

**Figure 8:**
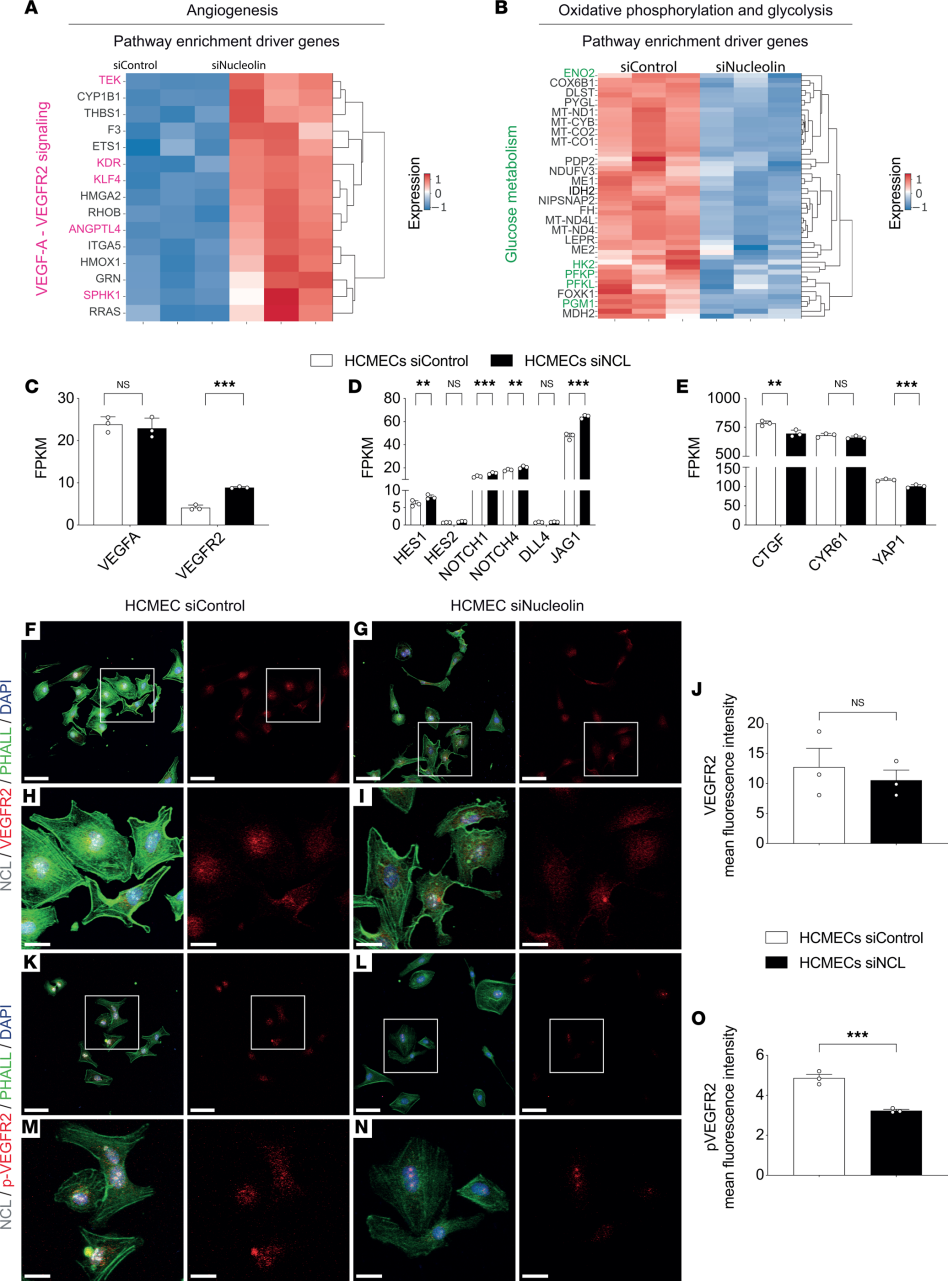
NCL induces regulation of angiogenic pathways and promotes phosphorylation of VEGFR2 in HCMECs. (**A** and **B**) Heatmap showing the expression of the top 15 genes driving the enrichment of “angiogenesis” pathways in HCMEC^NCL^ ^KD^ and the top 50 genes driving the enrichment of “oxidative phosphorylation and glycolysis” pathways in HCMEC^Control^ ^KD^. (**C**–**E**) *VEGFA* expression was not differentially regulated, and *VEGFR2* expression was upregulated (**C**) in HCMECs^NCL^ ^KD^ as compared with HCMECs^Control^ ^KD^. NCL knockdown induced a significant upregulation of the Dll4-Jagged-Notch signaling pathway, including in *HES1*, *NOTCH1*, *NOTCH4*, and Jagged1 (*JAG1*), while HES2 and DLL4 were not differentially regulated upon NCL knockdown (**D**). siNCL treatment caused a significant downregulation of the YAP-TAZ gene *YAP1* as well as the YAP-TAZ downstream effector gene *CTGF* but not of the YAP-TAZ downstream effector gene *CYR61* (**E**). (**F**–**O**) HCMECs were stained for NCL (gray), F-actin (green, stained with phalloidin), VEGFR2 (red, in **F**–**I**), or p-VEGFR2 (red, in **K**–**N**) and the general nuclear marker DAPI (blue). VEGFR2 expression was not regulated (**J**, *n* = 3), but pVEGFR2 expression was significantly downregulated (**O**, *n* = 3) upon siRNA-mediated NCL knockdown in HCMECs as compared with the control condition. Data represent mean ± SEM. Wald test corrected for multiple testing using the Benjamini-Hochberg method (**C**–**E**) and 2-tailed unpaired Student’s *t* test (**J** and **O**) were performed. **P < 0.01, ***P < 0.001. The boxed areas (white box) in **F**, **G**, **K**, and **L** are zoomed-in images shown in **H**, **I**, **M**, and **N**, respectively. Scale bars: 70 μm in **F**, **G**, **K**, and **L**; 20 μm in **H**, **I**, **M**, and **N**.

We next examined genes driving the enrichment of regulation of angiogenesis and glycolysis as well as oxidative phosphorylation in brain ECs. Genes involved in the key angiogenic *VEGFA*-*VEGFR2* pathway including *VEGFR2* as well as the *VEGFA-VEGFR2* regulatory genes *KLF4* (78), *ANGPTL4* (79), *TEK* (80), and *SPHK1* (81) were significantly upregulated, whereas genes involved in the regulation of glucose metabolism such as *HK2* (82), *ALDOC* (83), *ENO2* (84), *PFKL* (85), and *PFKP* (86) were significantly downregulated in HCMEC^NCL^ ^KD^ (Figure 8, A and B), indicating that NCL regulates those genes involved in angiogenic sprouting in brain ECs. Importantly, we observed overlapping genes regulating angiogenesis (*VEGFR2*, *KLF4*, *ANGPTL4*, *TIE2*, and *SPHK1*) and metabolic processes (*HK2*, *ENO2*, and *PFKL*) in the 2 cell types following NCL depletion (Supplemental Figure 6, A and B), suggesting common underlying mechanisms driving vascular growth and endothelial metabolism in HCMECs and HUVECs (Supplemental Results).

Based on the observed regulatory effect of NCL on sprouting angiogenesis, we next analyzed the expression of main regulators of the key angiogenic pathways VEGF-A–VEGFR2/VEGFR3–Dll4–Jagged–Notch and Hippo-YAP-TAZ. We observed regulation of the central VEGF-A–VEGFR2 pathway, upregulation of the antiangiogenic Dll4-Notch pathway as well as downregulation of the proangiogenic Hippo-YAP-TAZ pathway upon NCL knockdown (Figure 8, C–E; Supplemental Figure 6, C–E; and Supplemental Results), supporting the proangiogenic role of NCL in brain and peripheral ECs.

Finally, given its central role in angiogenesis and vascular growth and based on its regulation in the bulk RNA-Seq data, we validated the alteration of transcriptional expression of *VEGFR2* at the protein level using immunofluorescence in both cell types. We observed decreased expression of the phosphorylated form of VEGFR2 (p-VEGFR2) in HCMEC^NCL^ ^KD^ and HUVEC^NCL^ ^KD^ (Figure 8, F–O, and Supplemental Figure 6, F–O), thereby indicating a positive regulatory effect of NCL on VEGFR2 and suggesting a potential crosstalk between the VEGF-A–VEGFR2 and NCL pathways in brain and peripheral ECs. Taken together, these results suggest that NCL’s positive regulatory effects on CNS sprouting angiogenesis and endothelial metabolism might be regulated by interaction with the *VEGFA-VEGFR2* pathway and with glycolytic enzymes including *HK2*.

### Metabolomics confirm regulation of endothelial glucose metabolism upon NCL knockdown.

**Figure 9:**
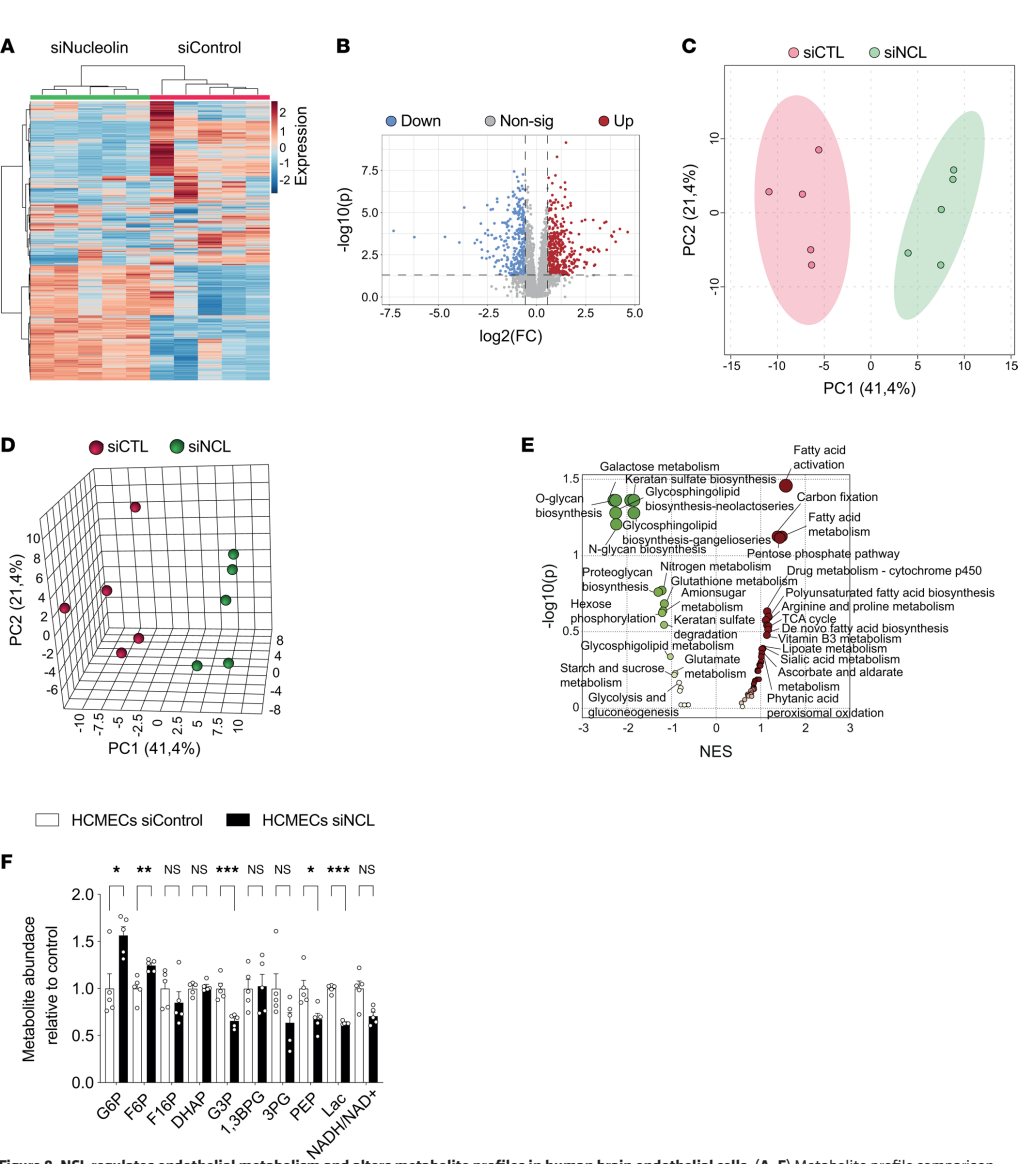
NCL regulates endothelial metabolism and alters metabolite profiles in human brain endothelial cells. (**A**–**F**) Metabolite profile comparison between siRNA NCL-knockdown HCMECs and control knockdown HCMECs (*n* = 5). Heatmap and hierarchical clustering showing 2,000 differentially regulated metabolites (**A**). Scatter plot showing the significantly regulated metabolites upon NCL KD. In total, 455 metabolites were significantly upregulated in HCMECs^NCL^ ^KD^ (indicated in red) and 292 metabolites were significantly downregulated (indicated in blue) (**B**). Two-dimensional and 3-dimensional PCA plot of log-transformed normalized concentration of 2,000 metabolites; each represent a sample and are colored by experimental group (**C** and **D**). Gene set enrichment analysis (GSEA) indicated a significant regulation of various metabolic pathway in HCMECs upon Nucleolin knockdown (**E**). Relative abundance of glycolysis intermediates showed an upregulation of both Glucose-6-Phoshpate (G6P) and Fructose-6-Phosphate (F6P), and a significant downregulation of Glyceraldehyde 3-phosphate (G3P), Phosphoenolpyruvate (PEP), and lactate (Lac) (**F**, *n* = 5). Levels of Fructose 1,6-bisphosphate (F16P), Dihydroxyacetone phosphate (DHAP), and 3-phosphoglycerate (3PG) and the ratio of NADH/NAD^+^ were not significantly regulated (**F**, *n* = 5). Data represent mean ± SEM. Two-tailed unpaired Student’s *t* test were performed. *P < 0.05, **P < 0.01, ***P < 0.001.

Endothelial metabolism is a crucial regulator of sprouting angiogenesis, ETC formation, and endothelial lamellipodia and filopodia dynamics (87–90). Moreover, EC glycolysis regulates the rearrangement of ECs by promoting filopodia formation and by reducing intercellular adhesion (90). Based on the regulation of metabolic pathways upon NCL knockdown in our bulk RNA-Seq data as well as on the observed regulatory effects of NCL on sprouting angiogenesis, ETC (filopodia), and the actin cytoskeleton, we next investigated whether NCL affected endothelial glucose and fatty acid metabolism (89). Therefore, we performed unbiased metabolic profiling using liquid chromatography–tandem mass spectrometry (LC-MS/MS) (91) in HCMEC^NCL^ ^KD^/HUVEC^NCL^ ^KD^ compared with HCMEC^Control^ ^KD^/HUVEC^Control^ ^KD^ (Figure 9, A–F, and Supplemental Figure 9, A–F). Hierarchical clustering revealed that metabolite levels of HCMEC^NCL^ ^KD^ and HUVEC^NCL^ ^KD^ clearly separated from HCMEC^Control^ ^KD^ and HUVEC^Control^ ^KD^, revealing significantly regulated metabolites between the groups (Figure 9, A and B, and Supplemental Figure 9, A and B). This analysis showed 747 and 383 metabolites altered by the knockdown of NCL in HCMECs and HUVECs, respectively (Figure 9B and Supplemental Figure 9B). Principal component analysis (PCA) further identified specific groups of metabolites, including those involved in endothelial glucose metabolism to be different in HCMEC^NCL^ ^KD^ and HUVEC^NCL^ ^KD^ as compared with the control groups (Figure 9, C and D, and Supplemental Figure 9, C and D).

Next, we examined the metabolic pathways regulated by NCL in ECs. GSEA (76) revealed glycolysis and fatty acid metabolism pathways to be downregulated in HUVEC^NCL^ ^KD^ and fatty acid metabolism pathways to be downregulated in HCMEC^NCL^ ^KD^ (Figure 9E and Supplemental Figure 9E). Moreover, glycolysis was downregulated (even though not significantly) in HCMEC^NCL^ ^KD^ (Figure 9E). We next analyzed the abundance of metabolites involved in glycolysis and other metabolic pathways important for EC homeostasis and activation (89) (Figure 9F, Supplemental Figure 9F, and Supplemental Figure 10). Interestingly, we found that, among the 8 glucose metabolites detected in HCMECs and HUVECs, glyceraldehyde 3-phosphate (G3P), phosphoenolpyruvate (PEP), and lactate (Lac) were significantly decreased in HCMEC^NCL^ ^KD^, whereas Lac was significantly decreased in HUVEC^NCL^ ^KD^ (Figure 9F and Supplemental Figure 9F). Notably, Lac levels were decreased by 35% in HCMEC^NCL^ ^KD^ versus HCMEC^Control^ ^KD^ and by 55% in HUVEC^NCL^ ^KD^ versus HUVEC^Control^ ^KD^ (Figure 9F and Supplemental Figure 9F). Moreover, the NADH/NAD^+^ ratio indicating for metabolic activity (92, 93) showed a decrease (although not significant) upon NCL KD in both cell types (Figure 9F and Supplemental Figure 7F). Together, these results indicate that NCL exerts its positive regulatory effects on CNS sprouting angiogenesis via positive regulation of endothelial (glucose) metabolism.

### NCL regulates endothelial glucose metabolism but not fatty acid metabolism.

**Figure 10:**
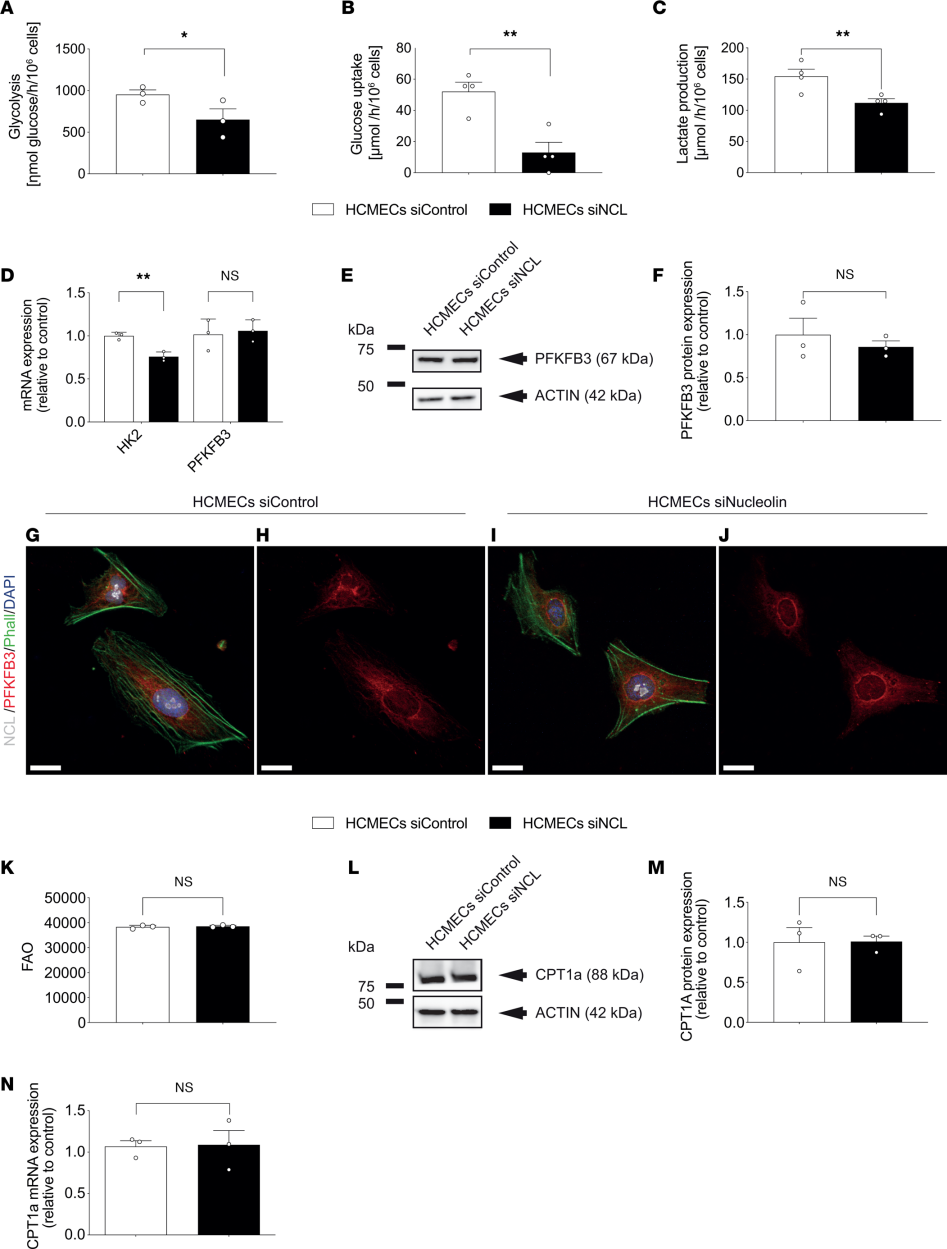
NCL positively regulates endothelial glucose metabolism via glycolytic enzymes, including HK2, but does not affect fatty acid oxidation in human brain endothelial cells. (**A**–**C**) Metabolic assays of HCMECs, upon NCL downregulation with siRNA targeting NCL. NCL knockdown decreased the glycolytic flux (**A**, *n* = 3), glucose uptake (**B**, *n* = 4), and Lac production (**C**, *n* = 4) in HCMECs as compared with the tested controls. (**D**) qPCR revealing a significant downregulation of about 30% of hexokinase-2 (*HK2*) mRNA expression by siRNA-targeted NCL knockdown. *PFKFB3* expression showed slight but no significant increase (*n* = 3). (**E** and **F**) Western blot using antibodies against PFKFB3 revealed no significant regulation of PFKFB3 expression by NCL knockdown (*n* = 3). (**G**–**J**) HCMECs were stained for NCL (green), PFKFB3 (red), and the general nuclear marker DAPI (blue). No difference in PFKFB3 expression could be seen between NCL knockdown HCMECs (**G** and **H**) and the HCMECs treated with a control siRNA (**I** and **J**). (**K**) NCL knockdown in HCMECs did not affect fatty acid oxidation (*n* = 3). (**L** and **M**) Western blot using antibodies against carnitine palmitoyltransferase 1A (CPT1A) showed no difference in CPT1A protein expression between NCL knockdown HCMECs and the control condition (*n* = 3). (**N**) qPCR showed no significant regulation of *CPT1A* mRNA expression upon siRNA-targeted NCL knockdown (*n* = 3). Data represent mean ± SEM. Two-tailed unpaired Student’s *t* test were performed. *P < 0.05, **P < 0.01. Scale bars: 20 μm in **G**–**J**.

To further examine the observed effects of NCL on endothelial metabolism, we next performed functional metabolic assays addressing endothelial glucose and fatty acid metabolism in HCMECs and HUVECs. Using a glycolytic flux assay (94), siRNA-mediated knockdown of NCL resulted in a significant reduction of glycolysis as compared with the control HCMECs and HUVECs (Figure 10A and Supplemental Figure 11A). Similarly, HCMEC^NCL^ ^KD^ and HUVEC^NCL^ ^KD^ showed reduced glucose uptake and decreased Lac production as compared with the HCMEC^NCL^ ^KD^ and HUVEC^Control^ ^KD^ (Figure 10, B and C, and Supplemental Figure 11, B and C), indicating a positive regulatory effect of NCL on HCMEC and HUVEC glucose metabolism.

6-Phosphofructo-2-kinase/fructose-2,6-biphosphatase 3 (PFKFB3) and HK2 have been shown to be key regulators of endothelial glucose metabolism (88, 89). To address whether NCL affected the expression patterns of these genes in HCMECs and HUVECs, we performed qPCR and Western blots of HCMEC^NCL^ ^KD^, HUVEC^NCL^ ^KD^, HCMEC^Control^ ^KD^, and HUVEC^Control^ ^KD^. HK2 was significantly downregulated upon NCL knockdown on the mRNA level (Figure 10D and Supplemental Figure 11D), whereas PFKFB3 showed no significant change on either the mRNA or the protein levels (Figure 10, E–J, and Supplemental Figure 11, E–J).

Next, we assessed whether NCL also regulates endothelial fatty acid oxidation (FAO), which is known to exert crucial effects on endothelial stalk cell proliferation (89, 95) and to be upregulated in quiescent ECs as a protection against oxidative stress (96). NCL knockdown in HCMECs and HUVECs did not affect endothelial FAO (Figure 10K and Supplemental Figure 11K). Carnitine palmitoyltransferase 1A (CPT1A) has been shown to be a key regulator of endothelial fatty acid metabolism (88, 89). As expected, qPCR and Western blot analysis of HCMEC^NCL^ ^KD^ and HUVEC^NCL^ ^KD^ showed no significant differences of CPT1A as compared with the HCMEC^Control^ ^KD^ and HUVEC^Control^ ^KD^ groups at either the mRNA levels and protein levels (Figure 10, L–N, and Supplemental Figure 11, L–N).

Taken together, these data reveal that KD of NCL decreases endothelial glucose metabolism without affecting endothelial fatty acid metabolism, indicating a positive regulatory role of NCL on sprouting angiogenesis via promoting endothelial glucose metabolism.

## Discussion

Here, using in vitro and in vivo approaches, we show that NCL is a positive regulator of angiogenesis in the human fetal brain. Our results suggest that NCL promotes brain endothelial sprouting, proliferation, and filopodia formation, potentially via interaction with the VEGF-A–VEGFR2 pathway and positively regulates brain endothelial glucose metabolism via the regulation of glycolytic enzymes, including HK2. We propose that, by acting on the cytoskeleton of CNS endothelial (tip and stalk) cells and their filopodia, and by regulating vascular endothelial metabolism, NCL controls the sprouting and filopodia extension of growing CNS blood vessels during human fetal brain development and presumably in human brain tumors. Importantly, the characterization of NCL as an oncofetal protein in the brain tumor vasculature and its inhibition using aptamers identifies NCL as a potential pharmaceutical target for gliomas.

### NCL may be a putative NVU/PVN-derived oncofetal signal to regulate developmental brain and brain tumor (vascular) growth.

Most of the evidence regarding the molecular regulation of sprouting angiogenesis during brain development is based on murine studies (97, 98), whereas less knowledge exists regarding how the vascularization and ETCs are regulated in the human brain. Interestingly, angiogenesis is highly dynamic during brain development and almost quiescent in the adult healthy brain (11, 14, 57), but it is reactivated in a variety of angiogenesis-dependent CNS pathologies such as brain tumors, brain vascular malformations, or stroke (4, 5, 14), thereby activating endothelial- and perivascular cells of the NVU (7, 11, 57). In our study, we not only observed a reactivation of NCL in angiogenic endothelial and perivascular cells within glial brain tumors, but we also observed a positive correlation of NCL expression with astrocytic tumor progression, in agreement with ref. 99, suggesting a crucial role of NCL as a PVN-derived signal in both angiogenic and tumor growth. The PVN has been shown to activate tumor growth in mouse and zebrafish models of breast cancer (100). Strikingly, endothelial-derived thrombospondin-1 in the stable microvasculature induced sustained breast cancer cell quiescence, but this suppressive cue was lost in sprouting neovasculature, where ETC-derived active TGF-β1 and periostin promoted breast tumor growth (100). These characterized the stable microvasculature as a “dormant perivascular (tumor) niche” in contrast to the sprouting neovasculature, which constitutes an “activated perivascular (tumor) niche” in which ETCs exert crucial roles. In light of these studies, the exploration of NCL’s angiogenic function within the perivascular tip cell niche in vivo promises to be an exciting avenue for future investigations.

Here, we found that NCL is an important positive regulator of angiogenesis and ETC filopodia in human fetal brain. The expression of NCL in endothelial and perivascular cells such as astrocytes and pericytes within the NVU of the human fetal brain, its downregulation in the adult healthy brain and reactivation in brain tumors characterizes NCL as an oncofetal protein and suggests an integral role once reactivated during brain cancer. Its high expression in CD105^+^ angiogenic blood vessel ECs in both human fetal brain and human GBM (but not in the adult healthy brain) supports this presumed role in active (developmental, tumor) versus stable (adult healthy) brain angiogenesis. Accordingly, our in vitro results suggest that, by exerting stimulatory effects on sprouting angiogenesis, filopodia extension, and glucose metabolism of vascular ECs, NCL might promote the sprouting of angiogenic blood vessel ECs into the brain parenchyma. The latter is also strongly suggested by the positive correlation between NCL expression and the number of ETC filopodia in the fetal brain parenchyma in vivo. Other neurodevelopmental regulators such as VEGF-A and GPR124 are downregulated in the adult healthy CNS and are reactivated in vascular-dependent CNS pathologies such as brain tumors or stroke (101–103). However, in contrast to those angiogenic factors, NCL is one of the first to have been directly compared in both human fetal brain and human gliomas. Furthermore, NCL endothelial and perivascular expression within the fetal- and tumor PVN and its presumed different roles on the involved cell types (angiogenesis versus tumor growth) characterize NCL as an important developmental signal reactivated during brain tumor growth and, consequently, as an oncofetal protein.

### NCL is a regulator of glucose but not fatty acid metabolism and may, therefore, have different effects on endothelial tip and stalk cells during sprouting angiogenesis.

Endothelial metabolism has recently emerged as a crucial regulator of sprouting angiogenesis during development and in tumors (87–89, 95, 104, 105). Moreover, it was suggested that ETCs mainly rely on glycolysis, whereas endothelial stalk cells also use fatty acid metabolism to support proliferation (87–89, 95, 104, 105). Here, we found that NCL positively regulates endothelial glycolysis but not fatty acid metabolism in vitro, indicating that NCL’s main effect might be on tip cells, but NCL’s precise roles on both tip and stalk cells inside and outside the CNS — for instance, in the embryonic or postnatal brain (26, 57) or in the postnatal retina (106) — need to be investigated in vivo (Supplemental Discussion).

As also demonstrated by our bulk RNA-Seq, pathways regulated downstream of NCL were linked to angiogenesis and endothelial glucose metabolism. While the observed regulatory effects of NCL on endothelial glucose metabolism are in line with an important role of glycolysis in angiogenesis and vascular biology inside and outside the CNS (88, 89, 104, 107, 108), we cannot exclude that NCL regulates other metabolic pathways that participate in (brain) angiogenesis and (brain) EC biology. Thus, given the crucial role of endothelial metabolism for vessel sprouting in development and disease (87, 88, 104) as well as of tumor metabolism in gliomas (35), investigating the precise role of NCL on EC metabolism and angiogenesis in the human brain vasculature along the oncofetal axis promises to be exciting.

### A putative role for NCL in angiogenesis-dependent CNS pathologies via molecular crosstalk with the VEGF-VEGFR signaling axis.

Angiogenesis and the PVN exert crucial roles in the pathophysiology of various vascular-dependent CNS diseases such as brain tumors, vascular malformations, and stroke (6, 7, 17, 49, 50, 56). With regard to brain tumors, glycosylated surface NCL has been shown to increase with the malignancy grade of human gliomas (99). A high expression of NCL may, therefore, promote vascularization of astrocytoma and thereby promote brain tumor growth (Supplemental Discussion). Furthermore, the anticancer aptamer AS1411 — that binds specifically to NCL — has shown promising clinical activity and is being widely used as a tumor-targeting agent (72) as well as an inhibitor of pathological angiogenesis in the retina (49, 50). In line with these reports, we find AS1411-mediated inhibition of brain EC proliferation in vitro, indicating that AS1411 could be tested to target the brain tumor vasculature in vivo. Interestingly, antibody- and peptide-mediated targeting of NCL induced normalization of tumor vasculature in pancreas and breast cancer models (53, 69), further suggesting that strategies targeting NCL might affect the GBM vasculature. During (glial) brain tumor progression, vascular dysfunction is partially mediated via angiogenic factors including VEGF-VEGFR (17, 109–111), and blocking VEGF-VEGFR signaling results in transient normalization of the immature and leaky brain tumor vasculature and leads to survival benefits in patients with newly diagnosed as well as recurrent GBM (17, 112, 113). Here, we observed a positive regulatory effect of NCL on the VEGF-A–VEGFR2 pathway in vitro, indicative of a molecular crosstalk between these 2 signaling axes. Given that both pathways regulate angiogenesis and vascular normalization, combinatorial targeting of NCL and VEGF-A–VEGFR2 to normalize the brain tumor vasculature may be a promising antiangiogenic strategy for GBM patients. Taken together, these literature indications in concert with our data suggest that — in addition to its effect on tumor cell proliferation — oncofetal NCL may regulate brain tumor vascularization and could be a candidate for targeted therapy on both tumor and ECs in human gliomas.

## Methods

Supplemental Methods are available online with this article.

### Study approval.

Tissue samples from human fetal brains were obtained from postmortem fetuses derived from spontaneous abortions and received by the Department of Pathological Anatomy, University of Bari School of Medicine. Tissue preparation and storage were performed as previously described. The study was approved by the Ethics Committee of the University of Bari Medical School and complied with the principles stated in the Declaration of Helsinki.

Tissue samples from human patients with GBM and controls from temporal lobes after selective temporal lobectomy in patients with chronic pharmacoresistant mesial temporal lobe epilepsy were obtained during surgery at the Department of Neurosurgery, University Hospital Zurich. Written informed consent was obtained from patients before study entry. All procedures were conducted in accordance with the Declaration of Helsinki, and the study was approved by the Ethics Committee of the Canton Zurich.

## Author contributions

TW had the idea for the study, designed the experiments, wrote the manuscript, and created the figures. TW, MS, IDT, JYS, MG, OS, FG, ME, and MY conducted the experiments and analyzed the data. RS, VV, DV, KDB, and KF supervised the experiments in their respective labs, and TW supervised the research. TW wrote the manuscript. MS assisted in editing the manuscript and figures. JH, OS, AM, SEL, JPG, PPM, IR, KF, RS, VV, DV, and KDB gave critical input to the manuscript. All authors read and approved the final manuscript.

## Supplementary Material

Supplemental data

## Version 1 03/14/2023

In-Press Preview

## Version 2 04/24/2023

Electronic publication

## References

[1] Batchelor TT (2014). Antiangiogenic therapy for glioblastoma: current status and future prospects. Clin Cancer Res.

[2] Hegi ME (2005). MGMT gene silencing and benefit from temozolomide in glioblastoma. N Engl J Med.

[3] Stupp R (2005). Radiotherapy plus concomitant and adjuvant temozolomide for glioblastoma. N Engl J Med.

[4] Carmeliet P, Jain RK (2011). Molecular mechanisms and clinical applications of angiogenesis. Nature.

[5] Jain RK, Carmeliet P (2012). SnapShot: tumor angiogenesis. Cell.

[6] Jain RK (2007). Angiogenesis in brain tumours. Nat Rev Neurosci.

[7] Hjelmeland AB (2011). Twisted tango: brain tumor neurovascular interactions. Nat Neurosci.

[8] Jayson GC (2016). Antiangiogenic therapy in oncology: current status and future directions. Lancet.

[9] Batchelor TT (2007). AZD2171, a pan-VEGF receptor tyrosine kinase inhibitor, normalizes tumor vasculature and alleviates edema in glioblastoma patients. Cancer Cell.

[10] Gilbert MR (2014). A randomized trial of bevacizumab for newly diagnosed glioblastoma. N Engl J Med.

[11] Walchli T (2015). Wiring the vascular network with neural cues: a CNS perspective. Neuron.

[12] Weis SM, Cheresh DA (2011). Tumor angiogenesis: molecular pathways and therapeutic targets. Nat Med.

[13] Sweeney MD (2018). Blood-brain barrier breakdown in Alzheimer disease and other neurodegenerative disorders. Nat Rev Neurol.

[14] Potente M (2011). Basic and therapeutic aspects of angiogenesis. Cell.

[15] Wälchli T (2023). Shaping the brain vasculature in development and disease in the single-cell era. Nat Rev Neurosci.

[16] Walchli T (2021). Hierarchical imaging and computational analysis of three-dimensional vascular network architecture in the entire postnatal and adult mouse brain. Nat Protoc.

[17] Arvanitis CD (2020). The blood-brain barrier and blood-tumour barrier in brain tumours and metastases. Nat Rev Cancer.

[18] Hsu CC (2011). Identifying LRRC16B as an oncofetal gene with transforming enhancing capability using a combined bioinformatics and experimental approach. Oncogene.

[19] Khazamipour N (2020). Oncofetal chondroitin sulfate: a putative therapeutic target in adult and pediatric solid tumors. Cells.

[20] Oo HZ (2021). Oncofetal chondroitin sulfate is a highly expressed therapeutic target in non-small cell lung cancer. Cancers (Basel).

[21] Vladoiu MC (2019). Childhood cerebellar tumours mirror conserved fetal transcriptional programs. Nature.

[22] Couturier CP (2020). Single-cell RNA-seq reveals that glioblastoma recapitulates a normal neurodevelopmental hierarchy. Nat Commun.

[23] Richards LM (2021). Gradient of developmental and injury response transcriptional states defines functional vulnerabilities underpinning glioblastoma heterogeneity. Nat Cancer.

[24] Huijbers EJM (2022). Tumors resurrect an embryonic vascular program to escape immunity. Sci Immunol.

[25] Sharma A (2020). Onco-fetal reprogramming of endothelial cells drives immunosuppressive macrophages in hepatocellular carcinoma. Cell.

[26] Fantin A (2013). The embryonic mouse hindbrain as a qualitative and quantitative model for studying the molecular and cellular mechanisms of angiogenesis. Nat Protoc.

[27] Gerhardt H (2003). VEGF guides angiogenic sprouting utilizing endothelial tip cell filopodia. J Cell Biol.

[28] De Smet F (2009). Mechanisms of vessel branching: filopodia on endothelial tip cells lead the way. Arterioscler Thromb Vasc Biol.

[29] Gilbert MR (2013). Dose-dense temozolomide for newly diagnosed glioblastoma: a randomized phase III clinical trial. J Clin Oncol.

[30] Jakobsson L (2010). Endothelial cells dynamically compete for the tip cell position during angiogenic sprouting. Nat Cell Biol.

[31] Mazzone M (2009). Heterozygous deficiency of PHD2 restores tumor oxygenation and inhibits metastasis via endothelial normalization. Cell.

[32] Bergers G, Benjamin LE (2003). Tumorigenesis and the angiogenic switch. Nat Rev Cancer.

[33] Ribatti D, Djonov V (2012). Intussusceptive microvascular growth in tumors. Cancer Lett.

[34] Frentzas S (2016). Vessel co-option mediates resistance to anti-angiogenic therapy in liver metastases. Nat Med.

[35] Bi J (2020). Altered cellular metabolism in gliomas - an emerging landscape of actionable co-dependency targets. Nat Rev Cancer.

[37] Tajrishi MM (2011). Nucleolin: the most abundant multifunctional phosphoprotein of nucleolus. Commun Integr Biol.

[38] Ginisty H (1999). Structure and functions of nucleolin. J Cell Sci.

[39] Tuteja R, Tuteja N (1998). Nucleolin: a multifunctional major nucleolar phosphoprotein. Crit Rev Biochem Mol Biol.

[40] Srivastava M, Pollard HB (1999). Molecular dissection of nucleolin’s role in growth and cell proliferation: new insights. FASEB J.

[41] Mongelard F, Bouvet P (2007). Nucleolin: a multiFACeTed protein. Trends Cell Biol.

[42] Xu Z (2012). Knocking down nucleolin expression in gliomas inhibits tumor growth and induces cell cycle arrest. J Neurooncol.

[43] Goldshmit Y (2014). Interfering with the interaction between ErbB1, nucleolin and Ras as a potential treatment for glioblastoma. Oncotarget.

[44] Benedetti E (2015). Nucleolin antagonist triggers autophagic cell death in human glioblastoma primary cells and decreased in vivo tumor growth in orthotopic brain tumor model. Oncotarget.

[45] Luo Z (2017). Precise glioblastoma targeting by AS1411 aptamer-functionalized poly (l-γ-glutamylglutamine)-paclitaxel nanoconjugates. J Colloid Interface Sci.

[46] Soundararajan S (2008). The nucleolin targeting aptamer AS1411 destabilizes Bcl-2 messenger RNA in human breast cancer cells. Cancer Res.

[47] Rosenberg JE (2014). A phase II trial of AS1411 (a novel nucleolin-targeted DNA aptamer) in metastatic renal cell carcinoma. Invest New Drugs.

[48] Figueiredo J (2019). AS1411 derivatives as carriers of G-quadruplex ligands for cervical cancer cells. Int J Pharm.

[49] Iturriaga-Goyon E (2021). AS1411 nucleolin-specific binding aptamers reduce pathological angiogenesis through inhibition of nucleolin phosphorylation. Int J Mol Sci.

[50] Vivanco-Rojas O (2020). Corneal neovascularization is inhibited with nucleolin-binding aptamer, AS1411. Exp Eye Res.

[51] Christian S (2003). Nucleolin expressed at the cell surface is a marker of endothelial cells in angiogenic blood vessels. J Cell Biol.

[52] Huang Y (2006). The angiogenic function of nucleolin is mediated by vascular endothelial growth factor and nonmuscle myosin. Blood.

[53] Gilles ME (2016). Nucleolin targeting impairs the progression of pancreatic cancer and promotes the normalization of tumor vasculature. Cancer Res.

[54] Xu C (2019). Targeting surface nucleolin induces autophagy-dependent cell death in pancreatic cancer via AMPK activation. Oncogene.

[55] Suzuki T (1997). DNA staining for fluorescence and laser confocal microscopy. J Histochem Cytochem.

[56] Storkebaum E (2011). Cerebrovascular disorders: molecular insights and therapeutic opportunities. Nat Neurosci.

[57] Walchli T (2015). Quantitative assessment of angiogenesis, perfused blood vessels and endothelial tip cells in the postnatal mouse brain. Nat Protoc.

[58] Daneman R (2010). Pericytes are required for blood-brain barrier integrity during embryogenesis. Nature.

[59] Armulik A (2010). Pericytes regulate the blood-brain barrier. Nature.

[60] Roitbak T (2008). Neural stem/progenitor cells promote endothelial cell morphogenesis and protect endothelial cells against ischemia via HIF-1alpha-regulated VEGF signaling. J Cereb Blood Flow Metab.

[61] Chou CH (2014). In vitro modeling of the neurovascular environment by coculturing adult human brain endothelial cells with human neural stem cells. PLoS One.

[62] Louis DN (2007). The 2007 WHO classification of tumours of the central nervous system. Acta Neuropathol.

[63] Louis DN (2016). The 2016 World Health Organization classification of tumors of the central nervous system: a summary. Acta Neuropathol.

[64] Perry A, Wesseling P (2016). Histologic classification of gliomas. Handb Clin Neurol.

[65] Gerdes J (1984). Cell cycle analysis of a cell proliferation-associated human nuclear antigen defined by the monoclonal antibody Ki-67. J Immunol.

[66] Ishii N (1999). Frequent co-alterations of TP53, p16/CDKN2A, p14ARF, PTEN tumor suppressor genes in human glioma cell lines. Brain Pathol.

[67] Diserens AC (1981). Characterization of an established human malignant glioma cell line: LN-18. Acta Neuropathol.

[68] Destouches D (2008). Suppression of tumor growth and angiogenesis by a specific antagonist of the cell-surface expressed nucleolin. PLoS One.

[69] Fogal V (2009). Cell surface nucleolin antagonist causes endothelial cell apoptosis and normalization of tumor vasculature. Angiogenesis.

[70] Korff T (2004). Three-dimensional spheroidal culture of cytotrophoblast cells mimics the phenotype and differentiation of cytotrophoblasts from normal and preeclamptic pregnancies. Exp Cell Res.

[71] Cheng Y (2016). AS1411-induced growth inhibition of glioma cells by up-regulation of p53 and down-regulation of Bcl-2 and Akt1 via nucleolin. PLoS One.

[72] Reyes-Reyes EM (2015). Mechanistic studies of anticancer aptamer AS1411 reveal a novel role for nucleolin in regulating Rac1 activation. Mol Oncol.

[73] Kuo CW (2010). Polymeric nanopillar arrays for cell traction force measurements. Electrophoresis.

[74] Shiu JY (2018). Nanopillar force measurements reveal actin-cap-mediated YAP mechanotransduction. Nat Cell Biol.

[75] O’Sullivan T (2014). Interleukin-17D mediates tumor rejection through recruitment of natural killer cells. Cell Rep.

[76] Subramanian A (2005). Gene set enrichment analysis: a knowledge-based approach for interpreting genome-wide expression profiles. Proc Natl Acad Sci U S A.

[77] Reimand J (2019). Pathway enrichment analysis and visualization of omics data using g:Profiler, GSEA, Cytoscape and EnrichmentMap. Nat Protoc.

[78] Wang Y (2015). KLF4 promotes angiogenesis by activating VEGF signaling in human retinal microvascular endothelial cells. PLoS One.

[79] Liabotis A (2022). Angiopoietin-like 4-induced 3D capillary morphogenesis correlates to stabilization of endothelial adherens junctions and restriction of VEGF-induced sprouting. Biomedicines.

[80] Thomas M, Augustin HG (2009). The role of the angiopoietins in vascular morphogenesis. Angiogenesis.

[81] Balaji Ragunathrao VA (2019). Sphingosine-1-phosphate receptor 1 activity promotes tumor growth by amplifying VEGF-A–VEGFR2 Angiogenic Signaling. Cell Rep.

[82] DeWaal D (2018). Hexokinase-2 depletion inhibits glycolysis and induces oxidative phosphorylation in hepatocellular carcinoma and sensitizes to metformin. Nat Commun.

[83] Lebherz HG, Rutter WJ (1969). Distribution of fructose diphosphate aldolase variants in biological systems. Biochemistry.

[84] Vizin T, Kos J (2015). Gamma-enolase: a well-known tumour marker, with a less-known role in cancer. Radiol Oncol.

[85] Feng Y (2020). A20 targets PFKL and glycolysis to inhibit the progression of hepatocellular carcinoma. Cell Death Dis.

[86] Lee JH (2017). Stabilization of phosphofructokinase 1 platelet isoform by AKT promotes tumorigenesis. Nat Commun.

[87] Eelen G (2013). Control of vessel sprouting by genetic and metabolic determinants. Trends Endocrinol Metab.

[88] De Bock K (2013). Role of PFKFB3-driven glycolysis in vessel sprouting. Cell.

[89] Rohlenova K (2018). Endothelial cell metabolism in health and disease. Trends Cell Biol.

[90] Cruys B (2016). Glycolytic regulation of cell rearrangement in angiogenesis. Nat Commun.

[91] Paglia G (2014). Ion mobility derived collision cross sections to support metabolomics applications. Anal Chem.

[92] Ying W (2008). NAD+/NADH and NADP+/NADPH in cellular functions and cell death: regulation and biological consequences. Antioxid Redox Signal.

[93] Houtkooper RH (2010). The secret life of NAD+: an old metabolite controlling new metabolic signaling pathways. Endocr Rev.

[94] Aragones J (2008). Deficiency or inhibition of oxygen sensor Phd1 induces hypoxia tolerance by reprogramming basal metabolism. Nat Genet.

[95] Schoors S (2015). Fatty acid carbon is essential for dNTP synthesis in endothelial cells. Nature.

[96] Kalucka J (2018). Quiescent endothelial cells upregulate fatty acid β-oxidation for vasculoprotection via redox homeostasis. Cell Metab.

[97] Mancuso MR (2008). Developmental angiogenesis of the central nervous system. Lymphat Res Biol.

[98] Risau W (1997). Mechanisms of angiogenesis. Nature.

[99] Galzio R (2012). Glycosilated nucleolin as marker for human gliomas. J Cell Biochem.

[100] Ghajar CM (2013). The perivascular niche regulates breast tumour dormancy. Nat Cell Biol.

[101] Kuhnert F (2010). Essential regulation of CNS angiogenesis by the orphan G protein-coupled receptor GPR124. Science.

[102] Chang J (2017). Gpr124 is essential for blood-brain barrier integrity in central nervous system disease. Nat Med.

[103] Plate KH (1992). Vascular endothelial growth factor is a potential tumour angiogenesis factor in human gliomas in vivo. Nature.

[104] Cantelmo AR (2016). Inhibition of the glycolytic activator PFKFB3 in endothelium induces tumor vessel normalization, impairs metastasis, and improves chemotherapy. Cancer Cell.

[105] Eelen G (2015). Endothelial cell metabolism in normal and diseased vasculature. Circ Res.

[106] Pitulescu ME (2010). Inducible gene targeting in the neonatal vasculature and analysis of retinal angiogenesis in mice. Nat Protoc.

[107] Veys K (2020). Role of the GLUT1 glucose transporter in postnatal CNS angiogenesis and blood-brain barrier integrity. Circ Res.

[108] De Bock K (2013). Role of endothelial cell metabolism in vessel sprouting. Cell Metab.

[109] Engelhardt S (2014). Cell-specific blood-brain barrier regulation in health and disease: a focus on hypoxia. Br J Pharmacol.

[110] Argaw AT (2006). IL-1beta regulates blood-brain barrier permeability via reactivation of the hypoxia-angiogenesis program. J Immunol.

[111] Quail DF, Joyce JA (2017). The microenvironmental landscape of brain tumors. Cancer Cell.

[112] Batchelor TT (2013). Improved tumor oxygenation and survival in glioblastoma patients who show increased blood perfusion after cediranib and chemoradiation. Proc Natl Acad Sci U S A.

[113] Emblem KE (2013). Vessel architectural imaging identifies cancer patient responders to anti-angiogenic therapy. Nat Med.

