# Supplementary material for: Nucleolin promotes angiogenesis and endothelial metabolism along the oncofetal axis in the human brain vasculature
Source: JCI Insight. 2023 Apr 24;8(8):e143071. doi: 10.1172/jci.insight.143071 (PMC10243742; doi:10.1172/jci.insight.143071)
Supplement: Supplemental data [file jciinsight-8-143071-s197.pdf]

SUPPLEMENTARY INTRODUCTION

Glioblastoma (GBM) is a type of glial brain tumor with a putative stem cell origin (1, 2), and represents the most common malignant primary brain tumors (3). Standard treatment of care involves maximal surgical resection followed by chemotherapy and radiotherapy (4, 5). GBM almost invariably recurs and there is currently no therapy that prolongs survival after tumor recurrence. To achieve significant improvements in GBM therapy and clinical outcomes, a better understanding of the cellular and molecular mechanisms that drive GBM growth, propagation, and therapeutic resistance is required.

Behind the endothelial tip cells, endothelial stalk cells proliferate to further elongate the vascular sprout (6, 7), and quiescent endothelial phalanx cells line the parental vessel (8). After fusion of neighboring sprouts, pericytes and perivascular astrocytes are recruited to stabilize the vessel sprout and allow the formation of functional BBB microvessels (9-12). Interestingly, ETCs drive sprouting angiogenesis during both development and in tumors (7, 13-16). ETCs have been shown to exert crucial regulatory effects via interaction with tumor cells within the perivascular niche in tumors outside the CNS (17, 18).

Upregulation of Nucleolin in CNS and non-CNS cancer cells is associated with poor prognosis and promotes cell proliferation and rDNA transcription to sustain high levels of protein synthesis (19-21, 22,, 23-25). Furthermore, Nucleolin was found in GBM stem cells (26, 27) and may constitute a histopathological marker for glioma grading and a possible tool for targeted therapy (27).

SUPPLEMENTARY RESULTS

Bulk RNA sequencing (RNAseq) reveals regulation of angiogenic pathways including VEGF-A-VEGFR2 as well as of endothelial metabolism upon Nucleolin knock-down

Among the top-regulated genes in HUVECs$^{Nucleolin\ KD}$ figured genes implicated in cell proliferation and cell cycle progression (Supplementary Figure 5E), in agreement with previously reported roles of Nucleolin (22, 28, 29) .

In HUVEC$^{Nucleolin\ KD}$, enrichment map revealed the TP53 pathway to be the only pathway enriched, whereas pathways downregulated in HUVEC$^{Nucleolin\ KD}$ mainly belonged to cell cycle and proliferation (Supplementary Figure 5F), indicating the negative regulatory effect of Nucleolin on TP53 signaling and its positive regulatory effects on cell cycle and proliferation. We then examined genes driving the enrichment of regulation of the TP53 pathway and cell cycle in peripheral ECs. The expression of TP53-induced glycolysis and apoptosis regulator (TIGAR), a protein linking cell cycle and glucose metabolism (30, 31), and the TP53 inducible protein 3 (TP53I3) were upregulated upon Nucleolin knockdown, without affecting TP53 expression (Supplementary Figure 7A-C). In HCMEC$^{Nucleolin\ KD}$, TP53 was significantly downregulated whereas TIGAR and TP53I3 expression were not changed (Supplementary Figure 8A,B). Validation by Western blot and immunofluorescence confirmed Nucleolin-induced upregulation of TIGAR at the protein level in HUVECs but not in HCMECs (Supplementary Figure 7D-J and 8C-I), indicating different underlying mechanisms between HCMECs and HUVECs.

Nucleolin knock-down downregulated *VEGFA* but upregulated *VEGFR2* in HCMEC$^{Nucleolin\ KD}$ and HUVEC$^{Nucleolin\ KD}$ (Figure 8C, Supplementary Figure 6C). Furthermore, the Notch receptors 1 (*NOTCH1*) and 4 (*NOTCH4)*, the Notch downstream effectors *HES1* and *HES2*, the Notch ligands *DLL4* and Jagged 1 (*JAG1*) (Figure 8D, Supplementary Figure 6D) as well as the Hippo-YAP-TAZ pathway component YAP1 and downstream effectors *CTGF* and *CYR61* were regulated upon

Nucleolin knock-down in HCMEC$^{Nucleolin\ KD}$ and HUVEC$^{Nucleolin\ KD}$ (Figure 8E, Supplementary Figure 6E).

SUPPLEMENTARY DISCUSSION

The onco-fetal axis including Nucleolin as novel therapeutic target in the brain tumor vasculature?

In order to achieve a better understanding of brain tumor biology and treatment, a thorough assessment of the tumor microenvironment (TME) comprising the extracellular matrix as well as blood vessel endothelial- and perivascular cells is required (9, 32-38). Notably, the vascular and immune components of the TME are of particular relevance with regard to achieving improvements in the treatment of brain tumors (36, 39).

Recent evidence indicates that brain tumors are a consequence of aberrant brain development or repair and that tumors reactivate onco-fetal programs in- and outside the tumor vasculature (9, 32-36). Interestingly, tumors resurrect an embryonic vascular program to escape immunity. While angiogenesis-induced suppression of adhesion molecules provides embryos with immune-privileged conditions allowing for uninterrupted growth and development, increasing evidence indicates that tumors refer to similar mechanisms to evade the immune system by enforcing a state of unresponsiveness of the tumor vasculature to inflammatory cytokines resulting in lack of adhesiveness of angiogenic ECs for immune cells. This in consequence leads to compromised immunity thereby ensuring progressive tumor growth. Thus, tumors may enforce an embryonic-like gene expression program in ECs via angiogenesis in order to suppress leukocyte infiltration and thereby compromise antitumor immunity. In consequence, angiogenesis inhibitors may overcome this state of compromised immunity and thereby improve immunotherapy (40). Blood vessels of both the developing and tumor vasculature regulate expression of endothelial adhesion molecules to control immune cell infiltration to assure undisturbed growth. Thus, based on the observed expression of Nucleolin in the developing fetal and glial brain tumor vasculature and

given Nucleolin's presumed role as a binding protein of the adhesion molecule P-selectin (41-43), it is tempting to speculate that counteracting Nucleolin's pro angiogenic role might contribute to improved immunotherapies for brain tumors (44).

Expression patterns of Nucleolin in the developing and adult brain as well as in brain tumors

Nucleolin is known to be expressed either at the cell surface, in the cytoplasm, nucleoplasm, or in the nucleolus (29, 45-48).

Here, we observed that Nucleolin expression in the adult brain was mainly restricted to the nucleoli, as opposed to the predominant nucleoplasm expression during brain development and in brain tumors. It is well known that the functions of Nucleolin depend on its subcellular localization, which can be at the cell plasma membrane, in the cytoplasm, in the nucleus (nucleoplasm and/or nucleolus), and that nucleolar fraction of Nucleolin usually represents more than 90% of the cellular pool of Nucleolin (48). The nucleolar restriction of Nucleolin in the adult brain may indicate that its main functions in the adult brain are those not linked to cell proliferation and cell division, such as ribosomal biogenesis and rRNA synthesis allowing for protein synthesis thereby supporting basic cellular functions (29, 45, 46, 48). However, during brain development and in brain tumors, perivascular- and endothelial cells are dividing at high rates and may thus require both, Nucleolin function in the ribosomes (in the nucleolus) as well as its regulatory role on cell cycle and cell proliferation (in the nucleoplasm) (48). Indeed, nuclear/nucleoplasmic Nucleolin is involved in the regulation of oncogene gene expression, promotes the proliferation and aggressiveness of a variety of tumors (48), and protects cancer cells from senescence (48).

In line with these observations, we observed a spatial restriction of Nucleolin to the nucleolus upon Nucleolin knocking-down in vitro, further indicating a restriction on specific basic functions in conditions with decreased Nucleolin expression (such as in the adult brain in vivo). Moreover, even though we observed nucleolar/nucleoplasmic and nucleolar expression, we did not observe

cytosolic or cell surface/plasmalemmal expression of Nucleolin, which might be related to specific organ (e.g. brain versus periphery) and developmental (e.g. brain development, adult brain, brain tumors) properties. Given the complexity of Nucleolin's subcellular expression patterns in various cell types (48), the different functions of Nucleolin in the context of its cellular expression and specific subcellular localization await further investigations. Moreover, in light of the BBB heterogeneity at the single-cell level (34, 49) and based on the observed Nucleolin expression pattern in spatial transcriptomics, future studies should address the expression of Nucleolin in the human brain vasculature along the onco-fetal axis at single-cell resolution.

Nucleolin as a regulator of endothelial filopodia dynamics and actin cytoskeleton orientation

Our in vitro results indicate that the effects of Nucleolin on developmental CNS angiogenesis are mediated via positive regulatory effects on endothelial sprouting, -proliferation, -glucose metabolism -and tip cell filopodia formation. This is supported by our in vivo findings showing Nucleolin expression in endothelial tip- stalk- and phalanx cells. Whereas our data indicate a role for Nucleolin in both tip cell filopodia formation as well as in stalk cell proliferation, the relative importance of Nucleolin for those angiogenic cell types and cellular mechanisms in vivo and in vitro remains unclear. For instance, differential expression patterns of Nucleolin might influence the competitiveness for the tip cell position (16, 50) and Nucleolin's role on tip versus stalk cell specification could be tested using in vitro genetic mosaic sprouting assays (16) and *in silico* computational simulation (50, 51). These future experiments might also shed light on the molecular mechanism that are responsible for our interesting observation of a positive correlation of Nucleolin expression and the number of filopodia extension per ETC during brain development in vivo. It will be exciting to further investigate Nucleolin role on migrating tip cells, proliferating stalk cells and tip vs. stalk cell specification in vivo in both health and disease.

During sprouting angiogenesis and vessel branching, actin cytoskeleton orientation is crucial for endothelial tip- and stalk cell filopodia- and lamellipodia formation, sprout formation and guidance, and cell migration (7, 12, 52-55). For instance, actin fibers form filopodia and lamellipodia in ETCs during sprout migration and actin polarizes in the direction of migration (12, 53-55). The knock-down of Nucleolin in HCMECs and HUVECs lead to disorientation of the actin cytoskeleton inducing a loss of cell polarization as indicated by a change in cell shape towards a less elongated phenotype. Recent evidence suggests that MST1–FOXO1-mediated ETC polarity facilitates sprouting angiogenesis (56). MST1-FOXO1 was shown to be essential for directional migration of tip cells towards hypoxic regions and endothelial-specific deletion of either MST1 or FOXO1 lead to loss of ETC polarity resulting in impaired sprouting angiogenesis (56). Indeed, in our bulk RNA sequencing data, we found regulation of both FOXO1 and MST-1 upon Nucleolin knock-down to different degrees in HCMECs and HUVECs, indicating a complex regulatory role for Nucleolin on MST1-FOXO1-mediated regulation of cell polarity, thereby warranting future investigation. Also, it remains to be determined whether Nucleolin regulates endothelial polarization via interaction with other signaling pathways in brain development and brain tumors in vivo.

Cell polarization directly depends on actin cytoskeleton organization (57) and Nucleolin affected actin cytoskeleton orientation in our study. However, the molecular mechanisms behind the loss of actin cytoskeleton orientation upon knocking-down Nucleolin remain to be elucidated. They could either be mediated via direct effects on the actin cytoskeleton given its well-known functions in cell migration, -sprouting, and lamellipodia- and filopodia formation (58, 59). Alternatively, Nucleolin could affect protein-protein interactions important for actin cytoskeleton regulation such as actin-myosin interactions. Interestingly, glycolytic enzymes including PFKFB3 associate with actin in endothelial filopodia and lamellipodia to provide the high amounts of ATP required for actin-myosin contraction (54, 60). Therefore, the decreased endothelial glycolysis observed in Nucleolin-deprived HCMECs and HUVECs might be causally linked to the decreased force

applied by HCMEC$^{Nucleolin\ KD}$ and HUVEC$^{Nucleolin\ KD}$ filopodia, e.g. due to reduced actin-myosin contraction. Furthermore, in light of emerging evidence of organ-specific regulation of angiogenesis (12, 53, 61), it is tempting to speculate about an organ- or even CNS-specific regulation of Nucleolin on angiogenesis and ETC polarity.

Nucleolin as regulator of glucose-, but not fatty acid endothelial metabolism – effects on endothelial tip- and stalk cells?

On the one hand, these findings are supported by the regulatory effects of Nucleolin on the number of filopodia during fetal brain development. In addition, the regulatory effect of Nucleolin on the actin cytoskeleton in vitro might – at least in part - explain the positive correlation between Nucleolin and the number of filopodia observed in vivo. Filopodia formation relies on actin cytoskeleton orientation and glycolytic production of ATP has been shown to promote filopodia formation in vivo, in vitro, and in silico (50). On the other hand, given the observed in vivo expression of Nucleolin in both endothelial tip- and stalk cells as well as the positive regulatory effect on both sprouting angiogenesis (a tip cell function) and endothelial proliferation (a stalk cell function) in vitro.

Moreover, the knock-down of Nucleolin lead to a downregulation of VEGF-A at the mRNA level as well as to a decrease of p-VEGFR-2 at the protein level, indicating a potential crosstalk between Nucleolin and VEGF-A-VEGFR2). The seemingly contradictory finding that VEGFR2 mRNA was upregulated in Nucleolin KD HCMECs and HUVECs may be due to the complexity of VEGF – VEGFR interactions also involving compensatory mechanism (62) as well as due to translational modifications (63, 64). In light of the important role of this signaling system for CNS angiogenesis (65) and ETCs (8, 13, 49, 66, 67), this result is interesting. However, whether and where the VEGF- and the Nucleolin signaling pathways interact intracellularly in vivo awaits further investigation,

with potential implications for Nucleolin as a therapeutic target for malignant gliomas (9, 32-36, 68).

A putative role for Nucleolin in angiogenesis-dependent CNS pathologies via molecular crosstalk with the VEGF-VEGFR signaling axis?

Based on our findings, one may therefore speculate that Nucleolin could - in addition to brain tumors - also regulate vessel sprouting for example in brain vascular malformations such as arteriovenous- or cavernous malformations, or in neuroregenerative (e.g. stroke), or -degenerative vascular diseases (e.g. vascular- or Alzheimer's dementia).

Moreover, it was reported that delivery of a toxin directed to cell surface Nucleolin could be used as targeted therapy of human glioblastoma (69). Another study reported that a Nucleolin antagonist induced death of primary human glioblastoma cells and decreased in vivo tumor growth in an orthotopic brain tumor model, but effects on angiogenesis were not addressed (70).

SUPPLEMENTARY MATERIAL AND METHODS

Human fetal, adult brain and brain tumor tissue

Samples of fetal brain were obtained from three post-mortem fetuses, two 22- and one 18-week-old, derived from spontaneous abortions and received by the Department of Pathological Anatomy, University of Bari School of Medicine. Permission to collect fetal tissue was obtained from the mother at the end of the abortion procedure. The sampling and handling of the specimens conformed to the ethical rules of the Department of Emergency and Organ Transplantation, Division of Pathology, University of Bari School of Medicine, and approval was gained from the local Ethics Committee of the National Health System in compliance with the principles stated in the Declaration of Helsinki. The fetuses did not reveal macroscopic structural abnormalities at autopsy and/or microscopic malformations of the central nervous system after conventional histological analysis with H&E or toluidine blue staining. The fetal age was estimated based on the crown-rump length and/or pregnancy records (counting from the last menstrual period). From each fetus, samples of the dorso-lateral wall of the telencephalic vesicles (n=6; future cerebral hemispheres) were dissected along the coronal plane in slices about 0.5-cm thick, fixed for 2–3 hours at 4°C by immersion in 2% paraformaldehyde (PFA) plus 0.2% glutaraldehyde in phosphate-buffered saline solution (PBS, pH 7.6), washed in PBS and stored in PBS plus 0.02% PFA at 4°C. The parahippocampal cortex, used as normal adult brain samples obtained after selective amygdalohippocampectomy from patients with chronic pharmaco-resistant mesial temporal lobe epilepsy and glioblastoma samples were also cut in 0.5-cm thick slices and submitted to the same histological procedure applied to fetal slices.

Immunofluorescence staining and analysis of angiogenesis and perivascular niche in human fetal brain, human adult brain, and human brain tumors

Fixed, unembedded fetal brain, normal adult brain, and glioblastoma slices were cut in 20-μm thick sections, using a vibrating microtome (Leica Microsystem) and submitted, as free-floating sections, for single and double staining, with the following antibodies (Abs): mouse mAb anti-Nucleolin (1:200, Santa Cruz, cat. sc-55486), rabbit pAb anti-CD31 (1:80; Abcam, cat. ab28364), rabbit pAb anti-CD105 (predilute; Abcam, ab27422), rabbit pAb anti-GFAP (prediluted, Immunostar, cat. 22522), pAb anti-NG2 (1:200, generous gift from William B. Stallcup). Briefly, the sections were: 1) permeabilized with 0.5% Triton X-100 in PBS for 30 min at room temperature (RT); 2) incubated overnight at 4°C with primary Abs, Nucleolin, Nucleolin/CD31, Nucleolin/CD105, Nucleolin/GFAP, and Nucleolin/NG2) incubated with the appropriate secondary Abs, goat anti-rabbit Alexa 568 and goat anti-mouse Alexa 488 (1:300, ThermoFisher Scientific,), for 45 min at RT; 4) counterstained with the nuclear dye TO-PRO-3 (diluted 1:10'000; Life Technologies, Inc., Gaithersburg, MD, USA). Finally, the sections were collected on polylysine slides (Menzel-Glaser, GmbH, Braunschweig, Germany) and coverslipped with Vectashield (Vector Laboratories Inc.). Negative controls were prepared by omitting the primary antibodies and mismatching the secondary antibodies. Sections were examined under Leica TCS SP5 confocal laser scanning microscope (Leica Microsystems) using a sequential scan procedure. Confocal images were taken at 0.35 μm intervals through the z-axis of the section, with 40x and 63x oil immersion lenses. Z-stacks of optical planes (image projection) and single optical planes were recorded and analysed by Leica confocal software

Laser confocal morphometry

The quantitative assessment was carried out on fetal brains (n=3), normal brains (n=3) and glioblastomas (n=4) samples, by computer-aided morphometric analysis using the Leica Confocal Multicolor Package (Leica Microsystems) and ImageJ (NIH) softwares. The Nucleolin mean area percentage was evaluated according to the 'mean area fraction' parameter of ImageJ, calculated on

randomly chosen fields (n=10; field area = 150.000 μm^2) of single optical planes across the z-stack. On double stained Nucleolin/CD105 sections (fetal brain n=18, normal brain n=12, glioblastoma n=36), cells positive for both the markers were counted on maximum intensity projection images (40x magnification), cells positive for both Nucleolin and CD31 were counted on double stained sections (fetal brain n=18, normal brain n=18, glioblastoma n=24), $Nucleolin^+/GFAP^+$ cells were counted on double stained sections (fetal brain n=12, normal brain n=6, glioblastoma n=10), and $Nucleolin^+/NG2^+$ cells were counted on double stained sections (fetal brain n=15, normal brain n=9, glioblastoma n=29). The percentage of double positive cells was calculated as the fraction of these cells on total number of nuclei stained with TO-PRO-3 counted on optical planes at 0.35µm intervals across the z-stack.

The measurement of the number of vessel sprouts was carried out on Nucleolin/CD31 and Nucleolin/CD105 stained sections (n=3 for each of the 3 fetus analysed), to make the presence of endothelial tip cells recognizable. The results showed a range of 2-6 endothelial tip cells per section (total endothelial tip cell number, n=35). The Nucleolin green signal intensity was measured by manual selection of the cell nucleus as a ROI. The number of filopodia emerging from each vessel sprout was recognized and counted on single optical planes acquired at 63x.

Tissue Microarrays (TMAs)

Glioma tissue microarray. The study cohort comprised of low- and high-grade glioma samples from 103 patients who were treated at the Department of Neurosurgery, University Hospital Zurich (Switzerland) between 06/2003 and 05/2009. Paraffin blocks of these tumors were reviewed by a neuropathologist and classified according to the World Health Organization of brain tumors (71, 72). Representative tumor areas were marked on hematoxylin/eosin-stained slides by an experienced neuropathologist (JH) and two cores (0.6 mm diameter) were punched from the donor

block and transferred into the tissue microarray (TMA) recipient block. Additionally, the TMA contained eight normal brain samples and four cell lines (MCF-7, LN-18, LN-229 and HT29).

For immunohistochemistry (IHC) analysis freshly cut 3-μm thick sections of the TMA block were mounted on SuperFrost. slides (Menzel Glaser, Braunschweig, Germany). The mouse monoclonal Nucleolin antibody C23 (1:20, Santa Cruz cat. sc-55486) was used and incubated overnight at 4°C. As a detection system the Simple Stain MAX PO (MULTI) anti-mouse (Nichirei Biosciences) was used. Finally, the slides were counterstained with Mayer's hemalum solution prior to dehydration and coverslipping.

Percentage of Nucleolin staining of tumor cells in all the samples were evaluated by two investigators and was performed in an entirely blinded fashion.

Cell culture and proliferation assay

Glioma cell lines (LN-229 and LN-18 kindly provided by N. de Tribolet, University of Geneva, Geneva, Switzerland) and the ex vivo GBM-1 cells (passage 3-7) established at the Department of Neurosurgery, University Hospital Zurich, as described by Rodak et al.(73) were cultured in DMEM (Gibco, Thermo Fisher Scientific, Allschwil, Switzerland) containing 10% heat-inactivated FCS (PAA Laboratories, Thermo Fisher Scientific), gentamicin (10 μg/ml, Gibco), 100 mM sodium pyruvate (ICN Biomedicals, Aurora, Ohio), and 5 ml N-acetyl L-alanyl L-glutamine (Biochrom AG, Berlin, Germany).

Brain endothelial cells (HCMECs, kindly provided by Prof. Weller, University of Zurich, University Hospital of Zurich, Zurich, Switzerland) and HUVECs (Lonza) were cultured in endothelial basal medium (EBM-2, Lonza) supplemented with endothelial growth factors EGM-2 SingleQuots (Lonza).

siRNA-mediated knockdown of Nucleolin

Silencing of Nucleolin expression, was performed by transfecting glioma cell line (LN-229, LN18), the ex vivo GBM-1 cells and endothelial cells (HCMECs, HUVECs) with siRNA against human Nucleolin (50nM, Santa Cruz), or a control siRNA (50nM, Santa Cruz), as indicated by the manufacturer. HUVECs were transfected with the indicated siRNA using Transfection reagent and Transfection medium (Santa Cruz).

Proliferation assay

Glioma cells (LN-229, LN-18, GBM-1), HCMECs and HUVECs were incubated for 6-7 hours in growth medium supplemented with 1 μCi/ml [^{3}H]-thymidine. Cells were fixed with 100% ethanol for 15 min at 4°C. Thereafter, cells were precipitated with 10% trichloroacetic acid and lysed using 0.1N NaOH. [^{3}H]-thymidine incorporation into DNA was measured by scintillation counting (Wallac 1450 Microbeta/Trilux, Shelton, USA).

Immunoblotting

Cells were lysed in RIPA buffer and mechanical disruption through a 1ml insulin syringe (BD). Proteins were separated by 10% SDS-polyacrylamide gel electrophoresis (SDS-PAGE) at 150 V and transferred to a nitrocellulose membrane at 100 V for 1.5 hours. Unspecific binding sites were blocked with 5% milk in TBS-Tween 0.05% for 30 minutes. The membrane was probed with mouse anti-Nucleolin (NCL, 1:500, Santa Cruz cat. sc-55486), rabbit anti-6-phosphofructo-2-kinase/fructose-2,6-biphosphatase 3 (PFKFB3, 1:1,000, abcam cat. ab127894), rabbit anti-CPT1a (1:500, abcam cat. ab128568), and rabbit anti-TIGAR (1:200, Sigma cat. HPA040354) at 4°C overnight. A secondary donkey antibody directed against mouse, or rabbit (1:2,000, Dako, cat. P044801-2 and P044701-2) was applied for 1 hour. Bands were visualised by chemiluminescence using ECL (Thermo Fisher, cat. 32132). Densitometric analysis was performed with ImageJ (NIH freeware). Data were normalised to actin, and values of control cells were set to 1.

Quantitative real-time PCR

Total RNA was prepared using the RNeasy RNA isolation kit (Qiagen, Hilden, Germany) including a DNase treatment to digest residual genomic DNA. For reverse transcription, equal amounts of total RNA were transformed by oligo(dT) and M-MLV reverse transcriptase (Promega). Ten nanograms of cDNA were amplified in the Applied Biosystems 7500 Fast Real Time PCR System thermocycler (Thermo Fisher Scientific) with the polymerase ready mix (KAPA SYBR FAST; Sigma). Relative quantification was calculated using the comparative threshold cycle ($\Delta\Delta^{CT}$) method. cDNA levels were normalized to *S18* (reference genes) and a control sample (calibrator set to 1) was used to calculate the relative values.

Spheroid angiogenesis assay

A three-dimensional (3D) in vitro sprouting angiogenesis assay was performed as described previously (60, 74). Briefly, HUVECs were incubated overnight in hanging drops in EGM-2 medium containing 20% methylcellulose (Sigma) to form spheroids. Spheroids were then embedded in collagen type I gel containing VEGF-A, basic FGF (bFGF), and EGF and cultured for 24 hours (at 37°C, 5% CO2) in EGM-2 to induce sprouting. Spheroids were fixed with 4% PFA at room temperature for 15 min and images of spheroids were captured with a Leica DMi1 (objectives: 20x and 40x. Analysis of the number of sprouts and the average sprout length was done using Image J.

Aptamer treatment

HCMECs were treated for 96h with 0uM (PBS), 1.25uM, 5uM and 10uM of the Nucleolin targeting aptamer AS1411 or the control aptamer CRO (Creative Biolabs, . At endpoint, cells were washed and stained with 0.5% Crystal violet in 20% methanol for 20min at room temperature. Cells were

then washed 3 times with tap water and left to dry for 24h. The cell-bound dye was redissolved in 10% SDS and the optical density was measured at λ = 570 nm with a a Sunrise plate reader (Tecan; Männedorf, Switzerland). The absorbance values were normalized to PBS treatment.

Nanopillar arrays

The polymer nanopillars platforms (photoresistant SU-8 nanopillars, Micro Chem) were fabricated using nanosphere lithography followed by a molding process, as previously described (59, 75). The spring constant k of a representative nanopillars was measured by atomic force microscopy (AFM). From the resulting force curve, k was calculated resulting in a value of 79 nN um^{-1}. To enable HCMECs/HUVECs culture on nanopillars, arrays were placed on petri dishes. HCMECs/HUVECs on nanopillars deflected the nanopillar tips (displacement x) and the resulting forces exerted by HCMECs/HUVECs on nanopillars could be dedicated using the formula F=k*x.

Printing Fn on nanopillar substrates for cell culture

The flat PDMS stamps were plasma treated for 5 seconds before use. 50 µg/ml fibronectin labelled with Alexa dye 633 were added to the stamp and was incubated at room temperature for 15 minutes. Excess Fn solution was removed and the stamp was dried by compressed air. Stamps coated with Fn were applied on top of the nanopillar substrates, 50g weight was applied on top to ensure good contact. The stamps were removed from nanopillar substrates and non-coated area was blocked with Pluronic F-127 (P2443, Sigma) solution. Control HUVEC and HUVEC Nucleolin KD were cultured in the EGM-2 medium (Lonza), at 37°C with 5% CO_2. Cells (7,500 cells/$cm^{2)}$ were let to adhere for 6 h seeded on a SU-8 nanopillar array printed with fibronectin.

Image acquisition and analysis

To calculate the traction forces by which the cells displaced the nanopillars, the deflections of the nanopillar tips were imaged with confocal microscopy using a Leica confocal microscope SP5 with a 63x/1.43 oil immersion objective. During image acquisition, cells were kept at 37 ºC and 10% CO_2 condition. The following laser wavelengths were used to acquire both the images of nanopillar arrays and of the labeled cells: 405 nm (DAPI), 488 nm (to create a DIC image of nanopillar structures, eGFP and FITC) and 546 nm (DiI & TRITC). Each cell was imaged live for 30 to 60 min, with a scanning ratio of 1 min/frame. The displacements in xy direction of the nanopillar tips were quantified by comparison of two images taken at the planes of the pillar tops versus the bottom plane, respectively, using confocal microscopy (image resolution 50 nm per pixel). All nanopillar images were processed by Diatrack 3.03 (Powerful Particle Tracking, Semasopht), Fiji (plugin, template matching for drift collection) and the force calculated according to Hooke's law: $F = k*x$. The average background displacement of pillars was ~15 nm.

Scanning electron microscopy

Cells on nanopillar arrays were imaged with higher magnification using a Scanning Electron Microscope (SEM, Zeiss ULTRA 55). After fixation, the samples were dehydrated by critical point drying using standard procedures. Briefly, this stepwise dehydration procedure started by adding 0.5% aqueous solution of polyethylenimine, followed by dehydrating the samples with a series of ethanol-water washes (25%, 50%, 75%, two 95%, and 100% ethanol) and finishing by drying the sample using critical point drying equipment and CO_2. After dehydration process, the samples were coated with a 5 nm thick layer of gold using a sputter coater.

Cell immunofluorescence

Cells were cultured on nanopillars or 8-well chamber slides (Sigma-Aldrich), cells were fixed with 4% PFA (P6148, Sigma-Aldrich) for 15 min at room temperature (RT). The cell membrane was

then permeabilized with 0.1% Triton X-100 (X100, Sigma-Aldrich) in PBS (10 min) followed by 1% BSA/PBS (85040C, Sigma-Aldrich) blocking step (30 min, RT).

HUVECs and HCMECs were stained with with mouse anti-NCL (1:100, Santa Cruz cat. sc-55486), rabbit anti-PFKFB3 (1:100, abcam cat. ab127894), and rabbit anti-TIGAR (1:200, Sigma cat. HPA040354) in 1% BSA/PBS at 4°C overnight. Cells were the incubated with secondary antibodies goat anti-rabbit Alexa 568 and goat anti-mouse Alexa 488 (1:500, Thermo Fisher Scientific cat. A-11011 and A-11001), or TRITC-labeled phalloidin (1:100 Sigma) in 1% BSA/PBS for 1.5 hours at RT. Nuclei were counterstained with DAPI staining (1:20,000, Thermo Fisher Scientific) in PBS for 5 min.

Bulk RNA-sequencing and analysis

Total RNA was extracted using the RNeasy RNA isolation kit (Qiagen, Hilden, Germany) including a DNase treatment to digest residual genomic DN. RNA sequencing of endothelial cells was performed by the Functional Genomics Center Zurich. Libraries preparation was performed following Illumina TruSeq stranded mRNA protocol. RNA and final libraries quality were addressed using an Agilent 4200 TapeStation System. The libraries were pooled equimolarly and sequenced in an Illumina NovaSeq sequencer (single-end 100 bp) with a depth of around 20 Mio reads per sample. For mapping and trimming of FASTQ format sequences was performed using Trimmomatic v0.3.3, and sequence quality control was assessed using FastQC. Alignment to the Ensembl Homo_sapiens GRCh38.p10 reference genome (Release_91-2018-02-26) was performed using the STAR aligner. Gene expression values were computed with the function featureCounts from the R package Rsubread. Differential expression was computed using the generalized linear model implemented in the Bioconductor package DESeq2. Statistical analysis included Wald test followed by correction for multiple testing using the Benjamini–Hochberg method (False Discovery Rate, FDR). All data is accessible via the accession number GSE224268.

Pathway analysis using Gene Set Enrichment Analysis and cytoscape

Pathway analysis was performed on the ranked differential expression gene list (rank formula:"-*LOG10(pvalue)*SIGN(logFC)*") using the Gene Set Enrichment Analysis (GSEA) software from the Broad Institute (software.broadinstitute.org/GSEA) (version 4.0.1) (76, 77). "Human_GOBP_AllPathways_no_GO_iea_February_01_2022_symbol.gmt" from [http://baderlab.org/GeneSets] was used to identify enriched pathways in GSEA analysis (inclusion criteria are pathways with a minimum size of 15 and a maximum size of 200 genes). The resulting pathways were filtered based on passing the threshold of FDR<0.05 and pvalue<0.05. Significantly regulated pathways were plotted using Cytoscape (Version 3.7.0) and EnrichmentMap (version 3.3) (78). Related pathways were grouped into themes, labeled by AutoAnnotate (version 1.3) and manually curated.

LC-MS/MS analysis polar metabolites

Sample preparation for LC-MS/MS analysis were modified from Paglia et al., 2014 (79) . HCMECs and HUVECs were washed twice with PBS and fixed with 80% methanol. HCMECs and HUVECs were detached using a cell scraper and lyzed mechanically using a dounce homogenizer. Extracts were centrifuged for 20min at 10,000g and 4°C. Samples were stored at -20°C until LC-MS/MS analysis

50 µl methanol extract was dried under a N2, reconstituted in 20 µl water and diluted with 80 µl injection buffer (90% acetonitrile, 8.8% methanol, 50 mM NH4-acetate). Samples were vortexed and centrifuged (10,000g, 4°C, 15 min). 50 µl of the supernatant was transferred to a glass vial (Total Recovery Vials, Waters, Milford, MA, USA) for LC-MS/MS injection.

Metabolites were separated on a nanoAcquity UPLC (Waters, Milford, MA, USA) equipped with a BEH Amide capillary column (150 µm × 130 mm, 1.7 µm particle size, Waters, Milford, MA,

USA). Solvent system consists of buffer A (5 mM NH4-acetate in water) and buffer B (5 mM NH_4-acetate, in 95% acetonitrile). Linear gradient applied from 10% A to 90% A over 10 min, with flow rate ramped down from 3 µl/min to 2 µl/min. Washing for 1 min at 50% A with a flow rate of 2 µl/min, followed by 5 min reequilibration with flow rate ramped to 3 µl/min. Injection volume was 1 µl. The UPLC was coupled to Synapt G2-Si mass spectrometer (Waters, Milford, MA, USA) by a nanoESI source. MS1 and MS2 data was acquired using negative polarization and MSE over a mass range of 50 to 1,200 m/z at resolution of >20,000.

Data were aligned and searched against databases with the Progenesis QI software (Waters, Milford, MA, USA). Polar metabolites and lipids were searched against the KEGG database with a precursor mass tolerance of 20 ppm and fragment mass tolerance of 50 ppm. Quality controls were run on pooled samples and reference compound mixtures to verify technical accuracy and stability. Reference compound included key metabolites from glycolysis, oxidative phosphorylation, TCA cycle, pentose phosphate, as well as amino acids and nucleotides. Those metabolites were manually curated in Skyline.

MetaboAnalyst 5.0 was used The peak intensity (peaks(mz/rt)) table was uploaded as a data matrix file to MetaboAnalyst 5.0 (80) for reading, processing the raw data and for statistical analysis. Statistical analysis performed include univariant analysis (fold change analysis and t-test) to plot the volcano plots. Features that passed the threshold of fold change $-1.5<x<1.5$ and p-value < 0.05 were counted as significantly changed, red dots indicate upregulated, while blue dots indicate down regulated. Multivariant analysis performed include unsupervised principal component analysis (PCA) to plot 2D and 3D PCA plots was done using prcomp package. Clustered heatmaps were plotted using the PlotHeatMap function implemented from the R pheatmap package (distance measure using euclidean, and clustering algorithm using ward.D).

Glycolytic Flux

HCMECs and HUVECs were incubated for 6 hours in EGM-2 containing 0.4 μCi/ml [5-3H]-D-glucose (PerkinElmer). Supernatant was transferred into glass vials containing perchloric acid and sealed with rubber stoppers. $^{3}H_2O$ was captured in hanging wells containing filter paper soaked with H_2O over a period of 48 hours at 37°C (81). Thereafter, the filter paper was transferred in scintillation cocktail for radioactivity measurement by liquid scintillation counting.

Fatty acid oxidation

HCMECs and HUVECs were incubated in FBS-free EGM-2 medium supplemented with 50 fatty-acid free BSA, 50 μM carnitine, 100 μM unlabeled palmitic acid, and 2 μCi/ml [9,10-^{3}H]-palmitic acid. Again, supernatant was transferred into glass vials and sealed with rubber stoppers. Radioactivity was measured as in the glycolytic flux assay.

Glucose uptake and lactate production

Glucose and lactate concentrations in HCMECs and HUVECs supernatant as well as in basal growth medium were measured at the Toronto General Hospital and the University Hospital of Zurich.

Glucose uptake was calculated by subtracting glucose concentration in medium by the concentration in cell supernatant. Lactate production was calculated by subtracting lactate concentration in medium by the concentration in cell supernatant.

Spatial Transcriptomics - Visualization of MHC class II gene expression on 10X Genomics Visium data

To examine the spatial gene expression of Nucleolin, we analyzed the publicly available Visium data for human glioblastoma (https://www.10xgenomics.com/resources/datasets/human-glioblastoma-whole-transcriptome-analysis-1-standard-1-2-0) using the Seurat v4 workflow. In

brief, data was sc-transformed using the "SCTransform" and spatial expression plots were generated using "SpatialFeaturePlot" functions.

Statistical analysis

Statistical significance was determined using unpaired two-tailed Student's t-test, one-way or two-way ANOVAs (GraphPad Prism8). Differences were considered significant with a P value less than 0.05. Quantified data are presented as mean ± SEM.

SUPPLEMENTARY REFERENCES

1. Singh SK, Hawkins C, Clarke ID, Squire JA, Bayani J, Hide T, et al. Identification of human brain tumour initiating cells. *Nature.* 2004;432(7015):396-401.
2. Chen J, Li Y, Yu TS, McKay RM, Burns DK, Kernie SG, et al. A restricted cell population propagates glioblastoma growth after chemotherapy. *Nature.* 2012;488(7412):522-6.
3. Thakkar JP, Dolecek TA, Horbinski C, Ostrom QT, Lightner DD, Barnholtz-Sloan JS, et al. Epidemiologic and molecular prognostic review of glioblastoma. *Cancer Epidemiol Biomarkers Prev.* 2014;23(10):1985-96.
4. Gilbert MR, Wang M, Aldape KD, Stupp R, Hegi ME, Jaeckle KA, et al. Dose-dense temozolomide for newly diagnosed glioblastoma: a randomized phase III clinical trial. *J Clin Oncol.* 2013;31(32):4085-91.
5. Stupp R, Mason WP, van den Bent MJ, Weller M, Fisher B, Taphoorn MJ, et al. Radiotherapy plus concomitant and adjuvant temozolomide for glioblastoma. *N Engl J Med.* 2005;352(10):987-96.
6. Phng LK, and Gerhardt H. Angiogenesis: a team effort coordinated by notch. *Dev Cell.* 2009;16(2):196-208.
7. Gerhardt H, Golding M, Fruttiger M, Ruhrberg C, Lundkvist A, Abramsson A, et al. VEGF guides angiogenic sprouting utilizing endothelial tip cell filopodia. *J Cell Biol.* 2003;161(6):1163-77.
8. Potente M, Gerhardt H, and Carmeliet P. Basic and therapeutic aspects of angiogenesis. *Cell.* 2011;146(6):873-87.
9. Hjelmeland AB, Lathia JD, Sathornsumetee S, and Rich JN. Twisted tango: brain tumor neurovascular interactions. *Nat Neurosci.* 2011;14(11):1375-81.
10. Jayson GC, Kerbel R, Ellis LM, and Harris AL. Antiangiogenic therapy in oncology: current status and future directions. *Lancet.* 2016;388(10043):518-29.
11. Weis SM, and Cheresh DA. Tumor angiogenesis: molecular pathways and therapeutic targets. *Nat Med.* 2011;17(11):1359-70.
12. Walchli T, Wacker A, Frei K, Regli L, Schwab ME, Hoerstrup SP, et al. Wiring the Vascular Network with Neural Cues: A CNS Perspective. *Neuron.* 2015;87(2):271-96.
13. Carmeliet P, and Jain RK. Molecular mechanisms and clinical applications of angiogenesis. *Nature.* 2011;473(7347):298-307.
14. Jain RK, and Carmeliet P. SnapShot: Tumor angiogenesis. *Cell.* 2012;149(6):1408- e1.
15. Hellstrom M, Phng LK, and Gerhardt H. VEGF and Notch signaling: the yin and yang of angiogenic sprouting. *Cell Adh Migr.* 2007;1(3):133-6.
16. Jakobsson L, Franco CA, Bentley K, Collins RT, Ponsioen B, Aspalter IM, et al. Endothelial cells dynamically compete for the tip cell position during angiogenic sprouting. *Nat Cell Biol.* 2010;12(10):943-53.
17. Ghajar CM, Peinado H, Mori H, Matei IR, Evason KJ, Brazier H, et al. The perivascular niche regulates breast tumour dormancy. *Nat Cell Biol.* 2013;15(7):807-17.
18. Carlson P, Dasgupta A, Grzelak CA, Kim J, Barrett A, Coleman IM, et al. Targeting the perivascular niche sensitizes disseminated tumour cells to chemotherapy. *Nat Cell Biol.* 2019;21(2):238-50.
19. Wolfson E, Goldenberg M, Solomon S, Frishberg A, and Pinkas-Kramarski R. Nucleolin-binding by ErbB2 enhances tumorigenicity of ErbB2-positive breast cancer. *Oncotarget.* 2016;7(40):65320-34.
20. Wolfson E, Solomon S, Schmukler E, Goldshmit Y, and Pinkas-Kramarski R. Nucleolin and ErbB2 inhibition reduces tumorigenicity of ErbB2-positive breast cancer. *Cell Death Dis.* 2018;9(2):47.

21. Huang F, Wu Y, Tan H, Guo T, Zhang K, Li D, et al. Phosphorylation of nucleolin is indispensable to its involvement in the proliferation and migration of non-small cell lung cancer cells. *Oncol Rep.* 2019;41(1):590-8.
22. Xu Z, Joshi N, Agarwal A, Dahiya S, Bittner P, Smith E, et al. Knocking down nucleolin expression in gliomas inhibits tumor growth and induces cell cycle arrest. *J Neurooncol.* 2012;108(1):59-67.
23. Ishimaru D, Zuraw L, Ramalingam S, Sengupta TK, Bandyopadhyay S, Reuben A, et al. Mechanism of regulation of bcl-2 mRNA by nucleolin and A+U-rich element-binding factor 1 (AUF1). *J Biol Chem.* 2010;285(35):27182-91.
24. Otake Y, Soundararajan S, Sengupta TK, Kio EA, Smith JC, Pineda-Roman M, et al. Overexpression of nucleolin in chronic lymphocytic leukemia cells induces stabilization of bcl2 mRNA. *Blood.* 2007;109(7):3069-75.
25. Takagi M, Absalon MJ, McLure KG, and Kastan MB. Regulation of p53 translation and induction after DNA damage by ribosomal protein L26 and nucleolin. *Cell.* 2005;123(1):49-63.
26. Balca-Silva J, do Carmo A, Tao H, Rebelo O, Barbosa M, Moura-Neto V, et al. Nucleolin is expressed in patient-derived samples and glioblastoma cells, enabling improved intracellular drug delivery and cytotoxicity. *Exp Cell Res.* 2018;370(1):68-77.
27. Galzio R, Rosati F, Benedetti E, Cristiano L, Aldi S, Mei S, et al. Glycosilated nucleolin as marker for human gliomas. *Journal of cellular biochemistry.* 2012;113(2):571-9.
28. Srivastava M, and Pollard HB. Molecular dissection of nucleolin's role in growth and cell proliferation: new insights. *Faseb J.* 1999;13(14):1911-22.
29. Tuteja R, and Tuteja N. Nucleolin: a multifunctional major nucleolar phosphoprotein. *Crit Rev Biochem Mol Biol.* 1998;33(6):407-36.
30. Bensaad K, Tsuruta A, Selak MA, Vidal MN, Nakano K, Bartrons R, et al. TIGAR, a p53-inducible regulator of glycolysis and apoptosis. *Cell.* 2006;126(1):107-20.
31. Ko YH, Domingo-Vidal M, Roche M, Lin Z, Whitaker-Menezes D, Seifert E, et al. TP53-inducible Glycolysis and Apoptosis Regulator (TIGAR) Metabolically Reprograms Carcinoma and Stromal Cells in Breast Cancer. *J Biol Chem.* 2016;291(51):26291-303.
32. Das S, and Marsden PA. Angiogenesis in glioblastoma. *N Engl J Med.* 2013;369(16):1561-3.
33. Weller M, Wick W, Aldape K, Brada M, Berger M, Pfister SM, et al. Glioma. *Nat Rev Dis Primers.* 2015;1:15017.
34. Arvanitis CD, Ferraro GB, and Jain RK. The blood-brain barrier and blood-tumour barrier in brain tumours and metastases. *Nat Rev Cancer.* 2020;20(1):26-41.
35. Steeg PS. The blood-tumour barrier in cancer biology and therapy. *Nat Rev Clin Oncol.* 2021;18(11):696-714.
36. Aldape K, Brindle KM, Chesler L, Chopra R, Gajjar A, Gilbert MR, et al. Challenges to curing primary brain tumours. *Nat Rev Clin Oncol.* 2019;16(8):509-20.
37. Quail DF, and Joyce JA. The Microenvironmental Landscape of Brain Tumors. *Cancer cell.* 2017;31(3):326-41.
38. Quail DF, and Joyce JA. Microenvironmental regulation of tumor progression and metastasis. *Nat Med.* 2013;19(11):1423-37.
39. Griveau A, Seano G, Shelton SJ, Kupp R, Jahangiri A, Obernier K, et al. A Glial Signature and Wnt7 Signaling Regulate Glioma-Vascular Interactions and Tumor Microenvironment. *Cancer cell.* 2018;33(5):874-89 e7.
40. Huijbers EJM, Khan KA, Kerbel RS, and Griffioen AW. Tumors resurrect an embryonic vascular program to escape immunity. *Sci Immunol.* 2022;7(67):eabm6388.

41. Fogal V, Sugahara KN, Ruoslahti E, and Christian S. Cell surface nucleolin antagonist causes endothelial cell apoptosis and normalization of tumor vasculature. *Angiogenesis.* 2009;12(1):91-100.
42. Romano S, Moura V, Simoes S, Moreira JN, and Goncalves J. Anticancer activity and antibody-dependent cell-mediated cytotoxicity of novel anti-nucleolin antibodies. *Sci Rep.* 2018;8(1):7450.
43. Reyes-Reyes EM, and Akiyama SK. Cell-surface nucleolin is a signal transducing P-selectin binding protein for human colon carcinoma cells. *Exp Cell Res.* 2008;314(11-12):2212-23.
44. Sampson JH, Gunn MD, Fecci PE, and Ashley DM. Brain immunology and immunotherapy in brain tumours. *Nat Rev Cancer.* 2020;20(1):12-25.
45. Ginisty H, Sicard H, Roger B, and Bouvet P. Structure and functions of nucleolin. *J Cell Sci.* 1999;112 (Pt 6):761-72.
46. Mongelard F, and Bouvet P. Nucleolin: a multiFACeTed protein. *Trends Cell Biol.* 2007;17(2):80-6.
47. Chen Z, and Xu X. Roles of nucleolin. Focus on cancer and anti-cancer therapy. *Saudi Med J.* 2016;37(12):1312-8.
48. Jia W, Yao Z, Zhao J, Guan Q, and Gao L. New perspectives of physiological and pathological functions of nucleolin (NCL). *Life Sci.* 2017;186:1-10.
49. Wälchli T, Bisschop J, Carmeliet P, Zadeh G, Monnier PP, De Bock K, et al. Shaping the brain vasculature in development and disease in the single-cell era. *Nat Rev Neurosci.* 2022.
50. Cruys B, Wong BW, Kuchnio A, Verdegem D, Cantelmo AR, Conradi LC, et al. Glycolytic regulation of cell rearrangement in angiogenesis. *Nat Commun.* 2016;7:12240.
51. Boas SE, and Merks RM. Tip cell overtaking occurs as a side effect of sprouting in computational models of angiogenesis. *BMC Syst Biol.* 2015;9:86.
52. Cao J, Ehling M, Marz S, Seebach J, Tarbashevich K, Sixta T, et al. Polarized actin and VE-cadherin dynamics regulate junctional remodelling and cell migration during sprouting angiogenesis. *Nat Commun.* 2017;8(1):2210.
53. Walchli T, Mateos JM, Weinman O, Babic D, Regli L, Hoerstrup SP, et al. Quantitative assessment of angiogenesis, perfused blood vessels and endothelial tip cells in the postnatal mouse brain. *Nature protocols.* 2015;10(1):53-74.
54. De Smet F, Segura I, De Bock K, Hohensinner PJ, and Carmeliet P. Mechanisms of vessel branching: filopodia on endothelial tip cells lead the way. *Arterioscler Thromb Vasc Biol.* 2009;29(5):639-49.
55. Carmeliet P, De Smet F, Loges S, and Mazzone M. Branching morphogenesis and antiangiogenesis candidates: tip cells lead the way. *Nat Rev Clin Oncol.* 2009;6(6):315-26.
56. Kim YH, Choi J, Yang MJ, Hong SP, Lee CK, Kubota Y, et al. A MST1-FOXO1 cascade establishes endothelial tip cell polarity and facilitates sprouting angiogenesis. *Nat Commun.* 2019;10(1):838.
57. Lizama CO, and Zovein AC. Polarizing pathways: balancing endothelial polarity, permeability, and lumen formation. *Exp Cell Res.* 2013;319(9):1247-54.
58. Xu ZP, Tsuji T, Riordan JF, and Hu GF. The nuclear function of angiogenin in endothelial cells is related to rRNA production. *Biochem Biophys Res Commun.* 2002;294(2):287-92.
59. Shiu JY, Aires L, Lin Z, and Vogel V. Nanopillar force measurements reveal actin-cap-mediated YAP mechanotransduction. *Nat Cell Biol.* 2018;20(3):262-71.
60. De Bock K, Georgiadou M, Schoors S, Kuchnio A, Wong BW, Cantelmo AR, et al. Role of PFKFB3-driven glycolysis in vessel sprouting. *Cell.* 2013;154(3):651-63.
61. Herbert SP, and Stainier DY. Molecular control of endothelial cell behaviour during blood vessel morphogenesis. *Nat Rev Mol Cell Biol.* 2011;12(9):551-64.

62. Apte RS, Chen DS, and Ferrara N. VEGF in Signaling and Disease: Beyond Discovery and Development. *Cell.* 2019;176(6):1248-64.
63. Rahimi N, and Costello CE. Emerging roles of post-translational modifications in signal transduction and angiogenesis. *Proteomics.* 2015;15(2-3):300-9.
64. Arcondeguy T, Lacazette E, Millevoi S, Prats H, and Touriol C. VEGF-A mRNA processing, stability and translation: a paradigm for intricate regulation of gene expression at the post-transcriptional level. *Nucleic Acids Res.* 2013;41(17):7997-8010.
65. Mancuso MR, Kuhnert F, and Kuo CJ. Developmental angiogenesis of the central nervous system. *Lymphat Res Biol.* 2008;6(3-4):173-80.
66. Blanco R, and Gerhardt H. VEGF and notch in tip and stalk cell selection. *Cold Spring Harb Perspect Med.* 2013;3(1).
67. Geudens I, and Gerhardt H. Coordinating cell behaviour during blood vessel formation. *Development.* 2011;138(21):4569-83.
68. Jain RK, di Tomaso E, Duda DG, Loeffler JS, Sorensen AG, and Batchelor TT. Angiogenesis in brain tumours. *Nat Rev Neurosci.* 2007;8(8):610-22.
69. Dhez AC, Benedetti E, Antonosante A, Panella G, Ranieri B, Florio TM, et al. Targeted therapy of human glioblastoma via delivery of a toxin through a peptide directed to cell surface nucleolin. *J Cell Physiol.* 2018;233(5):4091-105.
70. Cheung HC, Hai T, Zhu W, Baggerly KA, Tsavachidis S, Krahe R, et al. Splicing factors PTBP1 and PTBP2 promote proliferation and migration of glioma cell lines. *Brain.* 2009;132(Pt 8):2277-88.
71. Louis DN, Perry A, Reifenberger G, von Deimling A, Figarella-Branger D, Cavenee WK, et al. The 2016 World Health Organization Classification of Tumors of the Central Nervous System: a summary. *Acta Neuropathol.* 2016;131(6):803-20.
72. Perry A, and Wesseling P. Histologic classification of gliomas. *Handb Clin Neurol.* 2016;134:71-95.
73. Rodak R, Kubota H, Ishihara H, Eugster HP, Konu D, Mohler H, et al. Induction of reactive oxygen intermediates-dependent programmed cell death in human malignant ex vivo glioma cells and inhibition of the vascular endothelial growth factor production by taurolidine. *J Neurosurg.* 2005;102(6):1055-68.
74. Korff T, Krauss T, and Augustin HG. Three-dimensional spheroidal culture of cytotrophoblast cells mimics the phenotype and differentiation of cytotrophoblasts from normal and preeclamptic pregnancies. *Exp Cell Res.* 2004;297(2):415-23.
75. Kuo CW, Shiu JY, Chien FC, Tsai SM, Chueh DY, and Chen P. Polymeric nanopillar arrays for cell traction force measurements. *Electrophoresis.* 2010;31(18):3152-8.
76. Subramanian A, Tamayo P, Mootha VK, Mukherjee S, Ebert BL, Gillette MA, et al. Gene set enrichment analysis: A knowledge-based approach for interpreting genome-wide expression profiles. *Proceedings of the National Academy of Sciences.* 2005;102(43):15545.
77. Reimand J, Isserlin R, Voisin V, Kucera M, Tannus-Lopes C, Rostamianfar A, et al. Pathway enrichment analysis and visualization of omics data using g:Profiler, GSEA, Cytoscape and EnrichmentMap. *Nature Protocols.* 2019;14(2):482-517.
78. Shannon P, Markiel A, Ozier O, Baliga NS, Wang JT, Ramage D, et al. Cytoscape: a software environment for integrated models of biomolecular interaction networks. *Genome Res.* 2003;13(11):2498-504.
79. Paglia G, Williams JP, Menikarachchi L, Thompson JW, Tyldesley-Worster R, Halldorsson S, et al. Ion mobility derived collision cross sections to support metabolomics applications. *Anal Chem.* 2014;86(8):3985-93.

80. Pang Z, Zhou G, Ewald J, Chang L, Hacariz O, Basu N, et al. Using MetaboAnalyst 5.0 for LC-HRMS spectra processing, multi-omics integration and covariate adjustment of global metabolomics data. *Nature protocols.* 2022;17(8):1735-61.
81. Aragones J, Schneider M, Van Geyte K, Fraisl P, Dresselaers T, Mazzone M, et al. Deficiency or inhibition of oxygen sensor Phd1 induces hypoxia tolerance by reprogramming basal metabolism. *Nature genetics.* 2008;40(2):170-80.

SUPPLEMENTARY FIGURES

Supplementary Figure 1

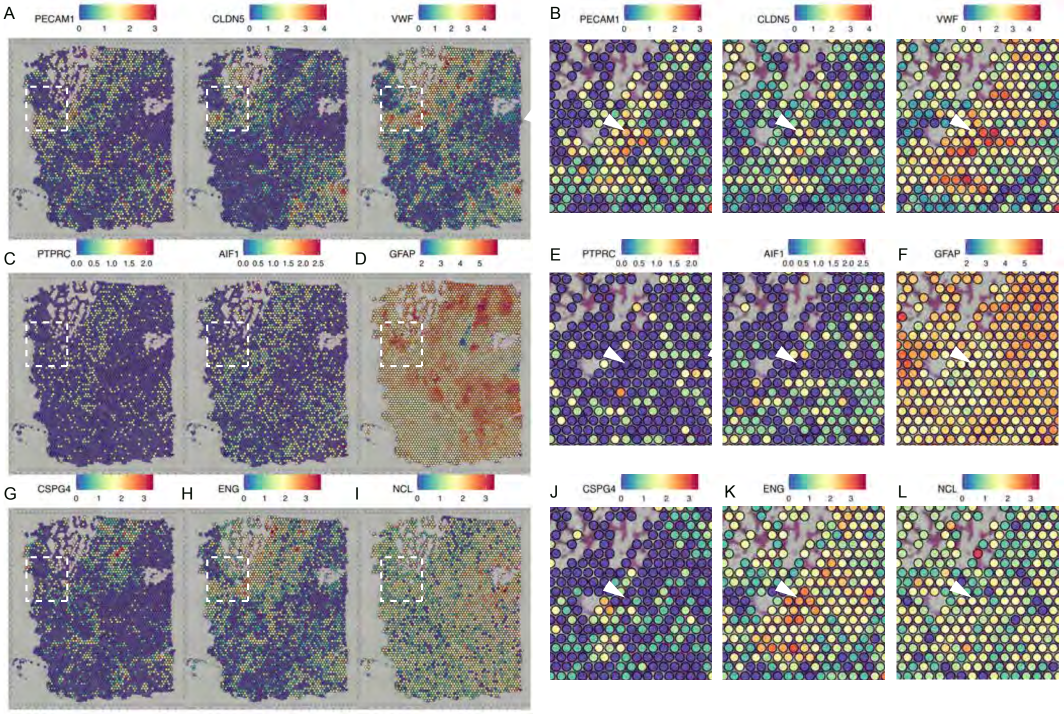

Supplementary Figure 1 Spatial transcriptomics reveals expression of Nucleolin in endothelial and perivascular cells in human glioblastoma in vivo

A-L Spatial expression of endothelial markers (**A,B**; *PECAM1*, *CLDN5*, *VWF*), microglia/macrophage markers (**C,E**; *PTPRC*, *AIF1*), astrocyte marker (**D,F**; *GFAP*), pericyte marker (**G,J**; *CSPG4*), activated endothelial cell markers (**H,K**; *ENG*) and Nucleolin (**I,L**; *NCL*) in publicly available 10X Genomics Visium sections of human glioblastoma. Nucleolin is expressed in (activated) endothelial cells (**A**,**B**,**G**,**H**,**I**) and in the perivascular cells microglia/macrophages (**C**,**E**,**I**), astrocytes (**D**,**F**,**I**) and pericytes (**G**,**J**,**I**) in human glioblastoma. The boxed areas (white dotted box) in **A**,**C**,**D**,**G**,**H**,**I** are zoomed in **B**,**E**,**F**,**J**,**K**,**L** respectively.

Supplementary Figure 2

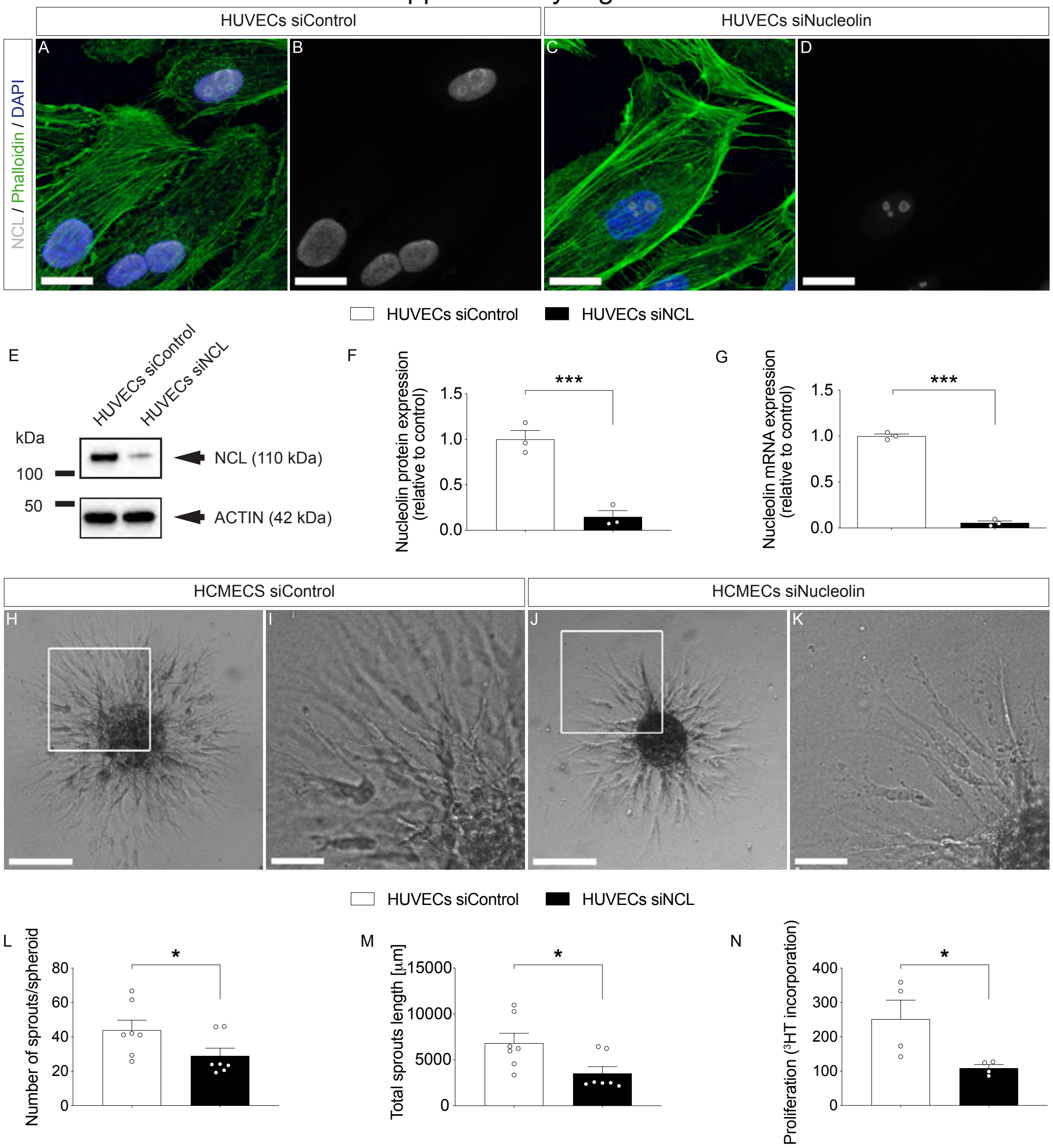

Supplementary Figure 2 Nucleolin promotes peripheral vascular endothelial cell sprouting and proliferation in vitro

A-D HUVECs were stained for Nucleolin (green), F-actin (red, stained with Phalloidin), and the general nuclear marker DAPI (blue). Nucleolin expression was decreased and restricted to nucleoli upon siRNA-mediated knockdown in HUVECs (siNCL, **C**,**D**) as compared to control-siRNA treated HUVECs (**A**,**B**).

E Western blot showing Nucleolin downregulation in HUVECs transfected with siRNA against Nucleolin as compared to control-siRNA treated HUVECs (n=3). (n=3).

F Quantification of Western blot revealing a significant downregulation of 80% Nucleolin protein expression by siRNA-targeted Nucleolin knock-down as compared to control cells (n=3).

G Quantitative RT-PCR revealing a significant downregulation of more than 90% of Nucleolin mRNA expression by siRNA-targeted Nucleolin knock-down (n=3).

H-**K** HUVEC sprout formation was decreased upon siRNA-mediated Nucleolin knockdown (**J**,**K**) as compared to the control group (**H**,**I**). The boxed areas in **H**,**J** are enlarged in **I**,**K** respectively.

L,**M** HUVEC sprout formation and total sprout length were significantly reduced upon Nucleolin knock-down as compared to the control group (**L**,**M**) (n=3).

N HUVEC proliferation was significantly decreased upon Nucleolin knock-down, as revealed by reduction of radioactively labeled Thymidine incorporation (n=3).

Data represent mean ± SEM. For statistical analysis, two-tailed unpaired Student's t -test (**F**-**G**, **L**-**N**) and two-way ANOVA with Tukey's multiple comparison test with Tukey's multiple-comparison test comparing treatment columns(o), were performed. *$P < 0.05$, **$P < 0.01$, ***$P < 0.001$, ****$P < 0.0001$. Scale bars: 20 µm in **A**-**D**; 150 µm in **H**,**J**; and 50 µm in **I**-**K**.

Supplementary Figure 3

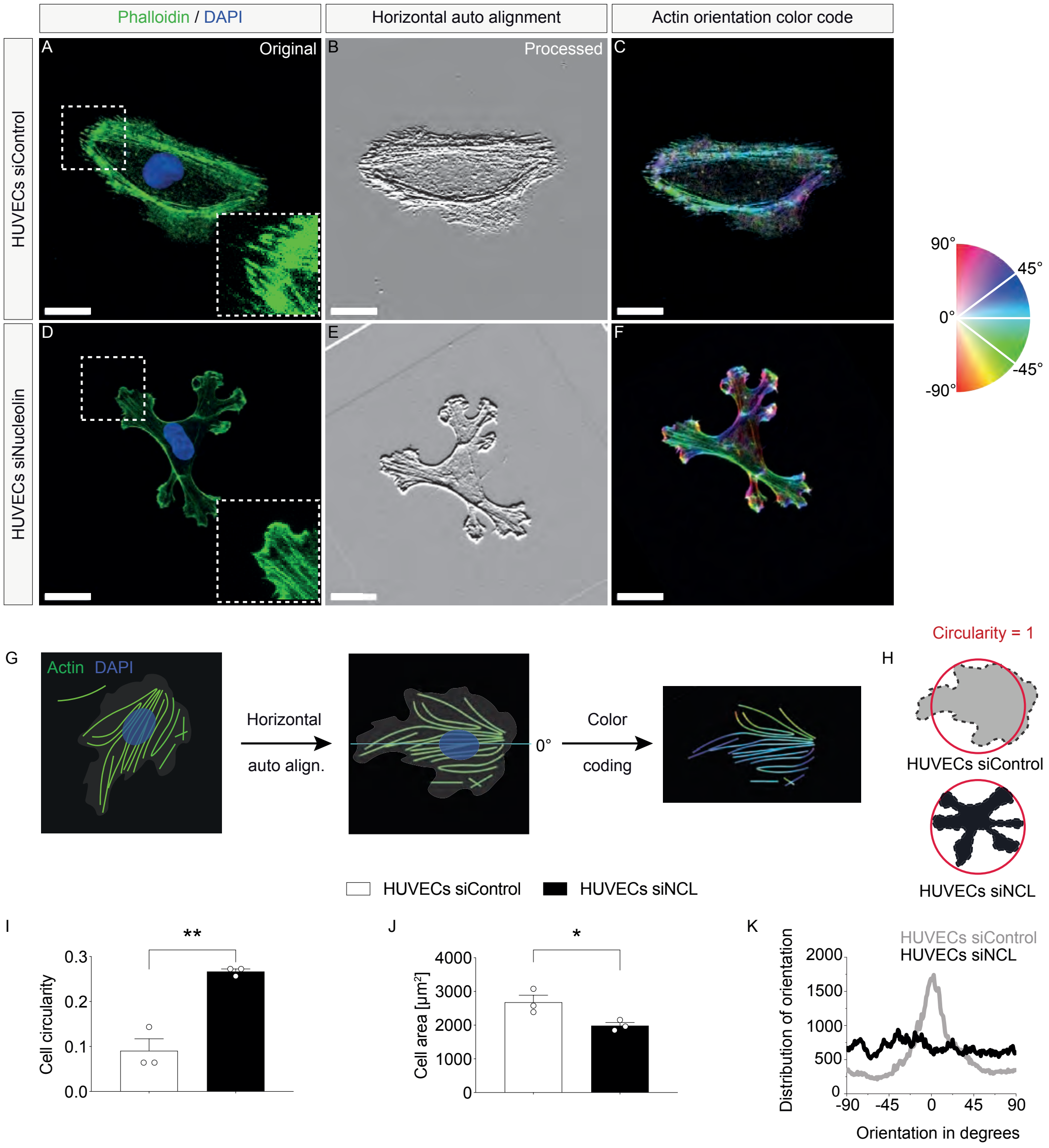

Supplementary Figure 3 Nucleolin affects HUVEC actin cytoskeleton orientation in vitro

A-**F** HUVEC treated with control- or Nucleolin siRNA were left to spread on fibronectin-coated glass substrate and stained for F-actin (green) and DAPI (blue) (**A**,**D**).

G Scheme illustrating actin fiber orientation characterization.

H Schematic illustration of circularity index indicating the reference circular index (circle = 1).

I,**J** Nucleolin knockdown decreased HUVEC cell spreading, as measured by cell circularity and cell area measurements. Nucleolin knock-down HUVECs had a significantly less elongated shape(**I**).

HUVEC spreading was significantly decreased upon Nucleolin knockdown (**J**). Phalloidin actin fibers (green) were more randomly organized in the HUVEC$^{Nucleolin\ KD}$ (**E**,**F**) as compared to the control (**B**,**C**). The distribution of actin orientation shows clear classical peak close to 0 degree in the control HUVEC$^{Control\ KD}$ (grey curve). In HUVEC$^{Nucleolin\ KD}$, the classical peak of actin orientation was lost and HUVEC actin orientation was more randomly distributed (black curve) (**K**).

Data represent mean ± SEM of 3 independent experiments. For statistical analysis, two-tailed unpaired Student's t -test were performed. *$P < 0.05$, **$P < 0.01$, ***$P < 0.001$. Scale bars: 20 µm in **A**-**F**.

Supplementary Figure 4

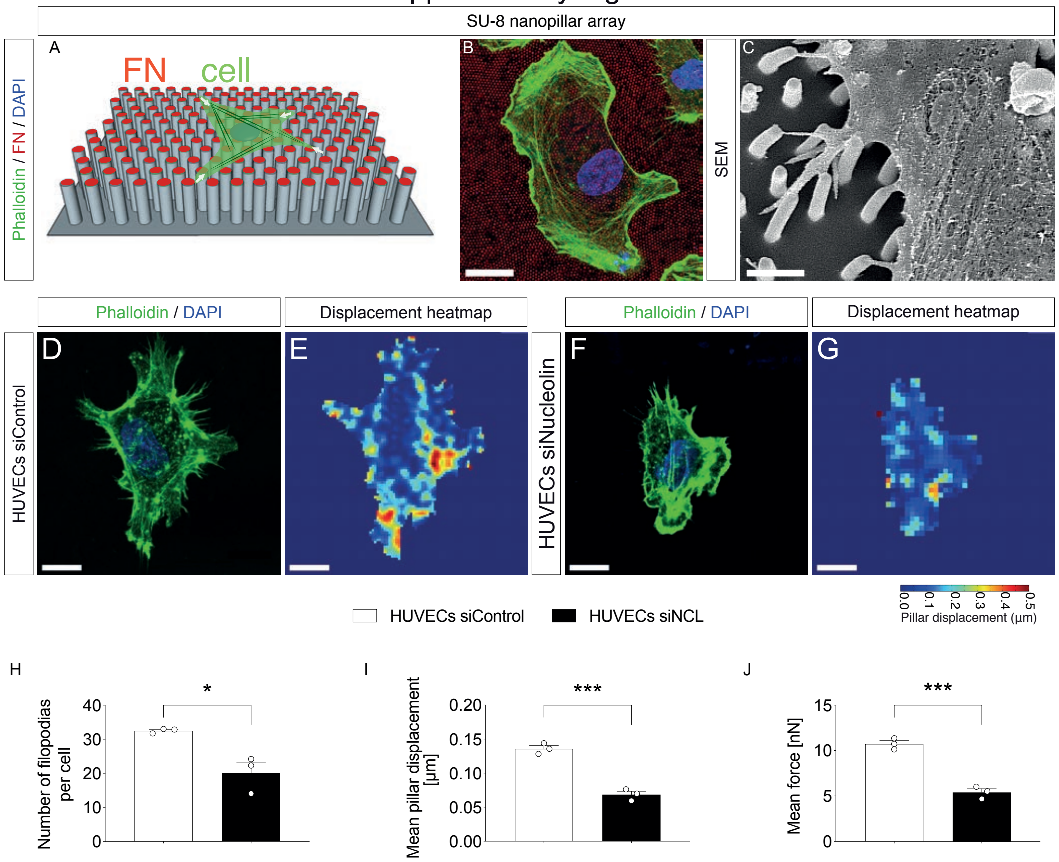

Supplementary Figure 4 Nucleolin regulates HUVEC lamellipodia and filopodia in vitro

A-C Scheme illustrating a spread endothelial cell (green) on a SU-8 nanopillar array (grey) coated with fibronectin (red) (**A**). F-actin (green) and DAPI (blue) stained HUVEC on a fibronectin-coated nanopillar substrate (red) (**B**). Scanning electron microscopy (SEM) image of HUVEC filopodia attaching to nanopillars (**C**). Note the nanopillar-deflection caused by retracting HUVEC filopodia (arrowheads), allowing to optically measure the displacement of the nanopillar and the induced corresponding traction forces.

D-J HUVECs treated with siRNA (for Nucleolin, and control) were placed on nanopillar substrate, and were stained for F-actin (green) and the general nuclear marker DAPI (blue) (**O**,**Q**). The number of filopodia per cell was decreased significantly in siRNA-mediated Nucleolin knockdown in comparison with control siRNA-treated HUVECs (**S**). Explorative movements of HUVECs (and its lamellipodia- and filopodia extensions) were reduced upon Nucleolin knockdown as evidenced by displacement heatmaps (**P**,**R**). Nucleolin downregulation decreased mean nanopillar displacement (**T**) and mean filopodia force (**U**) accordingly.

Data represent mean ± SEM of 3 independent experiments. For statistical analysis, two-tailed unpaired Student's t -test were performed. *$P < 0.05$, **$P < 0.01$, ***$P < 0.001$. Scale bars:10 µm in **B,D-G**; and 2 µm in **C**.

Supplementary Figure 5

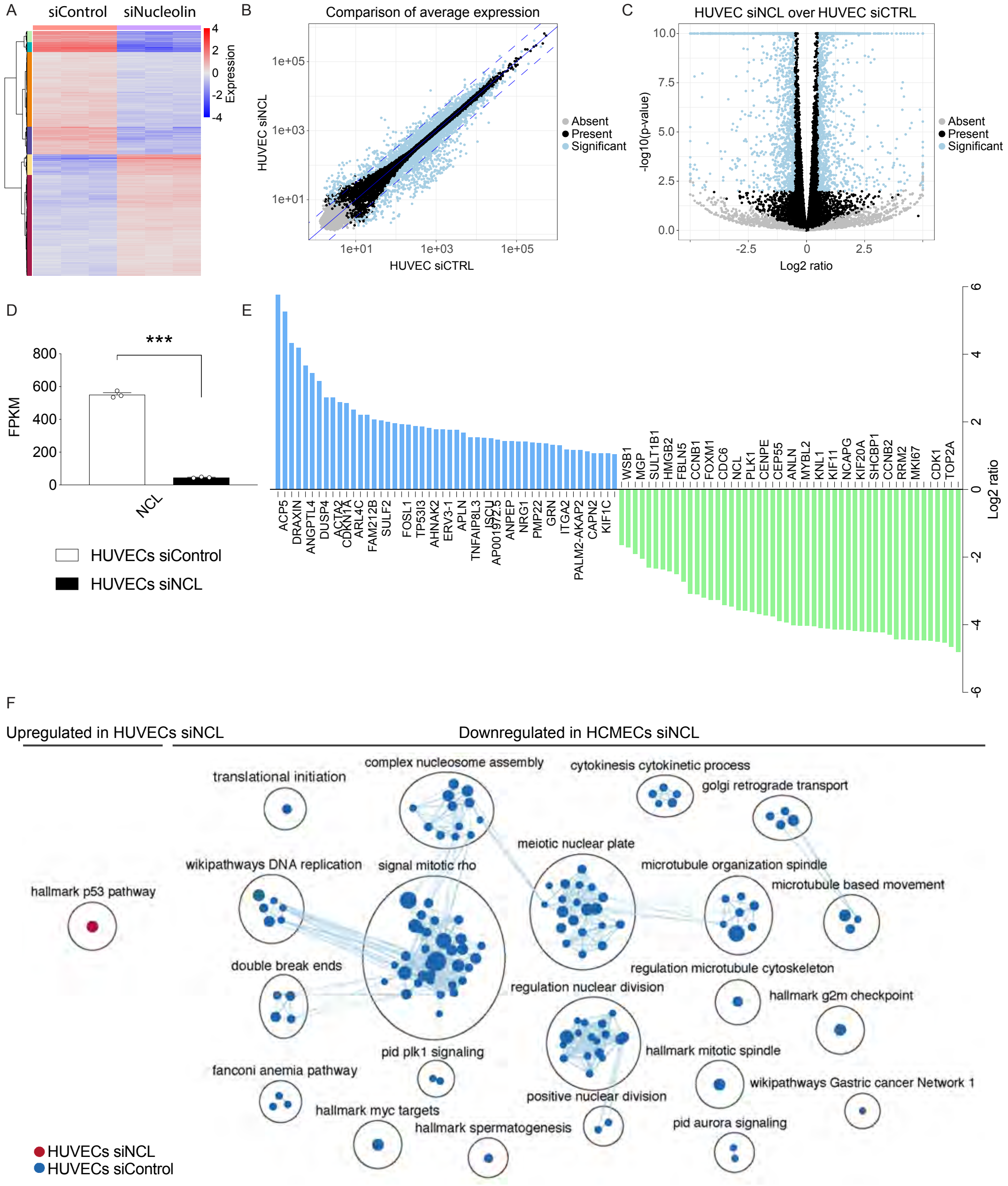

Supplementary Figure 5: Nucleolin induces regulation of angiogenic pathways including Dll4-Jagged-Notch-Hey-Hes-, YAP-TAZ-CTGF-Cyr61-, VEGF-A-VEGFR2 and endothelial glucose metabolism in HUVECs

Transcriptome analysis via RNA sequencing of HUVECs treated with small interfering RNA (siRNA) against Nucleolin (siNCL) and control siRNA (siControl) in three independent experiments.

A Heatmap and hierarchical clustering of siNucleolin treated HUVECs as compared to siControl treated HUVECs.

B-**C** 3240 genes were differentially regulated between HUVECs$^{\text{Nucleolin KD}}$ and HUVECs$^{\text{Control KD}}$ and indicated in blue on scatter (**b**) and volcano plots (**c**).

D Top 50 significantly up (blue)- or down (green)-regulated genes detected by RNA-seq in HUVECs upon Nucleolin knock-down as compared to control treatment. Differentially regulated genes were arranged according to fold change of gene expression.

E Nucleolin gene expression (FPKM) was significantly downregulated by siNucleolin treatment.

F Gene set enrichment analysis (GSEA) and cytoscape enrichment map showed a significant up-regulation of P53 signaling pathway in HUVECS treated with siRNA against Nucleolin. Whereas pathways involved in cell proliferation such as cell cycle regulation and mitosis were enriched in the control treatment. Pathways enriched in Nucleolin KD HUVECs are labeled in red and pathways enriched in control HUVECs are labeled in blue. Pathways are indicated by colored nodes. Their size represents the number of genes they contain. Green lines indicate relationships between the pathways. Black circles group related pathways.

Data represent mean ± SEM. For statistical analysis, Wald tests corrected for multiple testing using the Benjamini and Hochberg method were performed.

Supplementary Figure 6

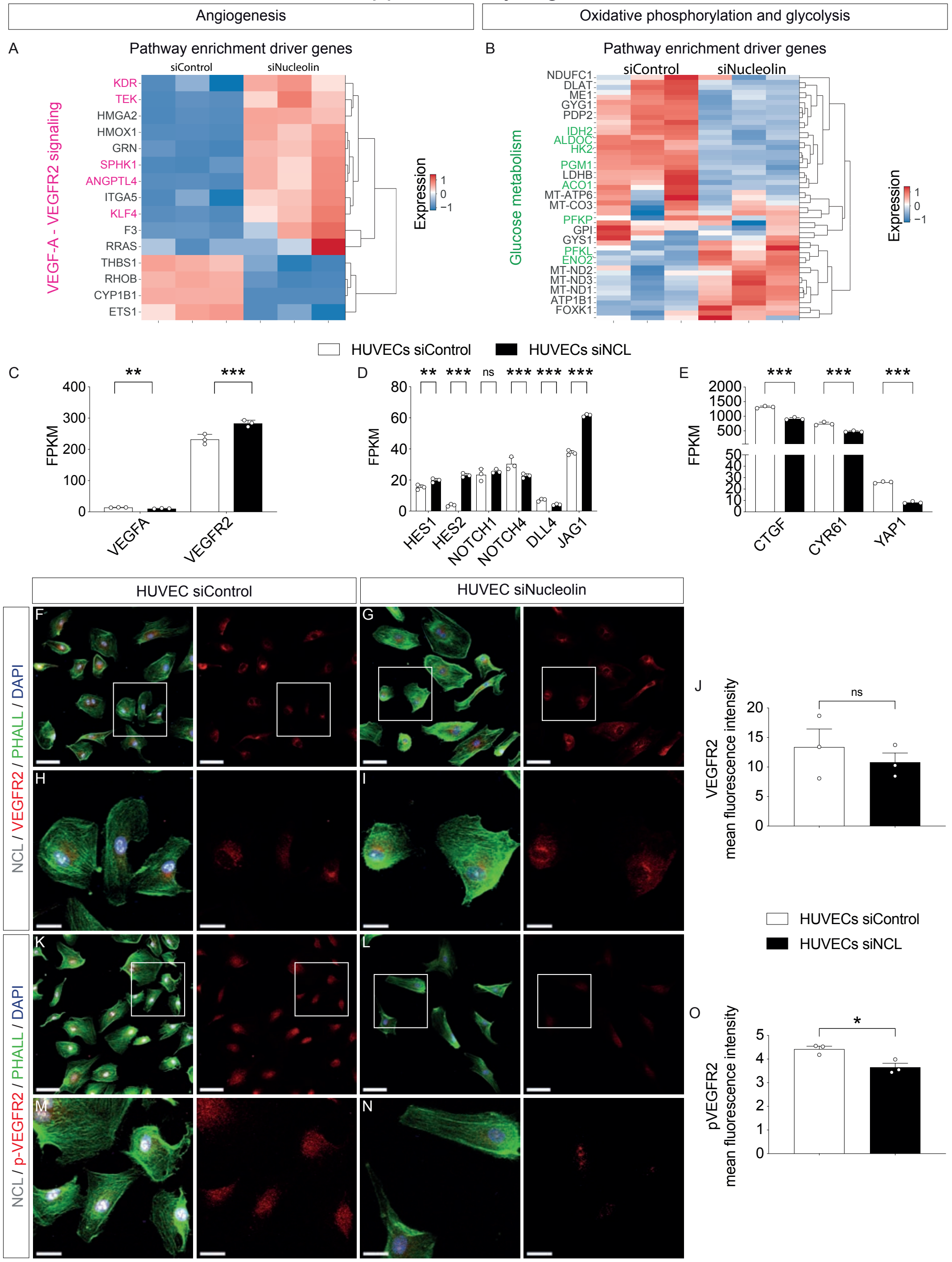

Supplementary Figure 6: Nucleolin induces regulation of angiogenic and promotes phosphorylation of VEGFR2 in HUVECs

A,B Heatmap of the expression of the top 15 genes driving the enrichment of "regulation of angiogenesis" pathways in $HCMEC^{Nucleolin\ KD}$ and the top 50 genes driving the enrichment of "Oxidative phosphorylation and glycolysis" pathways in $HCMEC^{Control\ KD}$ showed different expression pattern in HUVECs as compared to HCMECs. However, key driver genes of the VEGF-VEGFR2 signaling pathway such as VEGFR2 (*KDR*), Tie-2 (*TEK*), *KLF4* and glycolytic genes such as *HK2* and *ALDOC* showed similar regulation as presented in HCMECs.

C-**E** *VEGFA* expression was downregulated and *VEGFR2* expression upregulated (**C**) in $HUVECs^{Nucleolin\ KD}$ as compared to $HUVECs^{Control\ KD}$. Nucleolin knock-down induced a significant upregulation of the Dll4-Jagged-Notch signaling pathway including: *HES1*, *HES2*, Jagged1 (*JAG1*, **D**). *NOTCH4*, and *DLL4* were downregulated upon Nucleolin knock-down (**D**). siNucleolin treatment caused a significant down-regulation of the YAP-TAZ gene *YAP1* as well as the YAP-TAZ downstream effectors genes *CTGF* and *CYR61* (**D**).

F-**O** HUVECs were stained for Nucleolin (gray), F-actin (stained with Phalloidin, green), VEGFR2 (red, in **F-I**) or phopho-VEGFR2 (red in **K**-**N**) and the general nuclear marker DAPI (blue). VEGFR2 expression was not regulated (**J**) and phospho-VEGFR2 expression was significantly downregulated (**O**) upon siRNA-mediated Nucleolin knockdown in HUVECs as compared to control condition. Data represent mean ± SEM. For statistical analysis, Wald test corrected for multiple testing using the Benjamini and Hochberg method (**C**-**E**) and two-tailed unpaired Student's t -test (**P**,**U**) were performed. *$P < 0.05$, **$P < 0.01$, ***$P < 0.001$, ****$P < 0.0001$. The boxed areas (white box) in **F**,**G**,**K**,**L** are zoomed in **H**,**I**,**M**,**N** respectively. Scale bars: 70 µm in **F**,**G**,**K**,**L** and 20 µm in **H**,**I**,**M**,**N**.

Supplementary Figure 7

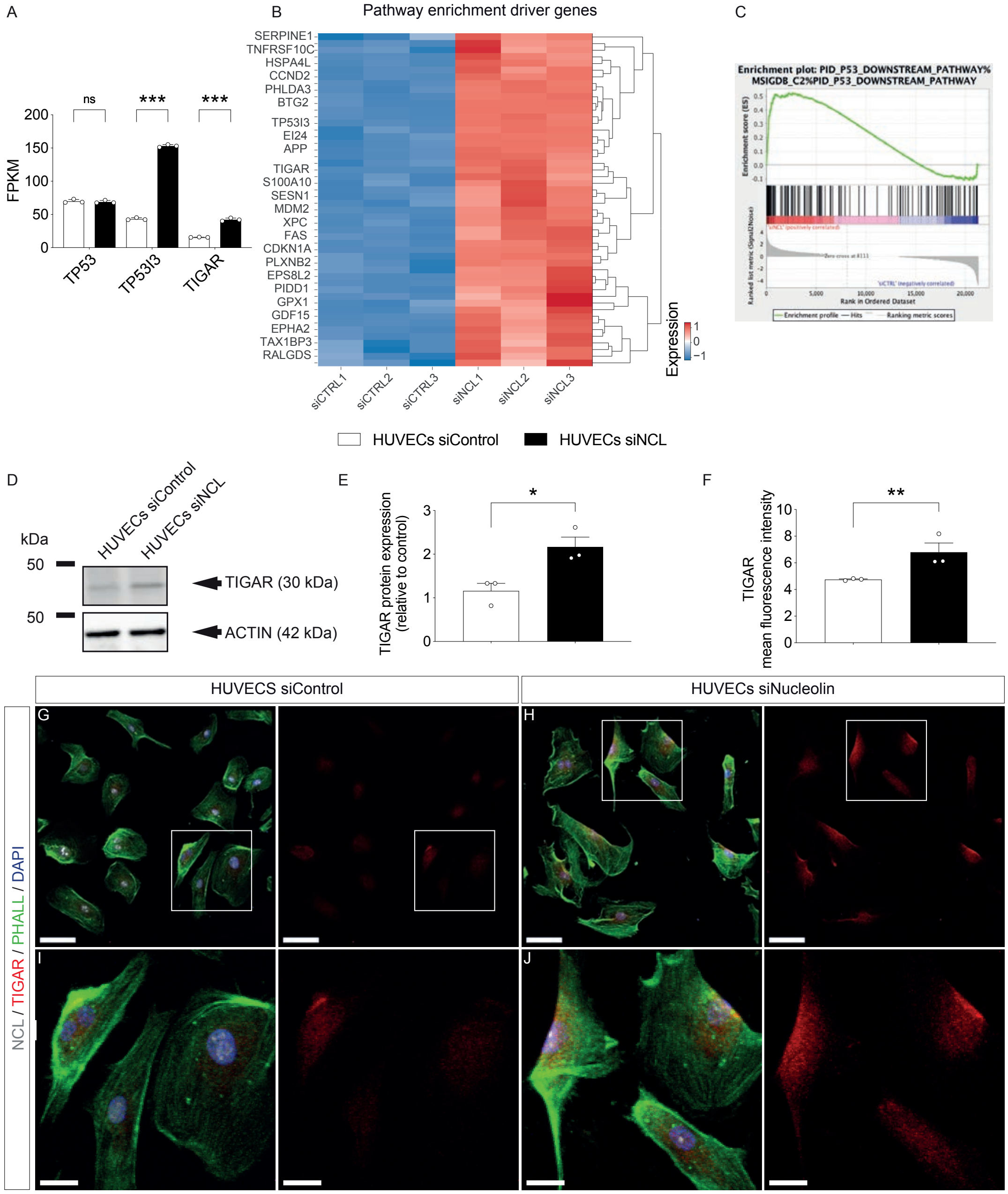

Supplementary Figure 7: Nucleolin positively regulates p53-TIGAR signaling pathway in HUVECs.

A Expression values (FPKM) of p53 signaling pathway genes. siNucleolin treatment induced upregulation of P53 target genes TP53IP and TIGAR without affecting P53 expression in HUVECs (n=3).

B-C TIGAR was among the top 50 genes driving the enrichment of p53 signaling pathway in HUVECs upon Nucleolin KD (**B**). Enrichment plot indicates that p53 signaling pathway is enriched in Nucleolin KD HUVECs as compared to the control (**C**).

D-E Western blot using antibody against TIGAR revealed an upregulation of TIGAR expression by Nucleolin knock-down in HUVECs (n=3).

F-J HUVECs were stained for Nucleolin (green), TIGAR (red), and the general nuclear marker DAPI (blue). TIGAR expression was upregulated in Nucleolin knock-down HUVECs as compared the HUVECs treated with a control siRNA (**F**, n=3).

Data represent mean ± SEM. For statistical analysis, Wald test corrected for multiple testing using the Benjamini and Hochberg method (**A**) and two-tailed unpaired Student's t -test (**E**,**F**) were performed $*P < 0.05$, $**P < 0.01$, $***P < 0.001$. The boxed areas (white box) in **G**,**H** are zoomed in **I**,**J** respectively. Scale bars: 70 µm in **G**,**H** and 20 µm in **I**,**J**.

Supplementary Figure 8

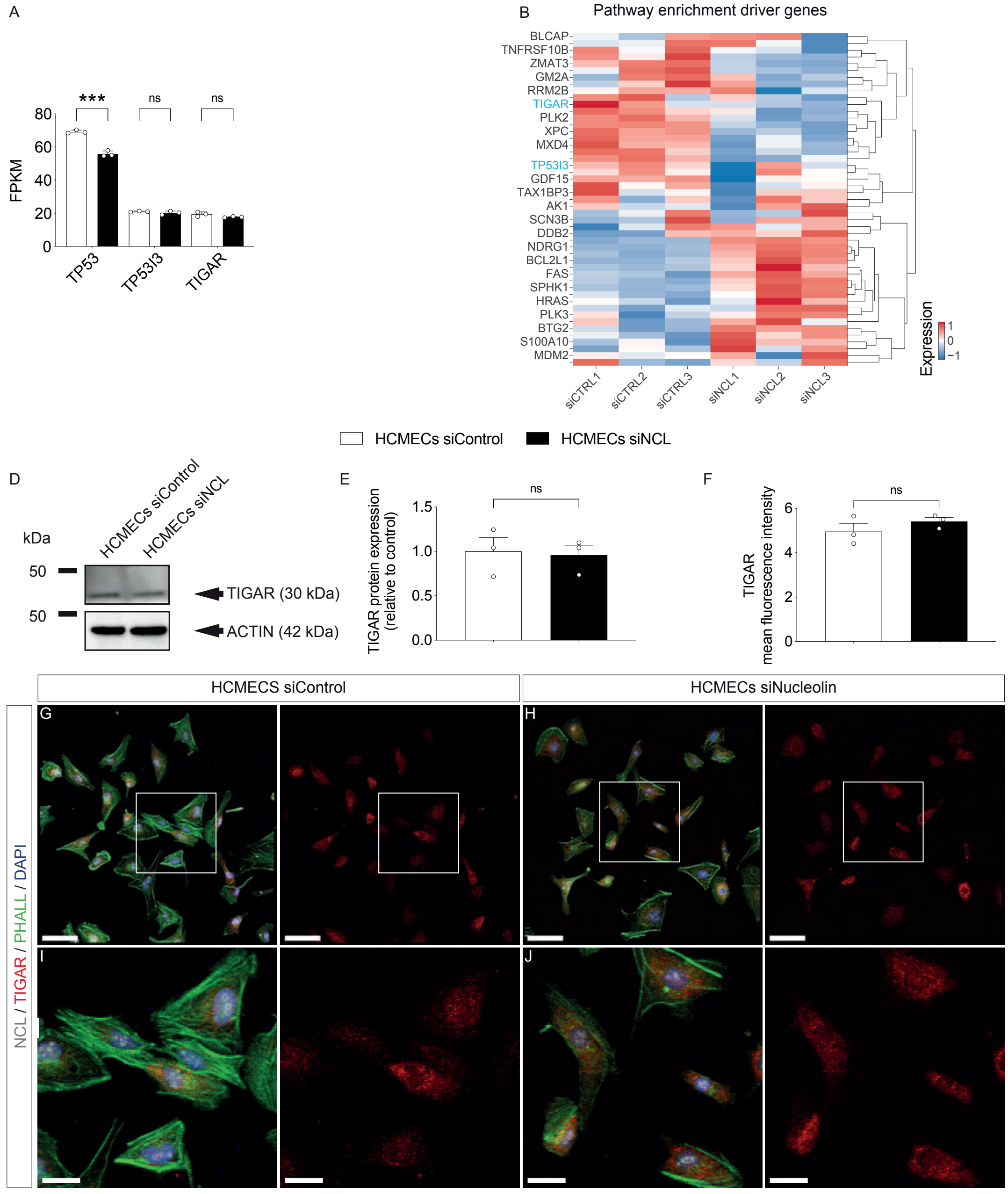

Supplementary Figure 8: Nucleolin does not regulate p53-TIGAR signaling pathway in HCMECs.

A Expression values (FPKM) of p53 signaling pathway genes. siNucleolin treatment induced downregulation of P53 without downstream genes TP53IP and TIGAR expression in HCMECs (n=3).

B Heatmap of the top 50 genes driving the enrichment of p53 signaling pathway in HUVEC$^{Nucleolin KD}$ showed different expression pattern in HCMECs as compared to HUVECs.

C-D Western blot using antibody against TIGAR showed no regulation of TIGAR expression by Nucleolin knock-down in HCMECs (n=3).

E-I HCMECs were stained for Nucleolin (green), TIGAR (red), and the general nuclear marker DAPI (blue). TIGAR expression was not regulated in Nucleolin knock-down HCMECs as compared the HCMECs treated with a control siRNA (**E**, n=3).

Data represent mean ± SEM. For statistical analysis, Wald test corrected for multiple testing using the Benjamini and Hochberg method (**A**) and two-tailed unpaired Student's t -test (**D**,**E**) were performed ***$P < 0.001$. The boxed areas (white box) in **F**,**G** are zoomed in **H**,**I** respectively. Scale bars: 70 µm in **F**,**G** and 20 µm in **H**,**I**.

Supplementary Figure 9

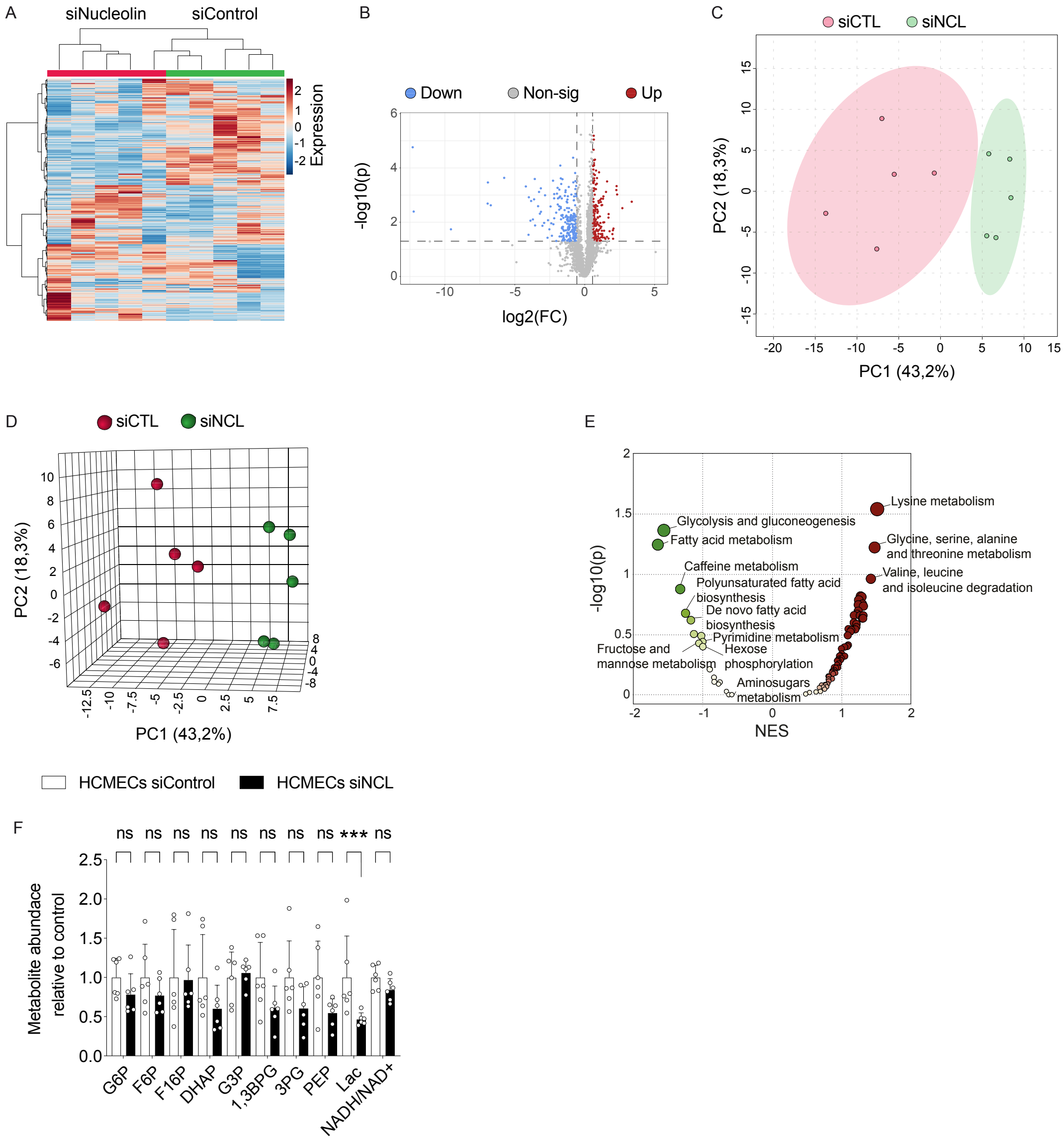

Supplementary Figure 9: Nucleolin regulates endothelial metabolism and alters metabolite profiles in HUVECs.

A-F Metabolite profile comparison between siRNA Nucleolin KD HUVEC and control HUVEC (n=5). Heatmap and hierarchical clustering showing 2000 differentially regulated metabolites (A). Scatter plot showing the significantly regulated metabolites upon Nucleolin KD. 165 metabolites were upregulated in HUVECs$^{\text{Nucleolin KD}}$ (indicated in red) and 218 were downregulated (indicated in blue) (**B**). Two-dimensional and three-dimensional PCA plot of log-transformed normalized concentration of 20000 metabolites, each dot represents a sample, and is colored by experimental group (**C**,**D**). Gene set enrichment analysis (GSEA), indicated a significant regulation of various metabolic pathways, including glycolysis in HUVECs upon Nucleolin KD (**E**). Relative abundance of glycolysis intermediates showed a downregulation of Lactate (Lac) upon Nucleolin KD (**F**, n=6). Levels Glucose-6-Phoshpate (G6P), Fructose-6-Phosphate (F6P), Fructose 1,6-bisphosphate (F16P), Dihydroxyacetone phosphate (DHAP), Glyceraldehyde 3-phosphate (G3P), 1,3-bisphosphoglycerate, 3-phosphoglycerate (3PG) and the ratio of NADH over NAD+ were not significantly regulated upon Nucleolin KD (**F**, n=6).

Data represent mean ± SEM. For statistical analysis, two-tailed unpaired Student's t -test were performed. *$P < 0.05$, **$P < 0.01$, ***$P < 0.001$.

Supplementary Figure 10

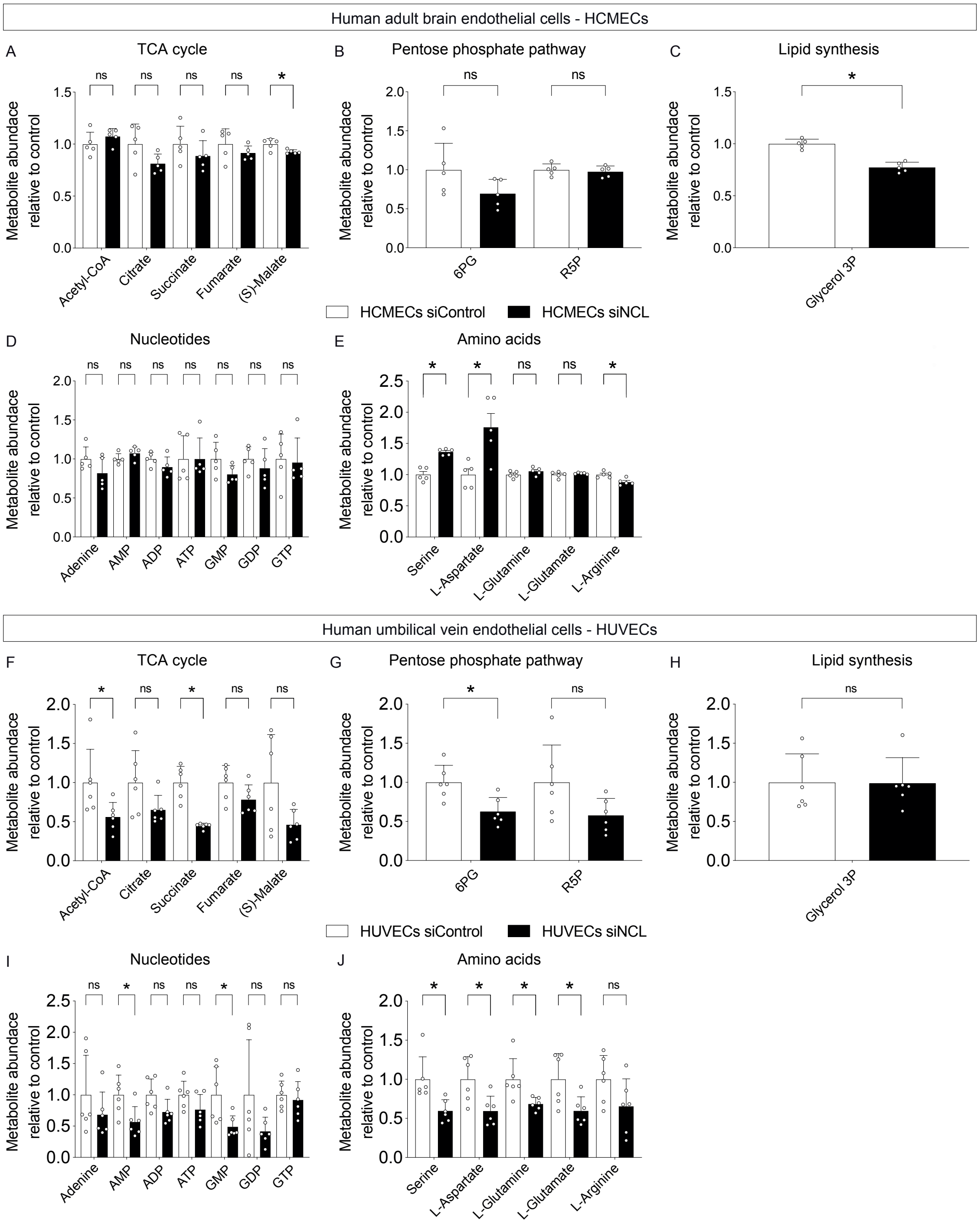

Supplementary Figure 10: Analysis of key metabolic intermediates using Liquid chromatography tandem mass spectrometry in brain and peripheral endothelial cells

(**A**-**E**) Abundance of key metabolites from selected endothelial metabolism pathways, such as the tricarboxylic acid (TCA) cycle, pentose phosphate pathway, lipid synthesis, nucleotides, and amino acids metabolism in HCMEC upon Nucleolin KD from 5 independent experiments. Levels of TCA cycle metabolites tend to decrease in HCMEC, with only Malate being significantly downregulated upon Nucleolin KD (**A**). No significant regulation of the pentose phosphate pathway as showed by 6-phosphogluconate (6PG) and ribose-5-phosphate (R5P) (**B**). Lipid synthesis intermediate sn-Glycerol-3-phosphate was downregulated upon Nucleolin KD (**C**). Nucleotides Adenine, AMP, ADP, GMP, GDP, and GTP showed no difference in their abundance between $HCMEC^{Nucleolin\ KD}$ and $HCMEC^{Control\ KD}$ (**D**). Abundance of Serine and L-Aspartate were significantly higher in HCMEC depleted from Nucleolin expression, while L-Arginine levels decreased in HCMEC upon Nucleolin KD (**E**).

(**F**-**J**) Abundance of key metabolites from selected endothelial metabolism pathways, such as the TCA cycle, pentose phosphate pathway, lipid synthesis, nucleotides, and amino acids metabolism in HUVEC upon Nucleolin KD from 6 independent experiments. Levels of TCA cycle metabolites tend to decrease in HUVECs, with Acetyl-CoA and Succinate being significantly downregulated upon Nucleolin KD (**F**). The pentose phosphate pathway was downregulated as showed by 6PG and R5P levels (**G**). Lipid synthesis intermediate sn-Glycerol-3-phosphate showed no regulation upon Nucleolin KD in HUVECs (**H**). Nucleotides levels tend to decrease with Adenine, AMP and GMP being significantly downregulated in $HUVEC^{Nucleolin\ KD}$ as compared to $HUVEC^{Control\ KD}$ (**I**). Abundance of amino acids Serine, L-Aspartate, L-Glutamine and L-Glutamate were significantly lower in HCMEC depleted from Nucleolin expression.

Data represent mean ± SEM. For statistical analysis, two-tailed unpaired Student's t -test were performed $^{*}P < 0.05$, $^{**}P < 0.01$, $^{***}P < 0.001$.

Supplementary Figure 11

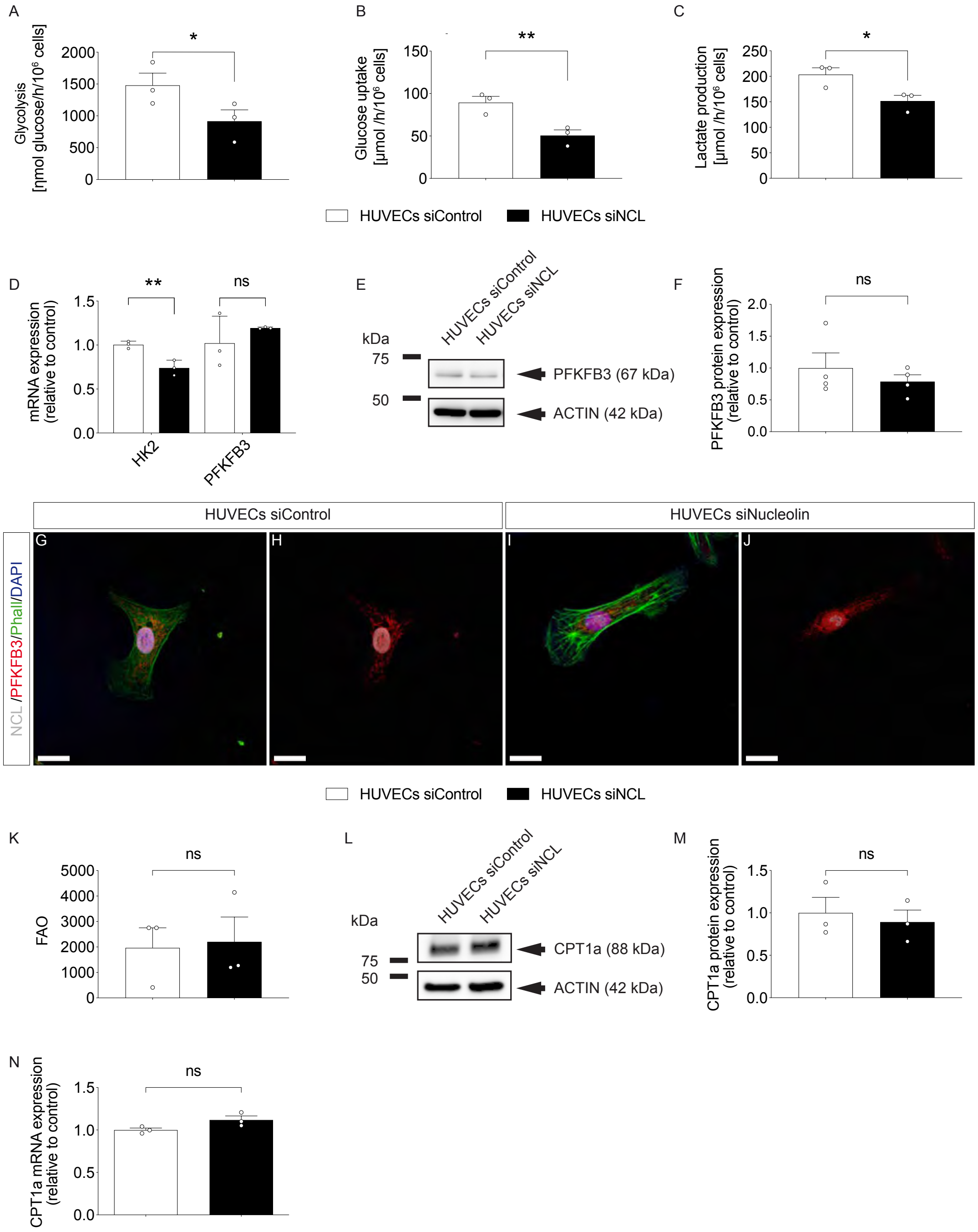

Supplementary Figure 11: Nucleolin positively regulates endothelial glucose metabolism via glycolytic enzymes including HK2 but does not affect fatty acid oxidation in HUVECs.

A-C Metabolic assays in HUVECs, upon Nucleolin downregulation with small interfering RNA targeting Nucleolin. Nucleolin knockdown decreased the glycolytic flux (**A**, n=3), glucose uptake (**B**, n=3) and lactate production (**C**, n=3) in HUVECs as compared to the tested controls.

D Quantitative RT-PCR revealing a significant downregulation of about 30% of Hexokinase-2 (*HK2*) mRNA expression by siRNA-targeted Nucleolin knock-down. 6-phosphofructo-2-kinase/fructose-2,6-biphosphatase 3 (*PFKFB3*) expression showed slight but no significant increase (n=3).

E-F Western blot using antibody against PFKFB3 revealed no significant regulation of PFKFB3 expression by Nucleolin knock-down in HUVECs.

G-J HUVECs were stained for Nucleolin (green), PFKFB3 (red), and the general nuclear marker DAPI (blue). No difference in PFKFB3 expression could be seen between Nucleolin knock-down HUVECs (**G**,**H**) and the HUVECs treated with a control siRNA (**I,J**).

K Nucleolin knock-down in HUVECs did not affect fatty acid oxidation (n=3).

L-M Western blot using antibodies against the carnitine palmitoyltransferase 1A (CPT1a) showed no difference in CPT1a protein expression between Nucleolin knock-down HUVECs and the control condition (n=3).

N Quantitative RT-PCR showed no significant regulation of (*CPT1A*) mRNA expression upon siRNA-targeted Nucleolin knock-down (n=3).

Data represent mean ± SEM. For statistical analysis, two-tailed unpaired Student's t -test were performed. *$P < 0.05$, **$P < 0.01$, ***$P < 0.001$. Scale bars: 20 µm in **G-J**
